# Cryptococcus neoformans*-*Infected Macrophages Release Proinflammatory Extracellular Vesicles: Insight into Their Components by Multi-omics

**DOI:** 10.1128/mBio.00279-21

**Published:** 2021-03-30

**Authors:** Lei Zhang, Keming Zhang, Hang Li, Carolina Coelho, Diego de Souza Gonçalves, Man Shun Fu, Xinhua Li, Ernesto S. Nakayasu, Young-Mo Kim, Wanqing Liao, Weihua Pan, Arturo Casadevall

**Affiliations:** a Department of Dermatology and Venereology, Changzheng Hospital, Second Military Medical University, Shanghai, China; b Shanghai Key Laboratory of Molecular Medical Mycology, Shanghai Institute of Medical Mycology, Changzheng Hospital, Second Military Medical University, Shanghai, China; c Department of Dermatology, Shaanxi Provincial People’s Hospital, Xi’an, China; d MRC Centre for Medical Mycology, University of Exeter, Exeter, United Kingdom; e Faculdade de Medicina, Universidade Federal do Rio de Janeiro, Pós-Graduaçao em Doença Infecciosas e Parasitárias, Rio de Janeiro, Brazil; f Institute of Immunology and Immunotherapy, Institute of Biomedical Research College of Medical and Dental Science, Birmingham, United Kingdom; g Department of Dermatology and Venereology, Taiyuan Central Hospital of Shanxi Medical University, Shanxi, China; h Biological Sciences Division, Pacific Northwest National Laboratory, Richland, Washington, USA; i Department of Molecular Microbiology and Immunology, Johns Hopkins School of Public Health, Baltimore, Maryland, USA; Institut Pasteur; Universidad de Córdoba

**Keywords:** extracellular vesicles, *Cryptococcus neoformans*, proteomics, lipidomics, metabolomics

## Abstract

Cryptococcus neoformans causes deadly mycosis in immunocompromised individuals. Macrophages are key cells fighting against microbes. Extracellular vesicles (EVs) are cell-to-cell communication mediators. The roles of EVs from infected host cells in the interaction with *Cryptococcus* remain uninvestigated. Here, EVs from viable C. neoformans-infected macrophages reduced fungal burdens but led to shorter survival of infected mice. *In vitro*, EVs induced naive macrophages to an inflammatory phenotype. Transcriptome analysis showed that EVs from viable C. neoformans-infected macrophages activated immune-related pathways, including p53 in naive human and murine macrophages. Conserved analysis demonstrated that basic cell biological processes, including cell cycle and division, were activated by infection-derived EVs from both murine and human infected macrophages. Combined proteomics, lipidomics, and metabolomics of EVs from infected macrophages showed regulation of pathways such as extracellular matrix (ECM) receptors and phosphatidylcholine. This form of intermacrophage communication could serve to prepare cells at more distant sites of infection to resist C. neoformans infection.

## Abstract (precis)

Cryptococcus neoformans causes cryptococcal meningitis, which is frequent in patients with HIV/AIDS, especially in less-developed countries. The incidence of cryptococcal meningitis is close to 1 million each year globally.

## INTRODUCTION

Cryptococcosis, a disease manifested mainly by pneumonia and meningitis, is caused primarily by Cryptococcus neoformans (1). The incidence of cryptococcal meningitis is estimated to be 223,100 cases per year globally (2). Humans are infected primarily through inhalation of desiccated yeast. In individuals with intact immunity, infection is usually asymptomatic and contained in the lung. If the infection is not cleared, a state of latency is established, which can reactivate when immunity is subsequently impaired (3). Moreover, the case fatality rate is estimated to be as high as 75% among persons living with HIV/AIDS in less-developed regions (2). Therefore, the progress and outcome in C. neoformans infection depend on the interplay between C. neoformans and a healthy host immune response.

Macrophages are the main phagocytic cells during C. neoformans infection. As the first line of defense, macrophages play a fundamental role in the control of C. neoformans infection (4). On the one hand, macrophages with M1 polarization can effectively inhibit the spread of C. neoformans (5). Murine bone marrow-derived macrophages (BMDMs) stimulated with gamma interferon (IFN-γ) effectively suppressed the growth of C. neoformans, and the effect of IFN-γ was augmented by lipopolysaccharide (LPS) (6). On the other hand, C. neoformans can survive and reproduce inside macrophages via several mechanisms such as inducing macrophage lysosome damage, modulating phagosomal pH, and causing alterations in the host cell cycle (7, 8). Consequently, macrophages can be a niche for C. neoformans to survive and spread. However, the mechanisms by which intracellular C. neoformans modulates macrophage functions remain poorly understood, especially at distant sites of infection.

Extracellular vesicles (EVs), or exosomes, are membrane-bound vesicles released by diverse types of cells that differ in size, ranging from 30 to 1,000 nm in diameter, although most are in the range of 100 to 150 nm (9). EVs are a vital mode for intercellular communication since vesicles function as vehicles for the transfer of materials such as proteins, lipids, and RNAs (10). C. neoformans-derived EVs contain components associated with virulence, and their packaging into vesicles allows the delivery of a concentrated payload of fungal products to recipient cells (11). C. neoformans-derived EVs can also stimulate macrophage inflammatory responses, including tumor necrosis factor alpha (TNF-α) and interleukin-10 (IL-10) production, which enhance macrophage fungicidal activity (12). The use of EVs obtained from a C. neoformans acapsular strain prolonged the survival of mice upon C. neoformans infection (13).

Apart from pathogen-derived vesicles, infected host cells can also produce EVs that can mediate the host immune response (14). Although this topic has been investigated for bacterial and parasitic pathogenic microbes, little work has been done with fungal pathogens. THP-1-derived EVs from cells infected with Candida albicans activate extracellular signal-regulated kinase (ERK) and p38 kinases to increase both the secretion of proinflammatory cytokines and candidacidal activity (15). Given this observation, there is a high likelihood that macrophage-derived exosomes are important in other fungal diseases, but little is known about the composition of host-derived EVs and their effects on recipient cells in the context of fungal pathogenesis.

In the present study, we first tested the biological effects of EVs secreted by activated BMDMs after exposure to C. neoformans both *in vivo* and *in vitro*. The underlying signaling pathways triggered by EVs in naive macrophages were investigated using transcriptional analysis. Finally, the components of vesicles were identified by a multi-omics technique. Our results show that during C. neoformans infection, EVs secreted by host activated macrophages could serve as a “priming” signal to shift naive macrophages to a proinflammatory phenotype and increase macrophage fungicidal activity.

## RESULTS

### EVs from C. neoformans-infected BMDMs lower the fungal burden but decrease mouse survival in murine experimental cryptococcosis.

**FIG 1:**
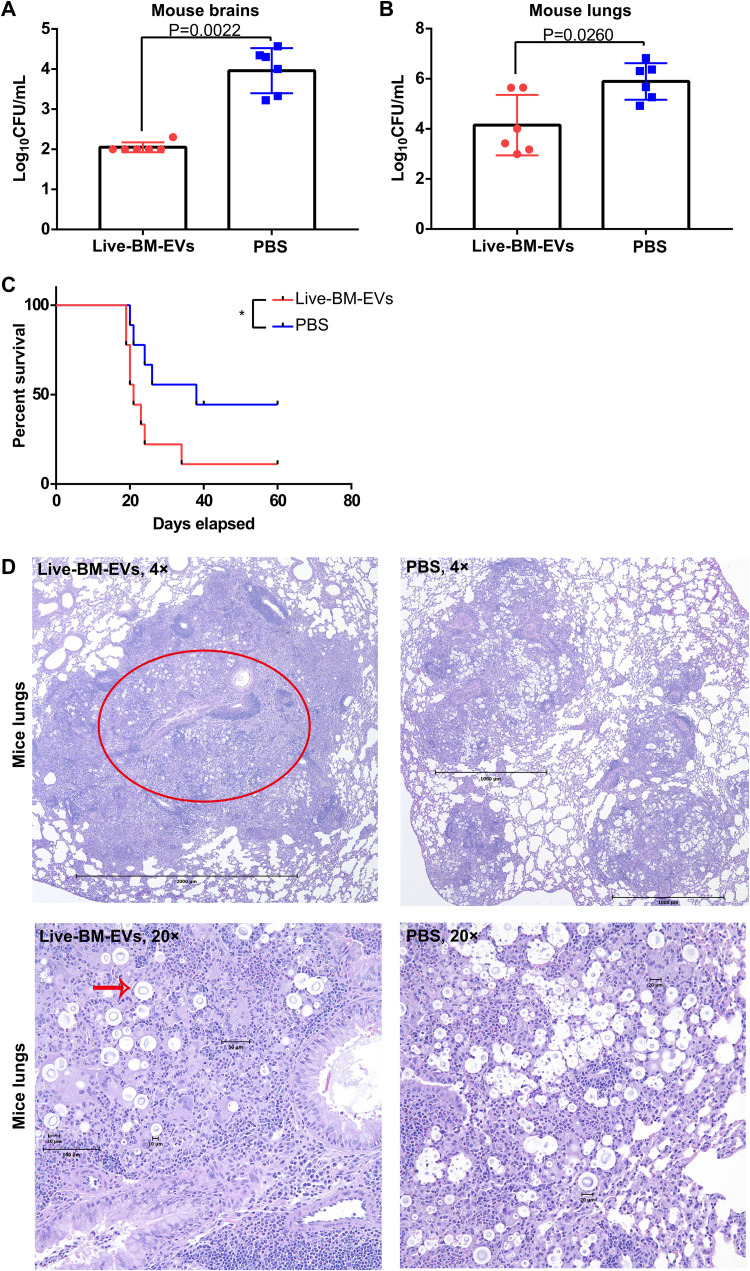
Administration of live-BM-EVs (EVs from live C. neoformans-infected activated BMDMs) decreased fungal burdens in both lungs and brains but led to shorter survival in murine experimental cryptococcosis. (A) CFU of mouse brains in EV-treated and nontreated groups. (B) CFU of mouse lungs in EV-treated and nontreated groups. Each point represents CFU from one mouse. (C) Survival analysis of EV-treated and nontreated groups (*n* = 9). *, *P* < 0.05. (D) Representative histopathology of lungs in EV-treated and nontreated groups. The red circle indicates a granuloma in an infected lung, and the red arrow points at cryptococcal giant cells. Data are shown as the means ± SD from at least six mice per group. An unpaired *t* test was used to calculate *P* values. A log rank (Mantel-Cox) test was used to compare the survival differences.

In preliminary experiments, we observed that EVs from BMDMs infected with live C. neoformans (live-BM-EVs) regulated macrophage polarization to an M1 phenotype *in vitro*. Thus, we explored their potential *in vivo* effects in a murine experimental cryptococcosis model (Fig. 1). Briefly, C57BL/6 mice were injected intraperitoneally (i.p.) with live-BM-EVs 1 day before intranasal infection with live C. neoformans. We monitored disease progression and fungal burdens 2 weeks after infection. CFU in both lungs and brains were lower in the group treated with live-BM-EVs (Fig. 1A and B). Surprisingly, despite the lower fungal burden, earlier mouse death was observed in the group treated with live-BM-EVs (Fig. 1C). In histopathological analyses of lungs (Fig. 1D), more extensive pyogranulomatous inflammation (red circle) with numerous giant cells (red arrow) and phagocytosis was observed in the lungs of the live-BM-EV-treated group than in the phosphate-buffered saline (PBS)-treated group. In histopathological analyses of brains, no significant differences were detected.

### EVs from activated BMDMs regulated the naive BMDM shift to the M1 phenotype.

**FIG 2:**
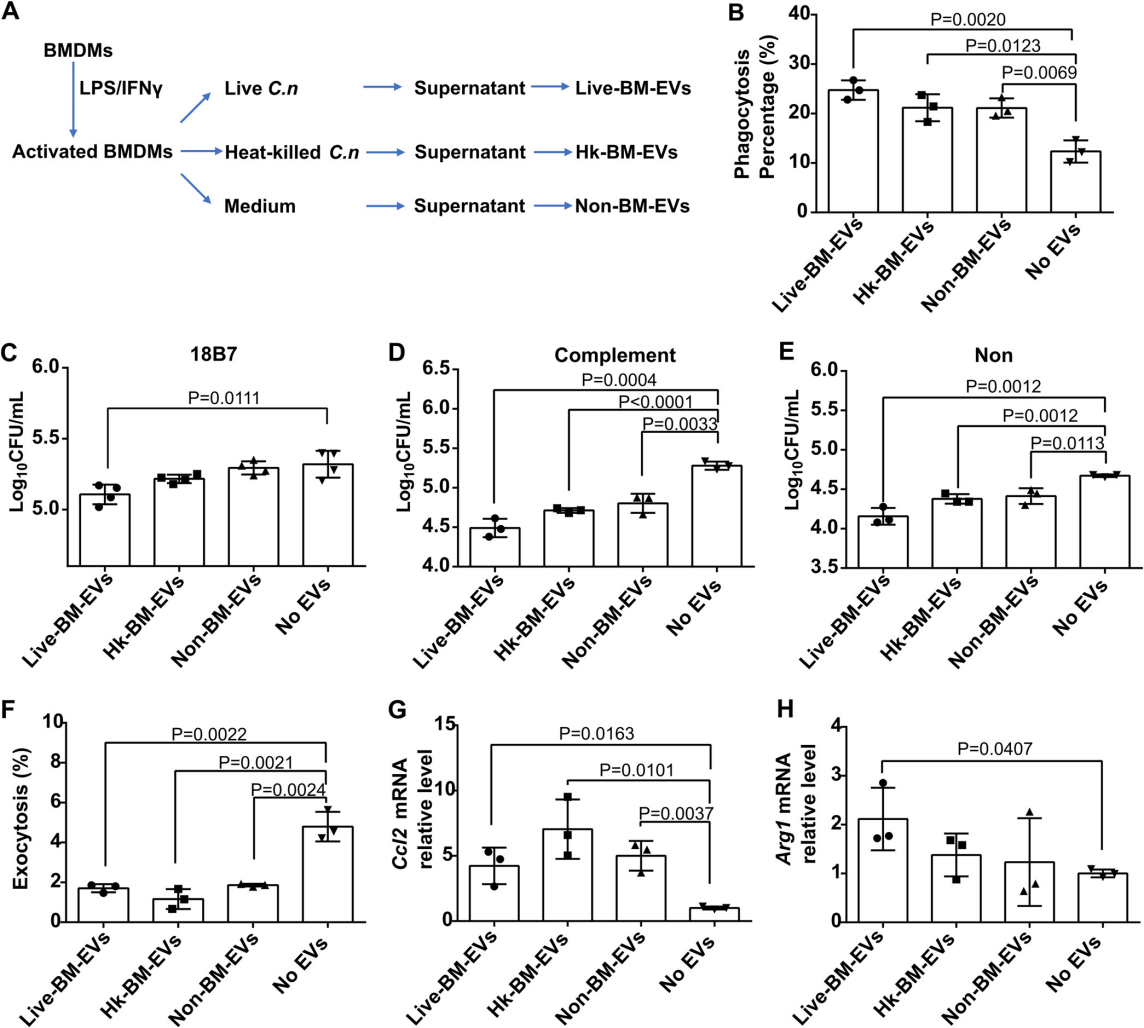
All three types of activated BMDM-EVs triggered increased antifungal activity of naive BMDMs during C. neoformans (*C.n*) infection, while live-BM-EVs (EVs from live C. neoformans-infected activated BMDMs) had the highest potential. (A) Flowchart of harvesting of the three types of EV samples. (B) Phagocytosis percentages of naive BMDMs after incubation with three types of EV samples. At least 100 macrophages were counted for each group. (C to E) Fungicidal activity of naive BMDMs after incubation with three types of EV samples with opsonin 18B7 (C), complement (D), or none (E). (F) Nonlytic exocytosis of naive BMDMs after incubation with three types of EV samples. (G and H) Macrophage polarization, as measured by mRNA levels of *Ccl2* (G) and *Arg1* (H), in naive BMDMs after incubation with three types of EV samples. Non-BM-EVs, EVs from activated BMDMs without C. neoformans infection; No EVs, EV-nontreated macrophages; Hk, heat killed. Data are shown as the means ± SD from at least three independent experiments, each performed in triplicate. An unpaired *t* test was used to calculate *P* values.

Next, we examined whether activated BMDM-derived EVs had biological effects on BMDMs *in vitro*. To investigate whether viable and dead C. neoformans cells could have similar effects, different types of EV samples were harvested from activated BMDMs (Fig. 2A). Naive macrophages were treated with three types of EV samples; EV-nontreated naive BMDMs were used as the control. The incorporation of these EVs by naive BMDMs happened by as early as 30 min and increased over time (observed by confocal microscopy) (see Fig. S1 in the supplemental material). Next, we examined whether these ingested EVs affected the antifungal effects of BMDMs. Inducible nitrate production in macrophages is a vital microbicidal mechanism (16). Therefore, after coincubation with EVs for 24 h, the supernatant nitrate concentration was determined. None of the three types of EV samples had an effect on the nitrate levels in the supernatant of macrophages (Table S1). All three types of EV samples enhanced the efficiency of phagocytosis, with live-BM-EVs causing the strongest increase in the phagocytosis percentage (Fig. 2B). For the killing function of macrophages, independent of the opsonin used to promote fungal ingestion, all EV-treated macrophages showed a higher killing capacity than nontreated macrophages (Fig. 2C to E). To ascertain the effect of EVs on nonlytic exocytosis, all events in 12 h using time-lapse microscopy were counted. Each of the three types of EV samples significantly suppressed the nonlytic exocytosis of macrophages (Fig. 2F). To assess the polarization of macrophages, we chose representative markers of classically (*Ccl2*) and alternatively (*Arg1*) activated macrophages. Both *Ccl2* and *Arg1* increased in macrophages after coincubation with EVs, with the increase of *Ccl2* being higher than that of *Arg1* (Fig. 2G and H). Compared with non-BM-EVs (EVs from activated BMDMs without C. neoformans infection), these *in vitro* effects on macrophages between C. neoformans-infected BM-EVs and non-BM-EVs were comparable except for their fungicidal effects, which were statistically different between live-BM-EVs and non-BM-EVs, independent of opsonin (Table S2).

10.1128/mBio.00279-21.1 FIG S1 Incorporation of three types of activated BMDM-EV samples by naive BMDMs analyzed by confocal microscopy. Red fluorescence represents vesicles with DiI staining, green fluorescence represents a cell membrane with CtxB-Alexa Fluor staining, and blue fluorescence represents a nucleus with DAPI staining. Live-EVs, inactivated BMDMs incubated with EVs from live C. neoformans-infected activated BMDMs; hk-EVs, inactivated BMDMs with EVs from heat-killed C. neoformans-infected activated BMDMs; nc-EVs, inactivated BMDMs with EVs from noninfected activated BMDMs. Download FIG S1, DOCX file, 0.3 MB. Copyright © 2021 Zhang et al. 2021 Zhang et al. https://creativecommons.org/licenses/by/4.0/ This content is distributed under the terms of the Creative Commons Attribution 4.0 International license.

10.1128/mBio.00279-21.5 TABLE S1 Nitrite concentrations of naive BMDMs after incubation with three types of EV samples. Download Table S1, DOCX file, 0.02 MB. Copyright © 2021 Zhang et al. 2021 Zhang et al. https://creativecommons.org/licenses/by/4.0/ This content is distributed under the terms of the Creative Commons Attribution 4.0 International license.

10.1128/mBio.00279-21.6 TABLE S2 Statistical difference comparison of *in vitro* effects between C. neoformans-activated BM-EVs and non-BM-EVs (EVs from activated BMDMs without C. neoformans infection). Live-BM-EVs, EVs from live C. neoformans-infected activated BMDMs; Hk-BM-EVs, EVs from heat-killed C. neoformans-infected activated BMDMs. An unpaired *t* test was used to calculate *P* values. Download Table S2, DOCX file, 0.02 MB. Copyright © 2021 Zhang et al. 2021 Zhang et al. https://creativecommons.org/licenses/by/4.0/ This content is distributed under the terms of the Creative Commons Attribution 4.0 International license.

### Immune-related pathways in naive BMDMs were activated by live-BM-EVs.

**FIG 3:**
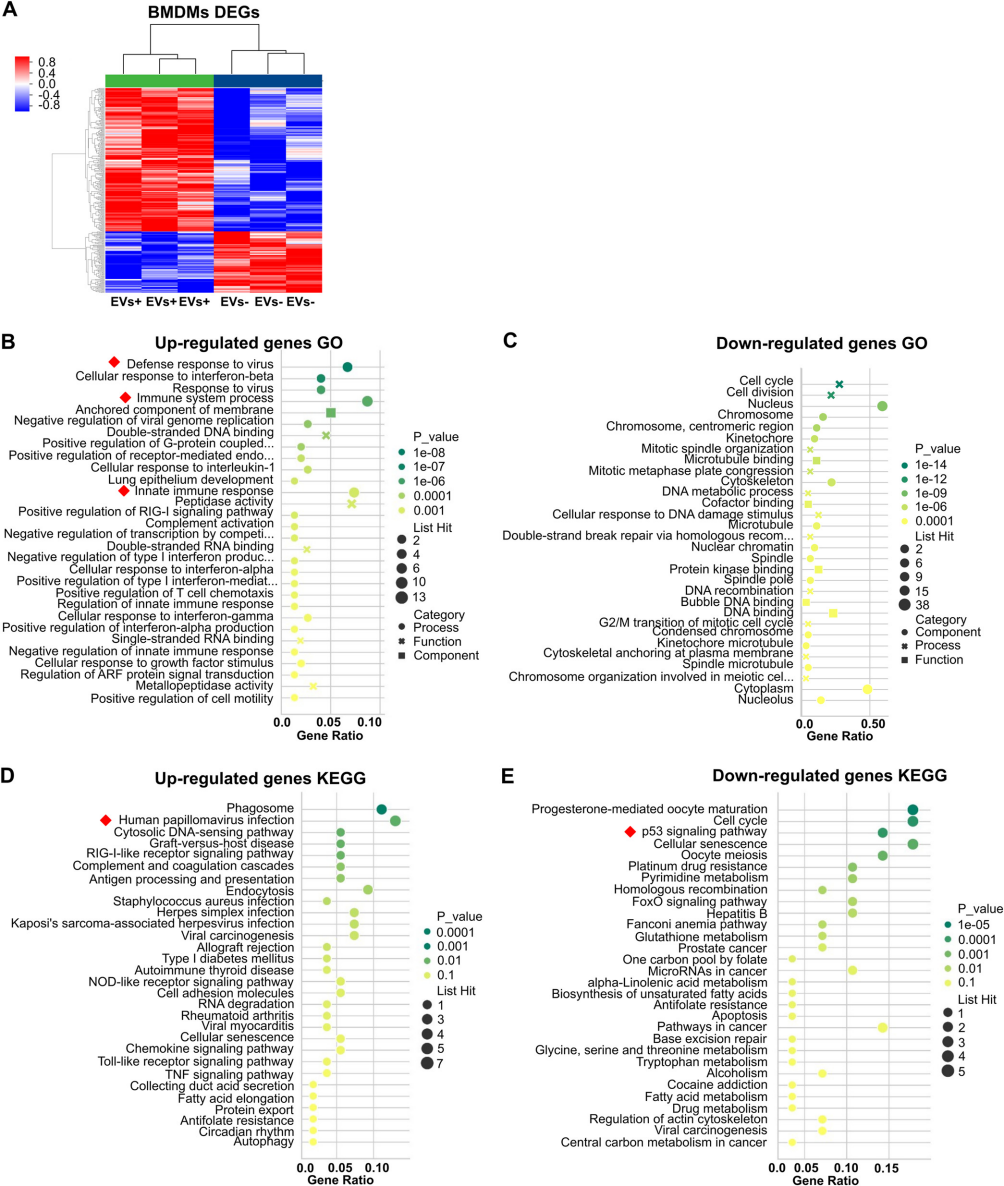
Transcriptional changes in naive BMDMs incubated with live-BM-EVs. EV-nontreated BMDMs were used as a control. (A) Volcano plot of differentially expressed genes (DEGs) in EV-treated and untreated groups. Red, upregulated; blue, downregulated (*n* = 3 for each group). (B and C) GO analysis of upregulated (B) and downregulated (C) DEGs. (D and E) KEGG analysis of upregulated (D) and downregulated (E) DEGs. Red squares indicate immune-related pathways.

To investigate the underlying genes and pathways involved in the naive macrophage response to sensing EVs, transcriptome analysis of naive macrophages after treatment with EVs was performed (EV-nontreated BMDMs were used as a control). Using a fold change of at least 1.5 and a *P* value of <0.05 (for *P* value calculations, see Materials and Methods) as cutoffs, there were 220 upregulated and 94 downregulated genes in naive BMDMs incubated with live-BM-EVs in comparison with BM-EV-nontreated BMDMs (Fig. 3A; transcriptome analyses of naive BMDMs incubated with Hk-BM-EVs [EVs from heat-killed C. neoformans-infected activated BMDMs] and non-BM-EVs are presented in Fig. S2). Gene Ontology (GO) (Fig. 3B and C), widely used in functional annotation and enrichment analyses (17), and Kyoto Encyclopedia of Genes and Genomes (KEGG) (Fig. 3D and E) analysis revealed high enrichment of immune-related pathway (Fig. 3D and E, red squares), including GO terms such as “defense response to virus,” “immune system process,” and “innate immune response,” and pathways such as “human papillomavirus infection” and “p53 signaling.”

10.1128/mBio.00279-21.2 FIG S2 Transcriptome analysis of naive BMDMs incubated with Hk-BM-EVs (EVs from heat-killed C. neoformans-infected BMDMs) (A) and non-BM-EVs (EVs from activated BMDMs without infection) (B). Red, upregulated; blue, downregulated (*n* = 3 for each group). (A) Using a fold change of at least 1.5 and a *P* value of <0.05 as cutoffs, there were 319 upregulated and 487 downregulated genes in naive BMDMs incubated with Hk-BM-EVs in comparison with BM-EV-nontreated BMDMs. (B) In naive BMDMs incubated with non-BM-EVs, there were 105 upregulated and 123 downregulated genes compared to BM-EV-nontreated BMDMs. EVs−, EV nontreated naive macrophages. Download FIG S2, DOCX file, 0.4 MB. Copyright © 2021 Zhang et al. 2021 Zhang et al. https://creativecommons.org/licenses/by/4.0/ This content is distributed under the terms of the Creative Commons Attribution 4.0 International license.

### Basic cell biological processes, including cell cycle and division, were the shared targets of EVs from both murine and human infected macrophages.

**FIG 4:**
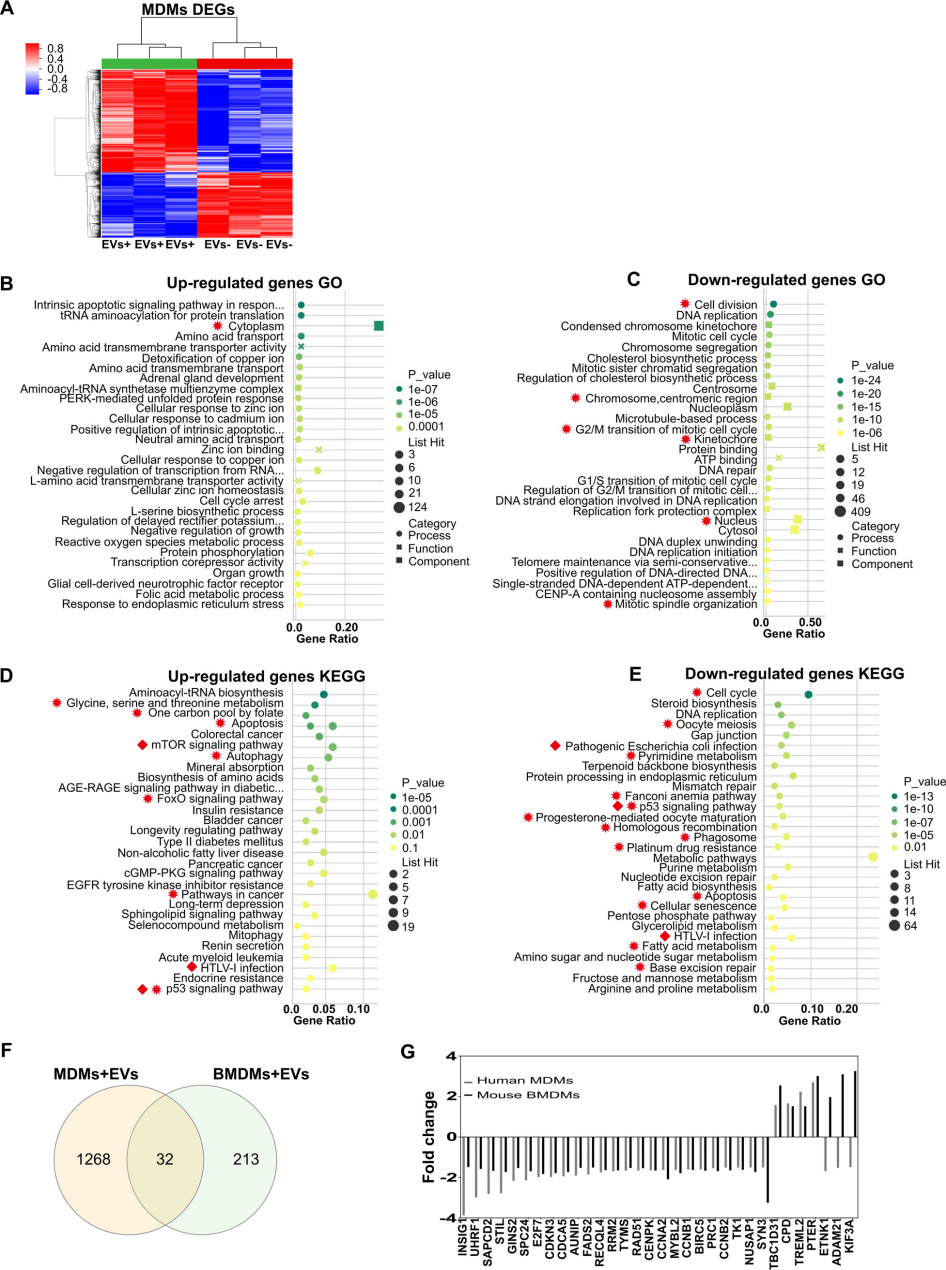
Transcriptional changes of naive human MDMs (monocyte-derived macrophages) incubated with live-M-EVs (EVs from live C. neoformans-infected activated human peripheral MDMs). (A) Differentially expressed genes (DEGs) in EV-treated and untreated groups. Red, upregulated; blue, downregulated (*n* = 3 for each group). (B and C) GO analysis of upregulated (B) and downregulated (C) DEGs. (D and E) KEGG analysis of upregulated (D) and downregulated (E) DEGs. EGFR, epidermal growth factor receptor. (F) Shared DEGs between MDMs and BMDMs identified by Venn analysis. (G) Fold changes of shared DEGs between MDMs and BMDMs. Red squares indicate immune-related pathways. Red asterisks indicate shared GO and KEGG pathways.

To expand the scope of our findings to human macrophages, transcriptome analysis was performed in naive peripheral blood monocyte-derived macrophages (MDMs) treated with EVs from live C. neoformans-infected activated MDMs (live-M-EVs). Using fold changes of at least 1.5 and *P* values of <0.05 as cutoffs, there were 599 upregulated and 942 downregulated genes in naive MDMs incubated with live-M-EVs, compared with EV-nontreated MDMs (Fig. 4A; transcriptome analyses of naive MDMs incubated with Hk-M-EVs and non-M-EVs are presented in Fig. S3). In GO (Fig. 4B and C) and KEGG (Fig. 4D and E) analyses, the most enriched GOs were cytoplasm and protein binding, pathways in cancer, and metabolic pathways. Immune-related pathways, including “p53 signaling pathway,” “mTOR signaling pathways,” and “human T-cell leukemia virus, type 1 (HTLV-1) infection,” were observed (Fig. 4D and E, red squares). To identify the shared effects of infected macrophage EVs, we performed a conserved analysis based on the transcriptome data from both murine and human macrophages treated with either live-BM-EVs or live-M-EVs. Thirty-two common differentially expressed genes (DEGs) between human and mouse macrophages were identified as shared targets (Fig. 4F and G). For GO conserved analysis, one upregulated and six downregulated genes were identified as shared GO pathways (Fig. 4B and C, red asterisks). For KEGG conserved analysis, 7 upregulated and 13 downregulated genes were identified as shared pathways (Fig. 4D and E, red asterisks). Basic cell biological processes, including cell cycle and division, were the shared targets of EVs from both murine and human infected macrophages.

10.1128/mBio.00279-21.3 FIG S3 Transcriptome analysis of naive MDMs incubated with Hk-M-EVs (EVs from heat-killed C. neoformans-infected activated human peripheral MDMs) (A) and non-M-EVs (EVs from activated human peripheral MDMs without infection) (B). Red, upregulated; blue, downregulated (*n* = 3 for each group). (A) Using fold changes of at least 1.5 and *P* values of <0.05 as cutoffs, there were 2,617 upregulated and 2,758 downregulated genes in naive MDMs incubated with Hk-M-EVs, compared with EV-nontreated MDMs. (B) In naive MDMs incubated with non-M-EVs, there were 759 upregulated and 1,077 downregulated genes compared to EV-nontreated MDMs. EVs−, EV nontreated naive macrophages. Download FIG S3, DOCX file, 0.4 MB. Copyright © 2021 Zhang et al. 2021 Zhang et al. https://creativecommons.org/licenses/by/4.0/ This content is distributed under the terms of the Creative Commons Attribution 4.0 International license.

### The main size of EVs secreted by BMDMs was 40 to 60 nm in diameter.

**FIG 5:**
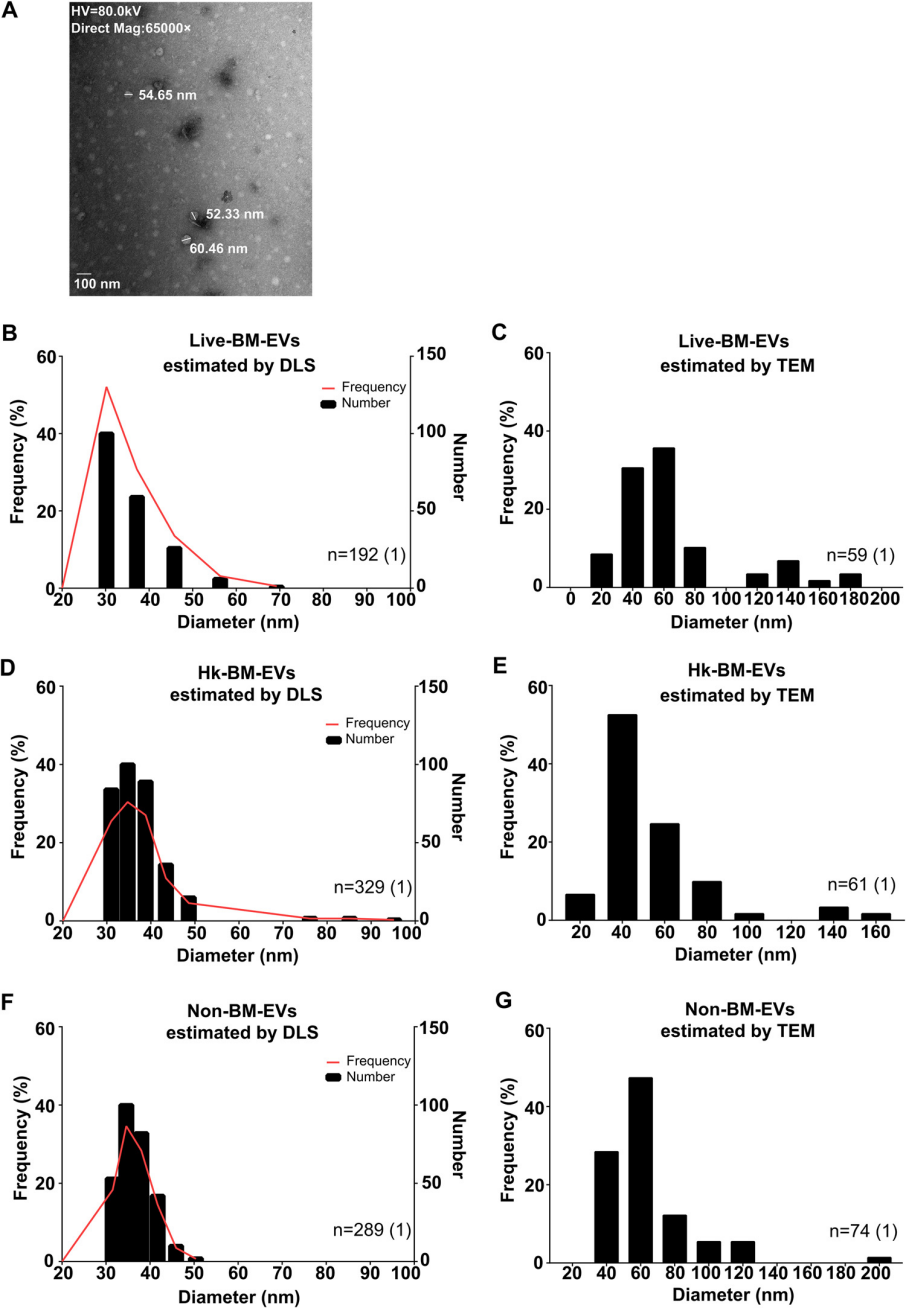
Size distributions of BMDM-EVs under different cryptococcal stimulations. The major size distribution of BMDM-EVs was around 40 to 60 nm in diameter. (A) Representative TEM image of BMDM-EVs. (B and C) Frequency distributions of live-BM-EVs (EVs from live C. neoformans-infected activated BMDMs) analyzed by DLS (B) (*n* = 192) and TEM (C) (*n* = 59). (D and E) Frequency distributions of Hk-BM-EVs (EVs from heat-killed C. neoformans-infected activated BMDMs) analyzed by DLS (D) (*n* = 329) and TEM (E) (*n* = 61). (F and G) Frequency distributions of non-BM-EVs (EVs from activated BMDMs without C. neoformans infection) analyzed by DLS (F) (*n* = 289) and TEM (G) (*n* = 74). HV, high voltage.

The physical features and components of three types of EV samples were identified by dynamic light scattering (DLS), transmission electron microscopy (TEM), and multi-omics analyses. First, the particle size characteristics were determined by DLS, which provides information on the mean size of vesicles and the width of the size distribution (18), and TEM. The main distributions of the three types of EV samples were similar, around 40 to 60 nm (Fig. 5). DLS showed a main size of around 40 nm in diameter (Fig. 5B, D, and F), while TEM demonstrated a main distribution of 60 nm in diameter (Fig. 5C, E, and G). The size distributions of EVs from naive BMDMs (without LPS or IFN-γ treatment) were also assessed by DLS and TEM (Fig. S4), and a similar distribution of around 40 to 60 nm was observed.

10.1128/mBio.00279-21.4 FIG S4 Frequency distributions of EVs from naive macrophages under different cryptococcal stimulations. (A and B) Frequency distributions of live-BM-EVs (EVs from live C. neoformans-infected activated BMDMs) analyzed by DLS (A) and TEM (B). (C and D) Frequency distributions of Hk-BM-EVs (EVs from heat-killed C. neoformans-infected activated BMDMs) analyzed by DLS (C) and TEM (D). (E and F) Frequency distributions of non-BM-EVs (EVs from activated BMDMs without C. neoformans infection) analyzed by DLS (E) and TEM (F). Download FIG S4, DOCX file, 0.2 MB. Copyright © 2021 Zhang et al. 2021 Zhang et al. https://creativecommons.org/licenses/by/4.0/ This content is distributed under the terms of the Creative Commons Attribution 4.0 International license.

### Proteins and lipids, other than metabolites, are potential signaling mediators in BMDM-derived EVs.

**FIG 6:**
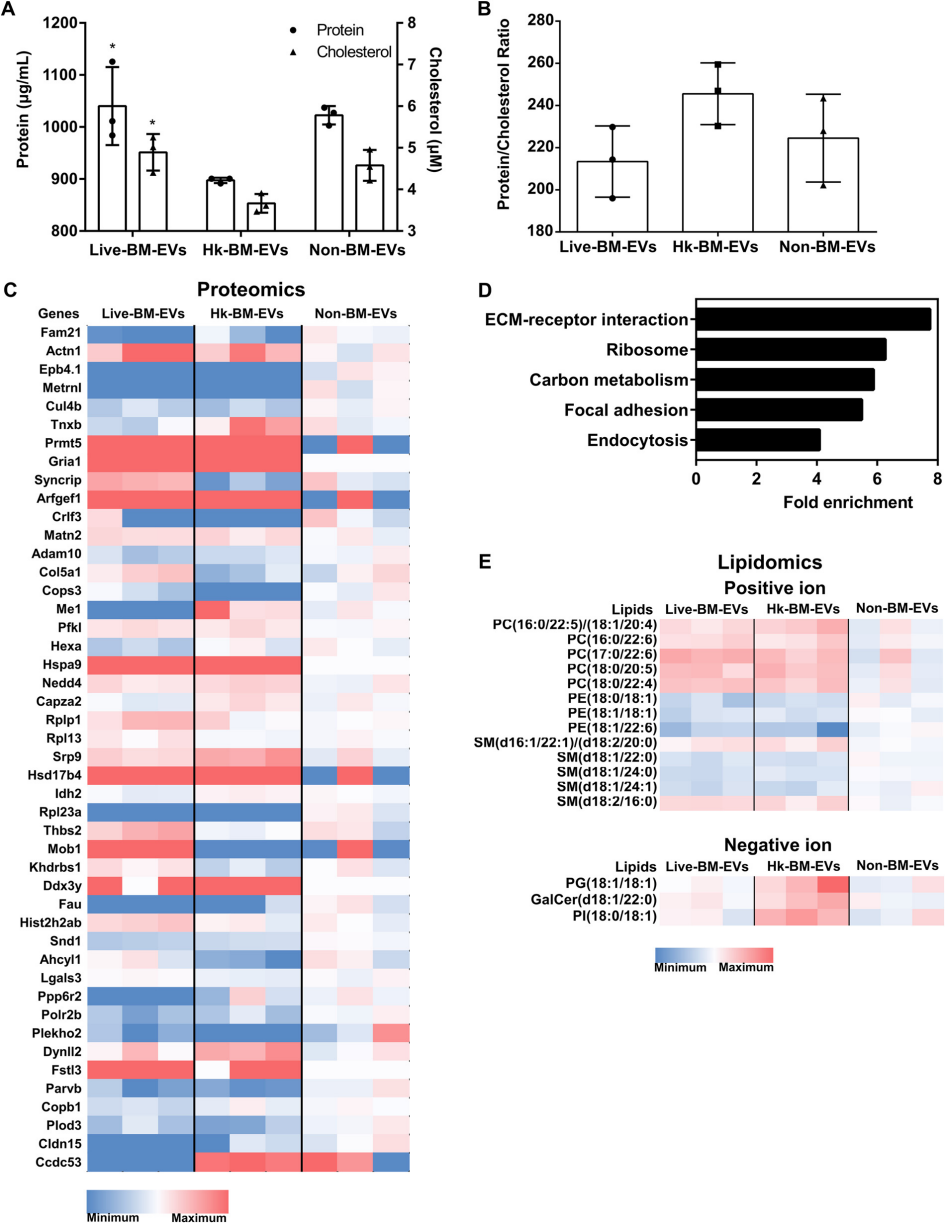
Multi-omics analysis of BMDM-EVs under different cryptococcal stimulations. (A) Protein and cholesterol levels of three types of EV samples. Lower protein and lipid levels are shown in Hk-BM-EVs (EVs from heat-killed C. neoformans-infected activated BMDMs). *, *P* < 0.05 by ANOVA. (B) Ratios of protein to cholesterol in three types of EV samples. (C) Differentially expressed proteins in EVs (*n* = 3 for each type of EV). (D) Pathway analysis of differentially expressed proteins in EVs. (E) Differentially expressed lipids in EVs (*n* = 3 for each type of EV). Live-BM-EVs, EVs from live C. neoformans-infected activated BMDMs; Non-BM-EVs, EVs from activated BMDMs without C. neoformans infection.

Decreased protein and cholesterol concentrations were observed in Hk-BM-EVs, with comparable protein-to-cholesterol ratios in all groups (Fig. 6A and B). We identified 1,481 proteins, 226 lipids, and 59 metabolites in three types of EV samples by a multi-omics approach (19). Proteomic analysis revealed that 46 proteins were differentially expressed among these EVs and that “extracellular matrix (ECM) receptors” was the most enriched pathway (Fig. 6C and D). With regard to lipidomics (Fig. 6E), phosphatidylcholine (PC), phosphatidylethanolamine (PE), and sphingomyelin (SM) were the most differentially expressed lipids. For the metabolomic analysis, no significant difference was detected (Table S3).

10.1128/mBio.00279-21.7 TABLE S3 Metabolomic analysis of EVs by MPLEx. Live-BM-EVs, EVs from live C. neoformans-infected activated BMDMs; Hk-BM-EVs, EVs from heat-killed C. neoformans-infected activated BMDMs; Non-BM-EVs, EVs from activated BMDMs without C. neoformans infection. Download Table S3, DOCX file, 0.03 MB. Copyright © 2021 Zhang et al. 2021 Zhang et al. https://creativecommons.org/licenses/by/4.0/ This content is distributed under the terms of the Creative Commons Attribution 4.0 International license.

Given that all three types of EV samples showed similar biological effects *in vitro*, we further identified “core EV components” as those widely present in all three types of EV samples. A total of 1,151 proteins were detected in all types of EV samples, with no statistical difference, putatively “core EV proteins” of murine macrophages (Table S4). Among them, Fn1, Hspg2, Emilin2, Hist1h4a, and Actb were the top five most enriched proteins.

10.1128/mBio.00279-21.8 TABLE S4 Core proteins widely present in all EVs from macrophages. Live-BM-EVs, EVs from live C. neoformans-infected activated BMDMs; Hk-BM-EVs, EVs from heat-killed C. neoformans-infected activated BMDMs; Non-BM-EVs, EVs from activated BMDMs without C. neoformans infection. Download Table S4, DOCX file, 0.3 MB. Copyright © 2021 Zhang et al. 2021 Zhang et al. https://creativecommons.org/licenses/by/4.0/ This content is distributed under the terms of the Creative Commons Attribution 4.0 International license.

For lipids, each type of EV sample had a set of 210 commonly detected lipids as “core EV lipids” of murine macrophages (Table S5). Among these lipids, SM(d18:1/16:0)_A, PC(16:0/18:1), PC(16:0/20:1)/PC(18:0/18:1), SM(d18:2/24:0), and PC(16:0/16:0)_A were the top five most enriched lipids. We propose that these proteins and lipids are core components of macrophage EVs, widely present in all EVs from murine macrophages and potentially in EVs of other murine cells. Their roles in EV formation, excretion, and function remain to be defined.

10.1128/mBio.00279-21.9 TABLE S5 Core lipids widely present in all EVs from macrophages. Live-BM-EVs, EVs from live C. neoformans-infected activated BMDMs; Hk-BM-EVs, EVs from heat-killed C. neoformans-infected activated BMDMs; Non-BM-EVs, EVs from activated BMDMs without C. neoformans infection. Download Table S5, DOCX file, 0.07 MB. Copyright © 2021 Zhang et al. 2021 Zhang et al. https://creativecommons.org/licenses/by/4.0/ This content is distributed under the terms of the Creative Commons Attribution 4.0 International license.

### Shared proteins and lipids were identified in *Cryptococcus*-infected BM-EVs.

**FIG 7:**
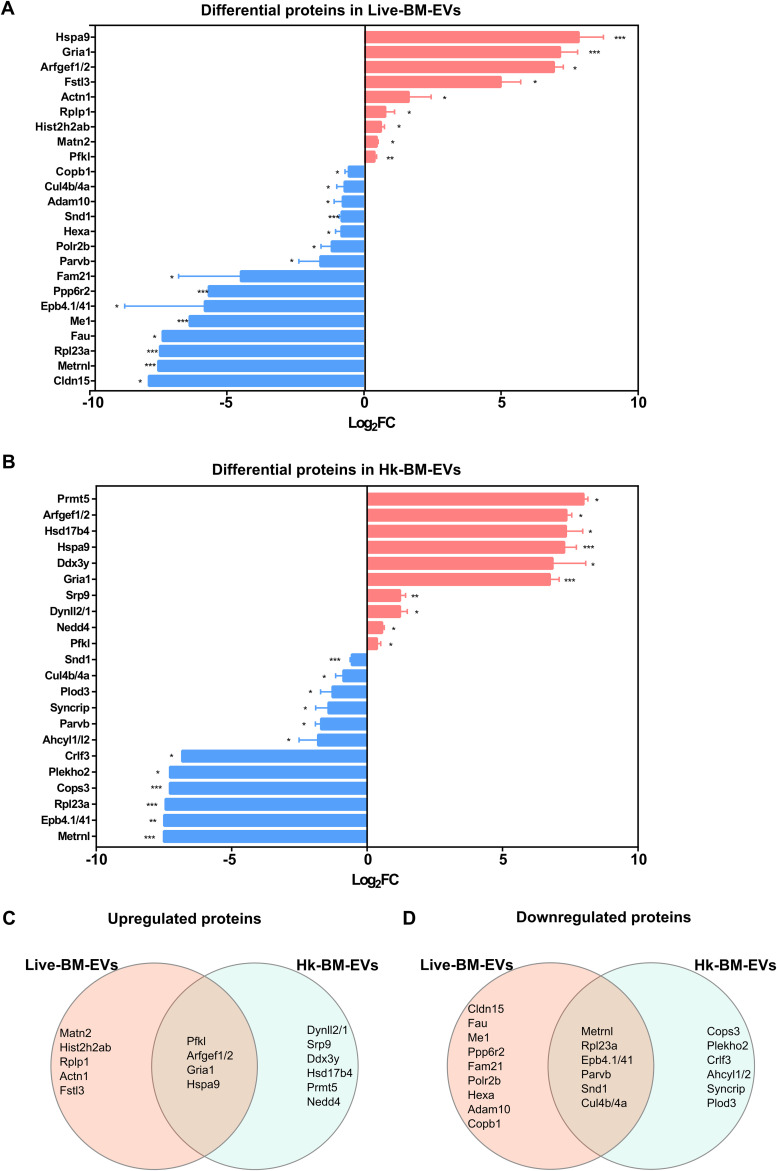
Differential protein composition in EVs from BMDMs after infection with C. neoformans. (A and B) Differential protein expression in live-BM-EVs (EVs from live *Cryptococcus*-infected BMDMs) (A) and Hk-BM-EVs (EVs from heat-killed *Cryptococcus*-infected BMDMs) (B), compared with non-BM-EVs (*n* = 3) (statistical analysis by ANOVA followed by Dunnett’s multiple-comparison test). Log_2_FC, log_2_ fold change. (C and D) Venn analysis of upregulated (C) and downregulated (D) proteins in live-BM-EVs and Hk-BM-EVs. *, *P* < 0.05; **, *P* < 0.01; ***, *P* < 0.001.

Finally, we aimed at identifying EV components associated with *Cryptococcus* infection. Therefore, proteins and lipids in live-BM-EVs and Hk-BM-EVs were analyzed. Compared with proteins in non-BM-EVs, 9 upregulated and 15 downregulated proteins were identified in live-BM-EVs (Fig. 7A); 10 upregulated and 12 downregulated proteins were determined in Hk-BM-EVs (Fig. 7B). Venn analysis identified four upregulated proteins (encoded by genes *Hspa9*, *Gria1*, *Arfgef1*/*2*, and *Pfkl*) and six downregulated proteins (encoded by genes *Metrnl*, *Rpl23a*, *Epb4.1*/*41*, *Parvb*, *Snd1*, and *Cul4b*/*4a*) in both live-BM-EVs and Hk-BM-EVs (Fig. 7C and D).

**FIG 8:**
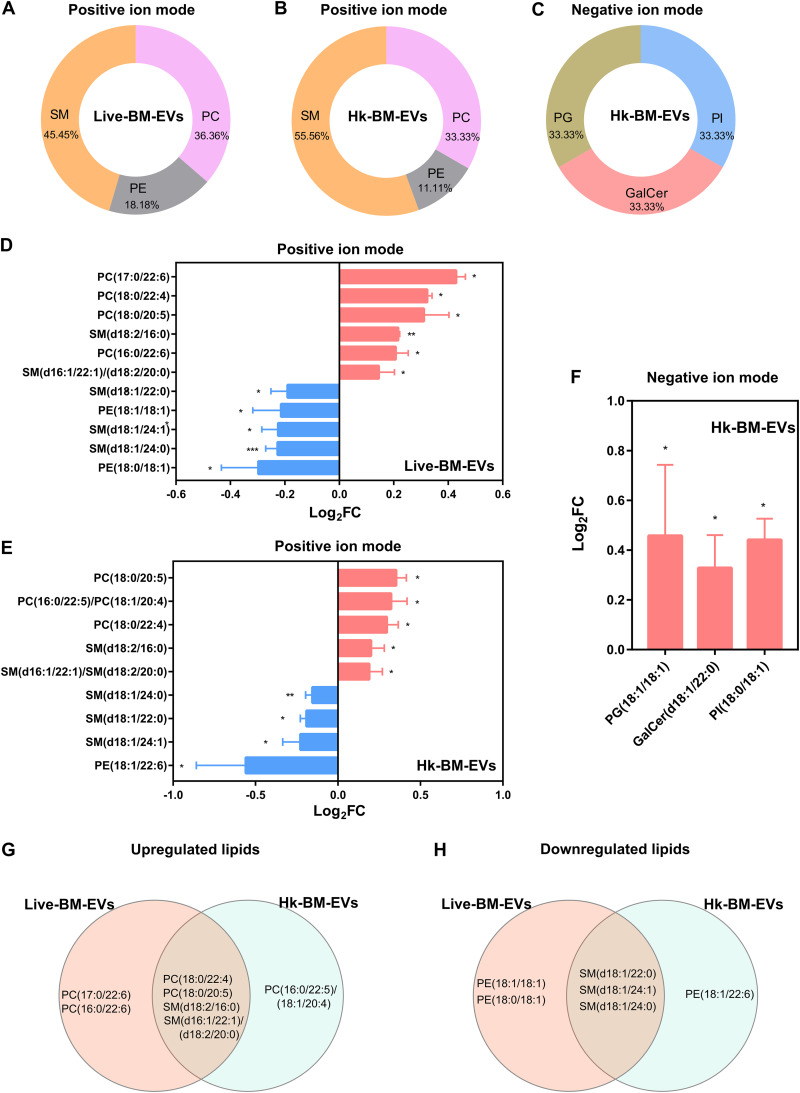
Differential lipid composition in EVs from BMDMs under different cryptococcal stimulations. (A to C) Species percentages of lipids determined by positive and negative ionization modes in live-BM-EVs (EVs from live *Cryptococcus*-infected BMDMs) (A) and Hk-BM-EVs (EVs from heat-killed *Cryptococcus*-infected BMDMs) (B and C) (*n* = 3). (D to F) Fold changes of dysregulated lipids determined by positive and negative ionization modes in live-BM-EVs (D) and Hk-BM-EVs (E and F) (statistical analysis by ANOVA followed by Dunnett’s multiple-comparison test). (G and H) Venn analysis of upregulated (G) and downregulated (H) lipids in live-BM-EVs and Hk-BM-EVs. *, *P* < 0.05; **, *P* < 0.01; ***, *P* < 0.001.

With regard to lipids, compared with non-BM-EVs, SM was the most enriched species in both live-BM-EVs and Hk-BM-EVs using the positive-ion mode (Fig. 8A and B). phosphatidylglycerol (PG), phosphatidylinositol (PI), and galactosylceramides (GalCer) were statistically different in Hk-BM-EVs using the negative mode (Fig. 8C). The upregulated and downregulated lipid profiles in live-BM-EVs and Hk-BM-EVs are shown in Fig. 8D to F. Venn analysis identified four shared upregulated lipids [PC(18:0/22:4), PC(18:0/20:5), SM(d18:2/16:0), and SM(d16:1/22:1)/(d18:2/20:0)] and three downregulated lipids [SM(d18:1/22:0), SM(d18:1/24:1), and SM(d18:1/24:0)] in both live-BM-EVs and Hk-BM-EVs (Fig. 8G and H).

**FIG 9:**
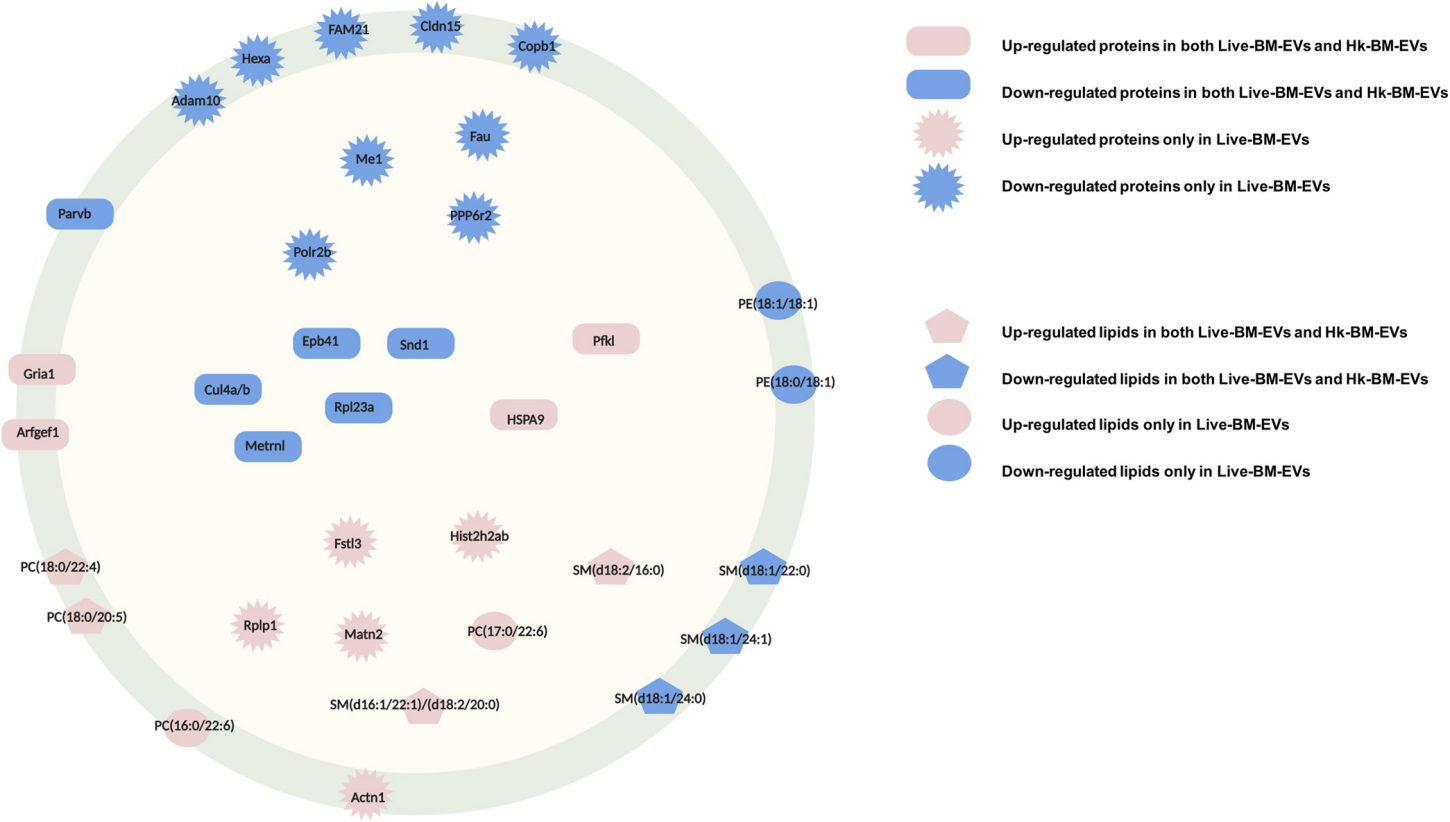
Simplified model of components in EVs from cryptococcus-infected macrophages. Red shapes indicate upregulated proteins or lipids. Blue shapes indicate downregulated proteins or lipids. Components in non-BM-EVs were used as controls. Rectangles indicate shared proteins in infection BM-EVs. Asterisks indicate proteins differentially regulated in live-BM-EVs. Pentagons indicate shared lipids. Circles indicate lipids identified only in live-BM-EVs. Both membrane-associated and intracellular components were found in EVs from cryptococcus-infected macrophages.

Annotated to known subcellular locations, the components (proteins and lipids) of macrophage EVs associated with *Cryptococcus* are simplified in Fig. 9.

## DISCUSSION

Cell-to-cell communication is vital in physiological processes, including immunity and defense against pathogens. EVs are proposed to be key players in intercellular communication as EVs contain protein, lipids, and RNAs (20). On the microbial side, EVs from pathogens carry abundant virulence factors (21). C. neoformans secreted vesicles were proposed to function as “virulence bags” because they contain numerous components associated with virulence, such as glucuronoxylomannan (GXM), laccase, and urease (11, 22). However, it is unknown whether host-derived vesicles are involved in the host response to cryptococcosis.

Here, we demonstrate a proinflammatory role of EVs from BMDMs *in vivo*. Treatment with EVs led to a lower fungal burden in mice. Surprisingly, overall host survival was decreased in the group treated with EVs, which we postulate is due to an overwhelming proinflammatory response in the lungs of EV-treated mice. This hypothesis is supported by the histopathological analysis and *in vitro* analysis of classical biological functions of macrophages. All three types of EV samples increased the fungicidal activities of naive BMDMs, with live-BM-EVs showing the highest ability. Given that nonlytic exocytosis requires viable intracellular fungi (23), the reduction in this fungal cell exit likely reflects the increased fungicidal activity of EV-treated macrophages.

Host damage in cryptococcosis comes from both direct fungally mediated toxicity as well as immune-derived damage, and the reduced survival could reflect enhanced host damage from an EV-stimulated immune response (24). A similar phenomenon was observed with C. neoformans infection in inducible nitric oxide (iNOS)-deficient mice, which manifested reduced survival despite controlling the infection, as the result of an overexuberant inflammatory response (25). Evidence from other intracellular microbes, including Mycobacterium tuberculosis, Salmonella enterica serovar Typhimurium, and Toxoplasma gondii, has also shown a common pattern of host-derived EVs enhancing the host inflammatory response (12, 26).

Analysis of the physical properties of EVs revealed that the main size distribution of these EVs was around 40 to 60 nm, which suggests that these macrophages produced exosomes (commonly 30 to 100 nm in diameter), the smaller type of EVs, rather than microvesicles, which are larger membrane structures (27). Based on our data, DLS tends to underestimate the size of EVs, so TEM is most probably more accurate than DLS. Therefore, the main distribution of macrophage EVs was likely to be around 60 nm. The similarity of the size distributions in these EVs, irrespective of whether the macrophage ingested live or dead fungal cells, implies that the assembly of EVs was mainly determined by the originating cells rather than the viability of the interacting pathogen. Moreover, lower protein and lipid levels were observed in Hk-BM-EVs, which indicates that heat-killed C. neoformans-infected macrophages produced fewer EVs. However, this should be interpreted with caution as all the measurements were performed using EVs secreted from activated macrophages, and it is unknown whether the production of EVs from activated macrophages is dysregulated relative to naive macrophages.

Our *in vitro* data suggest that activated macrophages release EVs that induce naive macrophages to adopt a proinflammatory phenotype, with live-BM-EVs showing stronger effects. The higher proinflammatory feature of live-BM-EVs may be caused by the inflammatory synergy between exposure to viable *Cryptococcus* and IFN-γ in macrophages (6). The multi-omics analysis also identified components that were dysregulated only in live-BM-EVs, including Fstl3, which can increase the proinflammatory response of macrophages (28). On the other hand, EVs from infected host cells could also participate in immune response modulation via the transfer of antigens (Ags) to recipient cells or signaling such as Toll-like receptor-related pathways (29). Consistent with this, live-BM-EVs induced a higher enrichment of GOs related to immune responses in naive macrophages, such as “defense response to virus” and “innate immune response.” Following the mouse observations, immune-related p53 signaling, widely reported in the macrophage response against C. neoformans (30), was observed as a shared target of EVs in both naive human and murine macrophages.

Furthermore, in EVs associated with C. neoformans (live-BM-EVs and Hk-BM-EVs), higher levels of alarmin proteins, GRIA1, ARFGEF1/2, and HSPA9, were identified as shared components. These proteins are immunity modulators via pathways such as STAT5 (31). Notably, GRIA1, a regulator in glutamatergic signaling (32), and HSPA9, a stress-associated protein (33), were detected only in EVs from infected macrophages, suggesting specific roles in intercellular communication during infection. The presence of alarmins, including heat shock proteins (HSPs), in macrophage EVs was also reported previously by Pieters et al. (34). Different from the proteomics from human macrophage EVs associated with infection (35), cytokines were not identified in our murine infected macrophages.

With regard to lipids associated with cryptococcal infection, PC species replaced with PC(16:0/22:5)/PC(18:1/20:4), PC(16:0/22:6), PC(17:0/22:6), PC(18:0/20:5), and PC(18:0/22:4) were induced in infected BM-EVs. These long polyunsaturated fatty acids are precursors of important mediators of inflammation, such as eicosanoids, and prostaglandins (36). For the SM species, SM(d18:1/24:0), decreased in the infection BM-EVs, was reported to activate macrophages directly (37). In light of these observations, we propose that activated macrophage EVs during cryptococcal infection may function by modulating immune responses in recipient cells.

It is worth noting that all macrophage-derived vesicles triggered similar protective roles *in vitro*. First, all the vesicles were produced by LPS- and IFN-γ-activated macrophages, which may directly influence EV composition. The direct role of LPS in modulating macrophage antimicrobial vesicles was previously reported by Ding et al. (38). Additionally, the proinflammatory effects of EVs may also be caused by host components. Among the top core EV proteins in all these EVs, the most enriched protein, Fn1, also known as an alarmin in macrophage EVs, was associated with inflammatory disease (34, 39), followed by Hspg2, which was reported to regulate an NF-κB-mediated pathway (40). SM(d18:1/16:0), the most enriched core EV lipid, increased cell ATP production through enhanced aerobic glycolysis (41). Given the similar biological effects of EVs, our results suggest the need for more detailed characterization of EVs, including future studies to decipher molecular-level effectors carried in EVs and perhaps manipulate the EV composition to achieve beneficial effects in the host.

In conclusion, this study demonstrated that EVs from activated macrophages mediated a proinflammatory signal both *in vivo* and *in vitro*, suggesting a potential self-regulatory loop during the host antifungal response. This study provides a new window for understanding the interplay between the host and pathogen and indicates new targets for immune-related therapy in the future.

## MATERIALS AND METHODS

### C. neoformans strain.

Strain H99 (serotype A) was grown in yeast extract-peptone-dextrose (YPD) at 30°C with moderate shaking (150 rpm) for 48 h to reach the stationary phase. Heat-killed (Hk) C. neoformans cells prepared by immersion in a water bath at 65°C for 30 min. C. neoformans cells were centrifuged, counted, and resuspended in Dulbecco’s modified Eagle’s medium (DMEM; Gibco).

### Macrophage infection.

BMDMs were harvested as previously reported (42). In brief, hind leg bones of 6- to 8-week-old C57BL/6J female mice (National Cancer Institute) were used as a source of BMDMs, which were then matured at 37°C in 10% CO_2_ for 6 to 8 days in DMEM with 20% L-929 cell-conditioned medium, 10% fetal bovine serum (FBS), 2 mM l-glutamine, 1% nonessential amino acids, 1% HEPES buffer, and β-mercaptoethanol. Macrophages were activated with gamma interferon (0.2 ng/ml) and lipopolysaccharide (0.5 μg/ml) for 18 h before C. neoformans infection and then incubated with either live or heat-killed H99 cells (1 yeast cell per host cell) for 1 h or without any C. neoformans. Free C. neoformans cells were removed by two gentle washes with PBS. These infected BMDMs were then incubated with FBS-depleted medium at 37°C in 10% CO_2_ for 6 h for EV harvest. MDMs and M1 phenotype MDMs were cultured according to routine protocols (43). In brief, human peripheral monocytes were harvested from buffy coats of healthy donors by using CD14^+^ magnetic microbeads. Next, these monocytes were cultured in untreated plates in RPMI 1640 containing 10% FBS plus 1% penicillin-streptomycin (pen-strep) with 25 ng/ml granulocyte-macrophage colony-stimulating factor (GM-CSF) for 5 days. Fresh medium with GM-CSF was changed on day 3. After 5 days of differentiation, LPS (500 ng/ml) and IFN-γ (10 ng/ml) were added 2 days before infection. The harvesting of EVs from MDMs was based on the same protocol as the one described above for BMDMs. In this study, activated macrophages were used to isolate EVs. The usage of human blood samples was approved by Shanghai Changzheng Hospital under project NSFC 31770161. All animal experiments were approved by the Johns Hopkins University IACUC under protocol MO18H152.

### EV isolation.

EVs were isolated according to a previously described method, with some modifications (11). In brief, the supernatant of the culture suspension from each group was collected, followed by sequential centrifugations at 450 × *g* for 10 min at 4°C followed by 3,000 × *g* for 10 min at 4°C. The supernatant passed through an 0.8-μm filter and concentrated using a 100-kDa membrane. The concentrate was then ultracentrifuged at 100,000 × *g* for 2 h at 4°C. Pellets were washed with sterile PBS, followed by centrifugation at 12,000 × *g* for 5 min, and the supernatant was then ultracentrifuged again at 100,000 × *g* for 60 min. Pellets were resuspended in sterile PBS for subsequent experiments. In this study, we extracted vesicles from cell culture media under three conditions: macrophages incubated with live *Cryptococcus* cells, macrophages incubated with heat-killed *Cryptococcus* cells, and macrophages only.

### EV quantification.

A Micro BCA protein assay kit (Thermo Fisher) and an Amplex red cholesterol assay kit (Thermo Fisher) were used to detect whole protein and cholesterol, respectively. All experiments were performed according to the manuals.

### Dynamic light scattering.

EV sizes (z-averaged diameter) were measured by dynamic light scattering (DLS) using Zetasizer (Nano ZS; Malvern). EVs were obtained by ultracentrifugation and diluted in PBS, samples were then transferred to a disposable cuvette, and 10 measurements for each sample were performed with a refractive index of 1.33 and absorption at 0.01. Data analysis was performed using Zetasizer 7.11 software (Malvern).

### Coculture of macrophages with EVs.

The amount of EVs added to macrophages was based on a protocol described previously by Oliveira et al. (12). In brief, 1 × 10^6^ naive macrophages were seeded in a 6-well plate, and an amount of EVs corresponding to 400 ng cholesterol was added to activate macrophages. Cells were incubated at 37°C in 10% CO_2_ for 24 h. For experiments with C. neoformans challenge, live C. neoformans cells at a ratio of 1:1 were added to macrophages after a 24-h incubation with EVs, unless otherwise described.

### Nitrite measurement.

A Griess assay was used to detect the concentration of nitrite in the supernatant, as previously described, with minor modifications (44). In brief, the culture supernatant in 100 μl was collected, and an equal volume of Griess reagent (1:1 ratio of 0.1% naphthyl ethylenediamine dihydrochloride and 1% sulfanilamide in 5% H_3_PO_4_) was added. The mixture was incubated in the dark for 10 min at room temperature. The absorbance of the mixture was measured at 562 nm using the EMax Plus microplate reader (Molecular Devices). The nitrite concentration was determined from a standard curve constructed with 0 μM to 50 μM sodium nitrite.

### Real-time PCR.

Total RNA was extracted using TRIzol (Ambion). The quantity and quality of RNA were validated by using a Nanodrop 2000 system (Thermo Fisher). cDNA was synthesized using a high-capacity cDNA reverse transcription (RT) kit (Applied Biosystems). Real-time PCR was performed using SYBR green according to the manufacturer’s instructions. The primers were designed and subjected to NCBI BLAST analysis (https://blast.ncbi.nlm.nih.gov/). The sequences are as follows: forward primer CAGCAACTCCCACTCTTCCAC (5′ to 3′) and reverse primer GGTCCAGGGTTTCTTACTC (5′ to 3′) for glyceraldehyde-3-phosphate dehydrogenase (GAPDH), forward primer TGACATCAACACTCCCCTGACAAC (5′ to 3′) and reverse primer CCTTTTCTTCCTTCCCAGCAG (5′ to 3′) for *Arg1*, and forward primer CCTGCTGTTCACAGTTGCC (5′ to 3′) and reverse primer ATTGGGATCATCTTGCTGGT (5′ to 3′) for *Ccl2*.

### Phagocytosis.

Phagocytosis was performed according to the protocol described previously by Nicola and Casadevall, with some minor modifications (45). In brief, 5 × 10^4^ macrophages were seeded on 3-ml plates. After incubation with EVs for 24 h, 1.5 × 10^5^ live C. neoformans cells with 20% complement (as opsonin) were added and incubated for 2 h. Ice-cold methanol was used to fix the cells, and cells were washed twice with PBS. Giemsa stain was added to each plate, and the mixture was incubated at 4°C overnight. An inverted microscope was used to observe phagocytosis. For phagocytosis percentages, the number of macrophages internalizing fungal cells was divided by the total number of macrophages. At least 100 macrophages were counted for each group.

### Time-lapse microscopy.

Approximately 3 × 10^4^ cells were seeded into a 3-ml plate with a coverslip at the bottom. After being treated with EVs for 24 h (24 ng EVs), 1.5 × 10^5^ live C. neoformans cells with 10 μg/ml 18B7 antibody (used as opsonin) were added. Cells were incubated at 37°C in 10% CO_2_ for 2 h to allow phagocytosis to proceed. After 5 washes with medium to remove extracellular C. neoformans, 2 ml medium without 10 μg/ml 18B7 was added. Time-lapse images were taken every 4 min for a period of 12 h at 37°C with 10% CO_2_ at a ×10 magnification on an Axiovert 200M microscope (Zeiss). Time-lapse movies were analyzed using AxioVision software (Zeiss), and the number of cells exhibiting nonlytic exocytosis was recorded by operator visual analysis of the movie.

### Transmission electron microscopy.

EVs were adsorbed to glow-discharged (EMS GloQube) carbon-coated 400-mesh copper grids (EMS) by floatation for 2 min. Grids were quickly blotted and then rinsed in 3 drops (1 min each) of Tris-buffered saline (TBS). Grids were negatively stained with 2 consecutive drops of 1% uranyl acetate, blotted, and then quickly aspirated to obtain a thin layer of stain covering the sample. Grids were imaged on a Phillips CM-120 transmission electron microscope operating at 80 kV with an AMT XR80 charge-coupled-device (CCD) camera (8 megapixels).

### Confocal microscopy.

Confocal microscopy was performed as described previously by Gonçalves et al. (46). In brief, BMDMs were plated at 2 × 10^5^ cells/well into 24-well plates, covered with a sterile glass coverslip, and cultured overnight at 37°C in 10% CO_2_. EVs (20 μg protein) were stained with 3 mM DiI (1,1′-dioctadecyl-3,3,3′,3′-tetramethylindocarbocyanine perchlorate [DiIC_18_]; Life Technologies) at 4°C for 30 min. EVs were ultracentrifuged at 100,000 × *g* for 1 h and then resuspended in 3 ml of 0.22-μm-filtered PBS. EVs were added to BMDMs and incubated for different intervals (30 min, 60 min, 2 h, 4 h, and 8 h). After the incubations, cells were washed and fixed with 4% paraformaldehyde for 40 min at 37°C. Cells were washed and blocked with 1% PBS–bovine serum albumin (BSA) for 1 h. Cells were washed and labeled with cholera toxin subunit B (CtxB) (recombinant)-Alexa Fluor 488 (Sigma-Aldrich) at 1 μg/ml for 1 h at 40°C. After three more washes, cells were labeled with 4′,6-diamidino-2-phenylindole (DAPI) (10 μg/ml) for 30 min at room temperature. The coverslips were washed three times with PBS and mounted in a solution containing 50% glycerol and 50 mM *n*-propyl gallate in PBS. The slides were visualized using a Zeiss LSM 710NLO-Meta confocal microscope with a 40× objective. DAPI-labeled cell nuclei were excited with a two-photon excitation regime using a Mai Tai HPpulsed infrared laser (Spectra-Physics, Lasers) at 740 nm. CtxB-Alexa Fluor 488- and DiIC_18_-labeled EVs were excited with an argon-ion laser at 488 nm and a diode laser at 561 nm, respectively. Emissions were collected in three separated channels using band-pass filters for green (BP 500 to 550 IR), red (BP 575 to 615 IR), and blue (BP 435 to 485 IR) channels. All images were collected using AxioVision 4.8. software (Carl Zeiss). Pinhole diameters were set to 1 Airy unit, corresponding to a z resolution of 0.8 mm. The displayed results are representative of those from three independent experiments.

### Killing assay.

Approximately 3 × 10^4^ macrophages were plated in 96-well plates. After incubation with EVs (24 ng) for 24 h, live C. neoformans cells were added at a ratio of 1:1 with either 10 μg/ml 18B7 or 20% guinea pig complement as an opsonin, or C. neoformans cells were added alone. After 2 h of phagocytosis, the supernatant was removed, followed by five gentle washes with PBS to remove extracellular C. neoformans. Sterile water was added and pipetted up and down several times to lyse macrophage cells. The suspension was then 100-fold diluted, 50 μl was added to YPD plates (in triplicates), and the plates were incubated at 30°C for 2 days before counting individual colonies.

### Multi-omics analysis.

EVs were placed in a 50-ml conical tube, and liquids were removed. Next, EVs were dried by placing the tube upside down for several hours and stored at −80°C. Samples were sent to the Pacific Northwest National Laboratory (PNNL) for multi-omics (proteomic, metabolomic, and lipidomic) detection (19). For simultaneous metabolite, protein, and lipid extraction (MPLEx), 5 volumes of −20°C chloroform-methanol (2:1) were added and incubated on ice for 5 min, spun for 1 min, and centrifuged at 12,000 rpm for 10 min at 4°C. The phases containing metabolites and lipids at the top and bottom, respectively, were collected in an autosampler tube and dried in a vacuum centrifuge. The protein particles were washed by adding 1 ml of −20°C methanol and centrifuged at 12,000 rpm for 10 min at 4°C. The supernatant was discarded, and the precipitated protein was dried in a vacuum centrifuge. Hydrophilic metabolites were derivatized with methoxamine and *N*-methyl-*N*-(trimethylsilyl)trifluoroacetamide (MSTFA) and analyzed on a GC 7890A gas chromatography-mass spectrometry (GC-MS) system (Agilent Technologies) as previously described (47). Data were analyzed with MetaboliteDetector (48), and metabolites were identified by matching against the PNNL augmented version of the Fiehn Library (49). Lipids were dissolved in methanol and analyzed by reverse-phase chromatography (Waters CSH column, 3.0 mm by 150 mm, 1.7-μm particle size) in a NanoAcquity ultraperformance liquid chromatography (UPLC) system (Waters) interfaced with a Velos Orbitrap mass spectrometer (Thermo Fisher) as described previously (50). Lipid species were identified with LIQUID (50) and manually inspected before having the features (peak areas) extracted with MZmine 2.0 (51). Proteins were digested with trypsin (19) and analyzed by liquid chromatography-tandem mass spectrometry (LC-MS/MS) in a Q-Exactive mass spectrometer (Thermo Fisher) as described previously by Coelho et al. (21). LC-MS/MS data were analyzed using MaxQuant (version 1.5.5.1) (52) by searching against the C. neoformans H99 and mouse reference proteome databases from the UniProt Knowledge Base (both downloaded on 14 August 2018). Only peptides with trypsin cleavage in both termini were considered, with up to 2 missed cleavage sites. Cysteine carbamidomethylation was considered an invariable modification, while protein N-terminal acetylation and methionine oxidation were considered variable modifications. Label-free quantification (LFQ) was used for quantitative analysis. Metabolomics, lipidomics, and proteomics data were normalized by linear regression and central tendency, followed by analysis of variance (ANOVA) using Inferno RDN (formerly Dante) (53).

### Transcriptome analysis.

Genespring (version 14.8; Agilent Technologies) was used to process the raw data. In brief, the raw data were normalized with the quantile algorithm. For subsequent comparisons, at least one set of samples must have a positive "detected" signal. Differentially expressed genes were then identified through fold changes as well as *P* values calculated with a *t* test. The cutoff set for up- and downregulated genes was a fold change of ≥1.5 and a *P* value of ≤0.05. Next, GO analysis and KEGG analysis were performed to determine the regulatory roles of these differentially expressed mRNAs.

### *In vivo* infection.

Animal studies were performed using 6- to 8-week-old female C57BL/6 mice. Each mouse was injected i.p. with 150 μg protein (resuspended in 150 μl PBS) 1 day before H99 challenge. H99 cells were grown for 18 h at 37°C with shaking at 180 rpm in YPD broth, followed by a wash with PBS, and resuspended to 2.5 × 10^7^ cells/ml PBS. Mice were then anesthetized with 2% isoflurane anesthesia, followed by intranasal infection with a 20-μl H99 suspension. At 2 weeks postinjection, mice were euthanized, and lung and brain were removed and homogenized by passing through a 100-μm filter. Homogenates were diluted and plated onto YPD agar for CFU counting. Survival analysis was also performed.

### Statistical analysis.

Data are reported as the means from at least three independent experiments. Continuous variables were displayed as the means ± standard deviations (SD) and compared by Student’s *t* test. The survival difference was determined using the log rank test, and *P* values of <0.05 were considered statistically significant. All statistical analyses were performed with GraphPad Prism software (GraphPad, La Jolla, CA, USA). Venn analysis was performed based on InteractiVenn (54).
