# Supplementary material for: Cryptococcus neoformans-Infected Macrophages Release Proinflammatory Extracellular Vesicles: Insight into Their Components by Multi-omics
Source: mBio. 2021 Mar 30;12(2):e00279-21. doi: 10.1128/mBio.00279-21 (PMC8092229; doi:10.1128/mBio.00279-21)
Supplement: FIG S1 [file mBio.00279-21-sf001.docx]

Figure S1. The incorporation of EVs by naïve macrophages.

Fig. S1

**
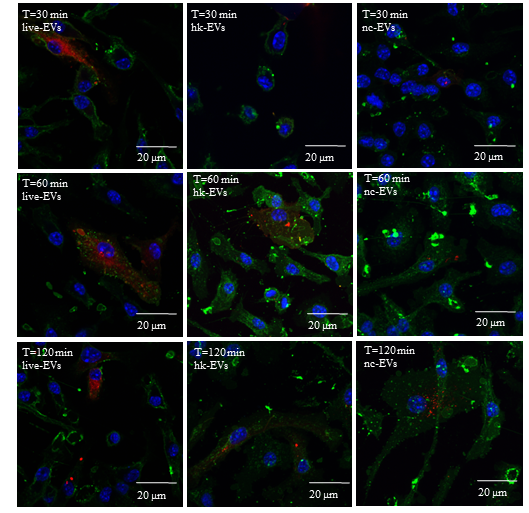
**

**Fig. S1.** The incorporation of three types of activated BMDM-EVs samples by naive BMDMs analyzed by confocal microscope. Red fluorescence represents vesicles with DiI staining; Green fluorescence represents cell membrane with CtxBAlexa staining; Blue fluorescence represents nucleus with DAPI staining. Live-EVs: inactivated BMDMs incubated with EVs from live *C. neoformans* infected activated BMDM; hk-EVs: inactivated BMDM with EVs from hk *C. neoformans* infected activated BMDM; nc-EVs: inactivated BMDM with EVs from non-infected activated BMDM. Hk: heat-killed.
