# Supplementary material for: Cryptococcus neoformans-Infected Macrophages Release Proinflammatory Extracellular Vesicles: Insight into Their Components by Multi-omics
Source: mBio. 2021 Mar 30;12(2):e00279-21. doi: 10.1128/mBio.00279-21 (PMC8092229; doi:10.1128/mBio.00279-21)
Supplement: TABLE S1 [file mBio.00279-21-st001.docx]

Table S1. The nitrite concentrations of naive BMDMs after incubated with three types of EVs samples.

**Table S1. The nitrite concentrations of naive BMDMs after incubated with three types of EVs samples**

| **Group** | **OD raw value** | **OD-blank** |
| --- | --- | --- |
| Live-BM-EVs | 0.085 | -0.06365 |
| Live-BM-EVs | 0.0779 | -0.07075 |
| Live-BM-EVs | 0.07925 | -0.0694 |
| Live-BM-EVs | 0.0762 | -0.07245 |
| Hk-BM-EVs | 0.08165 | -0.067 |
| Hk-BM-EVs | 0.07775 | -0.0709 |
| Hk-BM-EVs | 0.08215 | -0.0665 |
| Hk-BM-EVs | 0.0769 | -0.07175 |
| Non-BM-EVs | 0.07525 | -0.0734 |
| Non-BM-EVs | 0.0769 | -0.07175 |
| Non-BM-EVs | 0.07285 | -0.0758 |
| Non-BM-EVs | 0.07155 | -0.0771 |
| No EVs | 0.076 | -0.07265 |
| No EVs | 0.0844 | -0.06425 |
| No EVs | 0.08215 | -0.0665 |
| Standard (0μM) | 0.14865 | 0 |
| Standard (7.8125μM) | 0.17195 | 0.0233 |
| Standard (15.625μM) | 0.21915 | 0.0705 |
| Standard (31.25μM) | 0.312 | 0.16335 |
| Standard (62.5μM) | 0.45535 | 0.3067 |

Live-BM-EVs: macrophages treated with EVs from live *C. neoformans* infected activated BMDMs; Hk-BM-EVs: macrophages treated with EVs from heat-killed *C. neoformans* infected activated BMDMs;

Non-BM-EVs: macrophages treated with EVs from activated BMDMs without *C. neoformans* infection;

Hk: heat-killed;

No EVs: EVs non-treated macrophages.
