# Supplementary material for: Cryptococcus neoformans-Infected Macrophages Release Proinflammatory Extracellular Vesicles: Insight into Their Components by Multi-omics
Source: mBio. 2021 Mar 30;12(2):e00279-21. doi: 10.1128/mBio.00279-21 (PMC8092229; doi:10.1128/mBio.00279-21)
Supplement: TABLE S2 [file mBio.00279-21-st002.docx]

Table S2. The statistical difference comparison of *in vitro* effects between *C. neoformans* activated BM-EVs and Non-BM-EVs.

**Table S2. The statistical difference comparison of *in vitro* effects between *C. neoformans* activated BM-EVs and Non-BM-EVs**

|  |  | **Live-BM-EVs vs Non-BM-EVs** | **Hk-BM-EVs vs Non-BM-EVs** |
| --- | --- | --- | --- |
| **Phagocytosis** | P value | 0.0855 | 0.9806 |
| **Log_10_ CFU (18B7)** | P value | 0.0043* | 0.032* |
| **Log_10_ CFU (COMPLEMENT)** | P value | 0.0324* | 0.2797 |
| **Log_10_ CFU (non)** | P value | 0.0385* | 0.6224 |
| **Exocytosis** | P value | 0.2875 | 0.075 |
| ***Ccl*2 mRNA** | P value | 0.4976 | 0.2377 |
| ***Arg*1 mRNA** | P value | 0.2383 | 0.8114 |

Live-BM-EVs: macrophages treated with EVs from live *C. neoformans* infected activated BMDMs;

Hk-BM-EVs: macrophages treated with EVs from heat-killed *C. neoformans* infected activated BMDMs;

Non-BM-EVs: macrophages treated with EVs from activated BMDMs without *C. neoformans* infection; No EVs: EVs non-treated macrophages;

Hk: heat-killed;

An unpaired t-test was used to calculate p-values.
