# Supplementary material for: Cryptococcus neoformans-Infected Macrophages Release Proinflammatory Extracellular Vesicles: Insight into Their Components by Multi-omics
Source: mBio. 2021 Mar 30;12(2):e00279-21. doi: 10.1128/mBio.00279-21 (PMC8092229; doi:10.1128/mBio.00279-21)
Supplement: FIG S2 [file mBio.00279-21-sf002.docx]

Figure S2. Transcriptome analysis of naive BMDMs incubated with Hk-BM-EVs and Non-BM-EVs.

**Fig. S2**

**
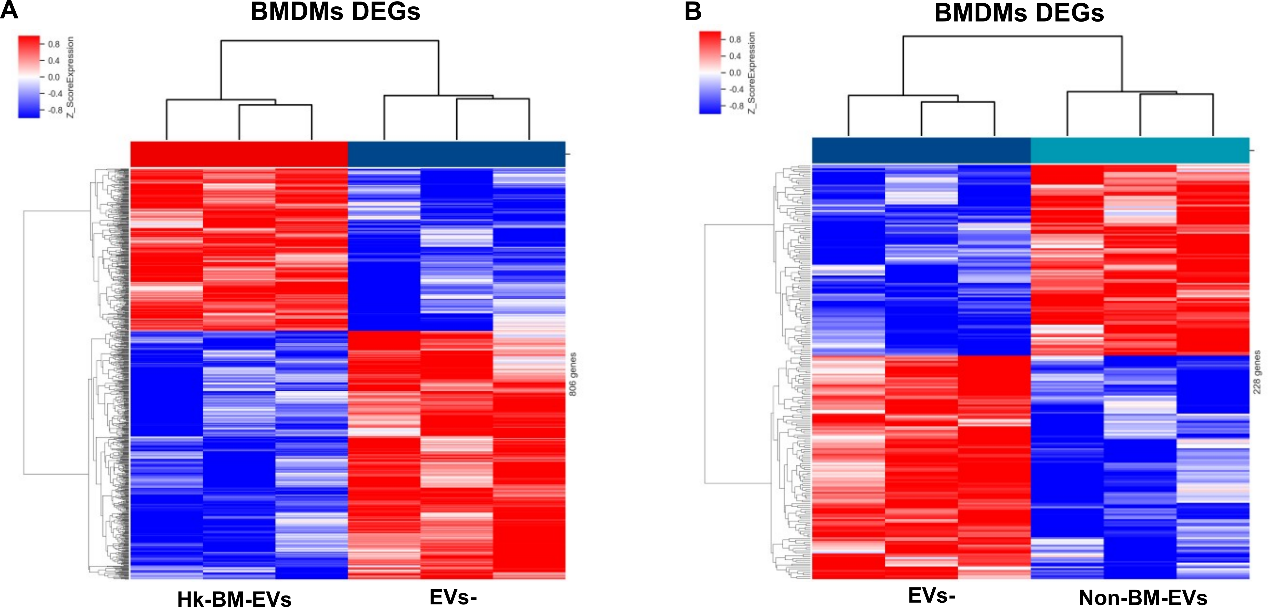
**

**Fig. S2.** Transcriptome analysis of naive BMDMs incubated with Hk-BM-EVs (A) and Non-BM-EVs (B), red for upregulated and blue for downregulated. n=3 for each group. (A) Using fold change of at least 1.5 and p-value < 0.05 as cut-offs, there were 319 upregulated and 487 downregulated genes in naïve BMDMs incubated with Hk-BM-EVs in comparison with BM-EVs non-treated BMDMs. (B) In naïve BMDMs incubated with Non-BM-EVs, there were 105 upregulated and 123 downregulated genes compared to BM-EVs non-treated BMDMs. Hk-BM-EVs: EVs from heat- killed *C. neoformans* infected BMDMs; Non-BM-EVs: EVs from activated BMDMs without infection; EVs-: EVs non treated naïve macrophages; Hk: heat-killed.
