# Supplementary material for: Cryptococcus neoformans-Infected Macrophages Release Proinflammatory Extracellular Vesicles: Insight into Their Components by Multi-omics
Source: mBio. 2021 Mar 30;12(2):e00279-21. doi: 10.1128/mBio.00279-21 (PMC8092229; doi:10.1128/mBio.00279-21)
Supplement: FIG S3 [file mBio.00279-21-sf003.docx]

Figure S3. Transcriptome analysis of naive MDMs incubated with Hk-M-EVs and Non-M-EVs.

**Fig. S3**

**
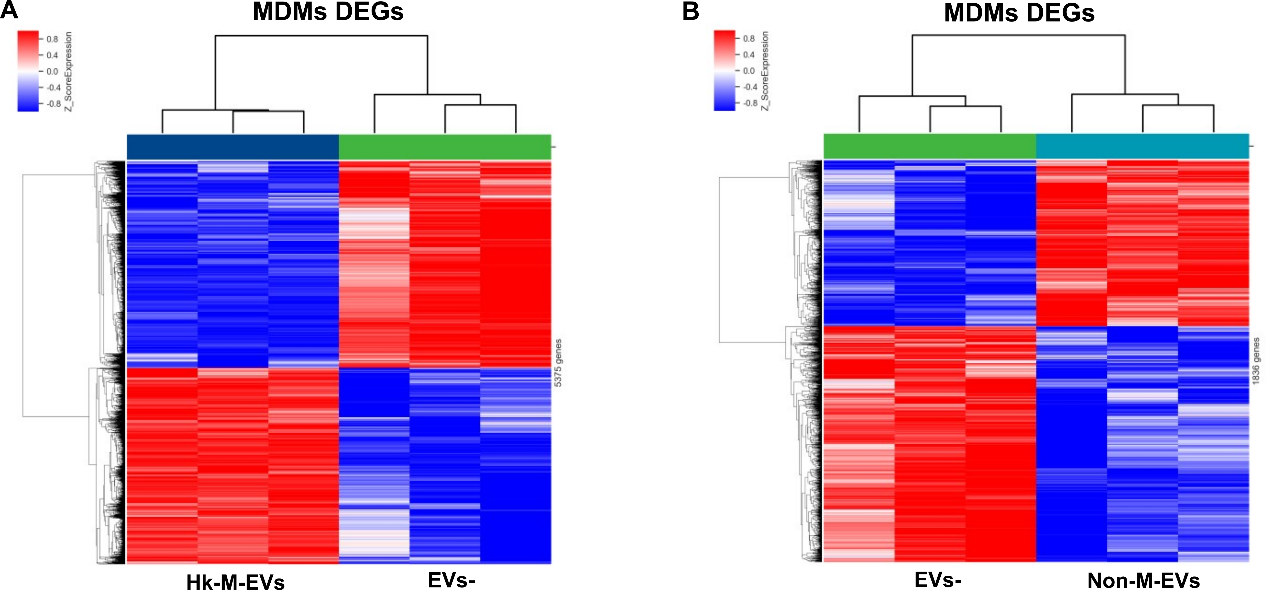
**

**Fig. S3.** Transcriptome analysis of naive MDMs incubated with Hk-M-EVs (A) and Non-M-EVs (B), red for upregulated and blue for downregulated. n=3 for each group. (A) Using fold changes of at least 1.5 and p-value < 0.05 as cut-offs, there were 2617 upregulated and 2758 genes downregulated in naïve MDMs incubated with Hk-M-EVs, compared with EVs non-treated MDMs. (B) In naïve MDMs incubated with Non-M-EVs, there were 759 upregulated and 1077 downregulated genes compared to EVs non-treated MDMs. Hk-M-EVs: EVs from heat-killed *C. neoformans* infected activated human peripheral MDMs; Non-M-EVs: EVs from activated human peripheral MDMs without infection; EVs-: EVs non treated naïve macrophages; Hk: heat-killed.
