# Supplementary material for: Cryptococcus neoformans-Infected Macrophages Release Proinflammatory Extracellular Vesicles: Insight into Their Components by Multi-omics
Source: mBio. 2021 Mar 30;12(2):e00279-21. doi: 10.1128/mBio.00279-21 (PMC8092229; doi:10.1128/mBio.00279-21)
Supplement: FIG S4 [file mBio.00279-21-sf004.docx]

Figure S4. Frequency distributions of naïve BMDMs-EVs under different cryptococcal stimulations were analyzed by DLS and TEM.

**Fig. S4**


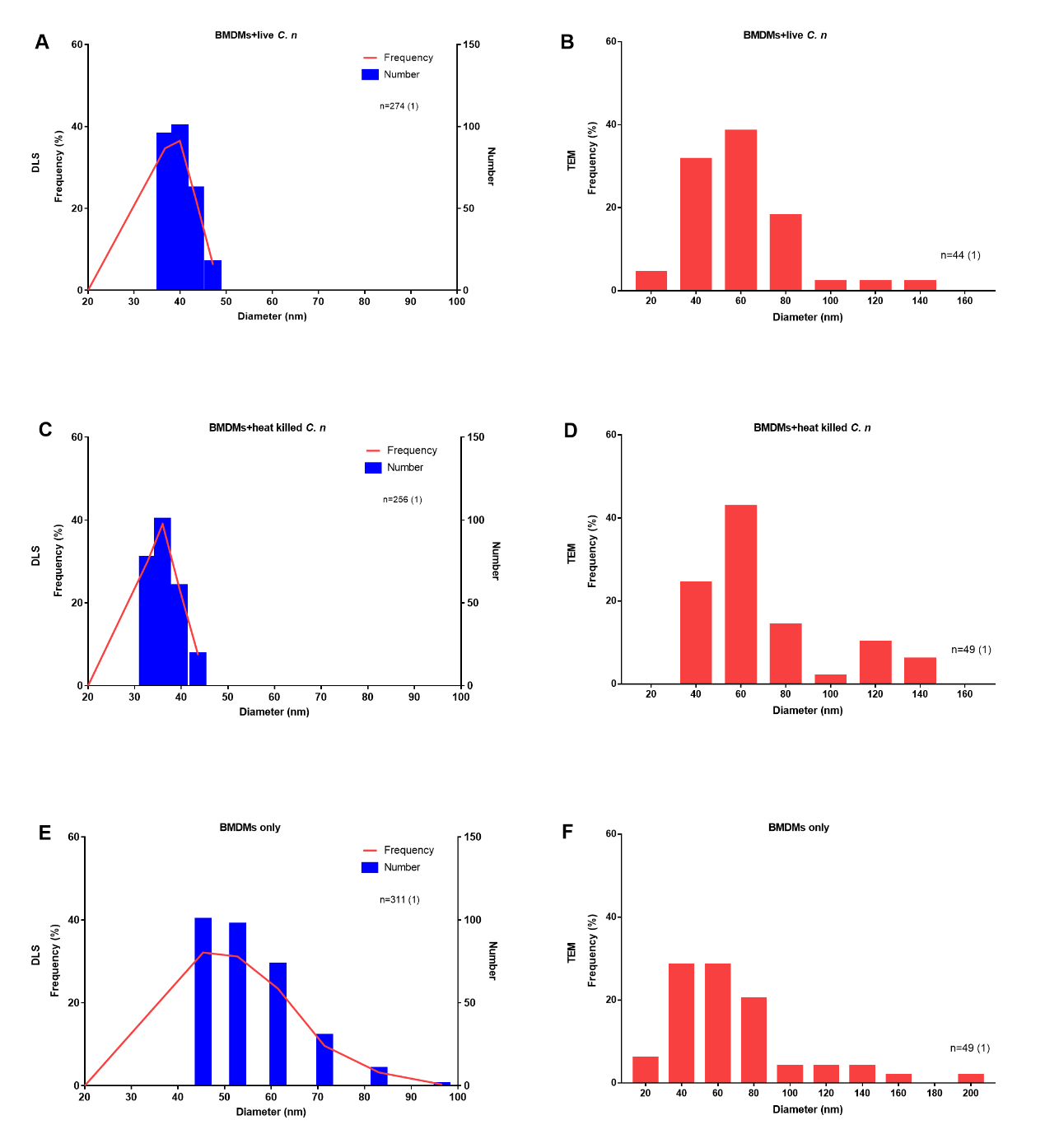


**Fig. S4**. Frequency distributions of EVs from naïve macrophages under different cryptococcal stimulations. **(A,B)** Frequency distributions of Live-BM-EVs analyzed by DLS (A) and TEM (B). **(C,D)** Frequency distributions of Hk-BM-EVs analyzed by DLS (C) and TEM (D). **(E,F)** Frequency distributions of Non-BM-EVs analyzed by DLS (E) and TEM (F). Live-BM-EVs: EVs from live *C. neoformans* infected activated BMDMs; Hk-BM-EVs: EVs from heat-killed *C. neoformans* infected activated BMDMs; Non-BM-EVs: EVs from activated BMDMs without *C. neoformans* infection; Hk: heat-killed.
