# Supplementary material for: Cryptococcus neoformans-Infected Macrophages Release Proinflammatory Extracellular Vesicles: Insight into Their Components by Multi-omics
Source: mBio. 2021 Mar 30;12(2):e00279-21. doi: 10.1128/mBio.00279-21 (PMC8092229; doi:10.1128/mBio.00279-21)
Supplement: TABLE S3 [file mBio.00279-21-st003.docx]

Table S3. The metabolomic analysis of EVs by MPLEx.

**Table S3. The metabolomic analysis of EVs by MPLEx**

| **Metabolite** | **Live-BM-EVs** | **Live-BM-EVs** | **Live-BM-EVs** | **Hk-BM-EVs** | **Hk-BM-EVs** | **Hk-BM-EVs** | **Non-BM-EVs** | **Non-BM-EVs** | **Non-BM-EVs** |
| --- | --- | --- | --- | --- | --- | --- | --- | --- | --- |
| capric acid | 10235.9 | 8528.5 | 11514.2 | 18828.3 | 11283.7 | 8705.9 | 13230.7 | 9045.5 | 7549.6 |
| carbonate ion | 5641018.2 | 4647516.7 | 4958849.2 | 5388420.6 | 5040365.3 | 4240979.3 | 3799083.7 | 4653137.7 | 3959612.7 |
| cholesterol | 699562.0 | 286473.0 | 37989.0 | 4313241.0 | 27114.0 | 42249.0 | 35856.0 | 59835.0 | 1087934.0 |
| citric acid | 19917.3 | 12445.9 | 28979.3 | 7144.0 | 16947.2 | 18836.6 | 16982.5 | 17374.7 | 24429.9 |
| D-glucose | 257108.2 | 301988.3 | 1356012.6 | 20451.3 | 259964.5 | 208102.7 | 336458.6 | 178959.6 | 424155.3 |
| diglycerol* | 54962.2 | 116656.4 | 121057.8 | 85886.3 | 33148.8 | 55866.4 | 104745.9 | 120287.3 | 70041.7 |
| glycine | 130349.6 | 330814.7 | 241972.3 | 33915.5 | 113690.2 | 72771.4 | 115944.4 | 118606.3 | 195113.7 |
| lauric acid | 23743.6 | 25905.5 | 26508.8 | 20788.5 | 22414.2 | 15062.9 | 15382.2 | 13717.6 | 38706.7 |
| L-glutamic acid | 79158.6 | 97739.4 | 148788.6 | 34330.6 | 97383.0 | 67390.3 | 121196.0 | 80214.4 | 137359.7 |
| L-leucine | 181820.5 | 293295.6 | 377074.7 | 51025.8 | 209052.9 | 175075.3 | 224495.0 | 170445.0 | 260741.3 |
| L-phenylalanine | 20835.2 | 56622.8 | 112693.6 | 1823.4 | 29668.6 | 20503.4 | 26702.7 | 20496.1 | 42417.8 |
| L-pyroglutamic acid | 361964.7 | 924237.7 | 1149349.4 | 58320.3 | 403195.3 | 318393.1 | 363039.3 | 316537.2 | 754700.6 |
| L-threonine | 19451.1 | 50041.7 | 58053.3 | 0.0 | 23858.4 | 16374.1 | 21440.9 | 16772.3 | 32680.4 |
| L-tyrosine | 29462.0 | 56673.9 | 99978.9 | 0.0 | 39898.7 | 17944.3 | 23863.8 | 14060.7 | 58554.5 |
| L-valine | 70187.4 | 186114.9 | 303808.8 | 19464.6 | 90888.5 | 64273.2 | 92467.8 | 58218.6 | 135680.5 |
| monopalmitin | 502176.3 | 438628.0 | 410228.6 | 445155.6 | 141695.6 | 512114.4 | 131042.4 | 426999.8 | 386109.6 |
| monostearin | 788231.3 | 748448.4 | 518450.8 | 735825.9 | 134907.9 | 746213.2 | 214748.1 | 652191.0 | 608307.8 |
| myo-inositol | 42079.3 | 26486.8 | 61387.4 | 0.0 | 40896.8 | 30190.5 | 41405.4 | 38284.4 | 46802.8 |
| myristic acid | 1940653.0 | 1630707.6 | 977905.6 | 4108000.6 | 818027.9 | 713753.7 | 1409753.9 | 904524.7 | 1905939.0 |
| nanonoic acid | 325222.1 | 292455.9 | 368141.7 | 318394.8 | 297663.8 | 371957.7 | 295242.1 | 311133.6 | 320959.7 |
| phosphate ion | 18335746.9 | 17946461.9 | 30350291.7 | 10496879.2 | 20553716.6 | 20529260.0 | 25513017.7 | 18527411.6 | 27726396.6 |
| Unknown 001 | 127354.8 | 191715.2 | 187448.5 | 35337.3 | 55688.0 | 179350.4 | 152097.6 | 100060.5 | 230216.7 |
| Unknown 002 | 87382.3 | 29452.6 | 64907.1 | 13841.1 | 16236.5 | 49050.0 | 33955.8 | 27775.1 | 40160.2 |
| Unknown 003 | 3029.8 | 1327.1 | 111300.9 | 91546.0 | 95127.5 | 134784.1 | 5948.7 | 101494.0 | 132301.6 |
| Unknown 004 | 500421.4 | 383815.1 | 477882.2 | 511880.0 | 461113.4 | 525037.1 | 451471.8 | 508144.4 | 448101.5 |
| Unknown 005 | 57289.8 | 52685.6 | 166258.9 | 38865.2 | 88813.5 | 132481.7 | 45969.9 | 67071.1 | 95328.8 |
| Unknown 006 | 21243.8 | 22436.2 | 28094.1 | 9353.9 | 26301.4 | 697.7 | 15110.5 | 21583.4 | 36519.9 |
| Unknown 007 | 27457.5 | 31352.9 | 43766.6 | 22159.2 | 31977.5 | 20260.3 | 28690.9 | 20321.5 | 45448.1 |
| Unknown 008 | 13627.9 | 14408.8 | 18420.2 | 9986.3 | 20212.9 | 11726.7 | 13942.2 | 11740.4 | 23545.1 |
| Unknown 009 | 232311.4 | 209697.7 | 205135.6 | 294678.4 | 217993.0 | 210021.0 | 178129.8 | 260271.1 | 211791.9 |
| Unknown 010 | 66756.7 | 52251.4 | 51657.3 | 85508.0 | 75351.1 | 99906.0 | 53869.3 | 59727.8 | 102293.7 |
| Unknown 011 | 15378.0 | 14940.7 | 24176.9 | 37437.1 | 44063.4 | 23940.9 | 22400.7 | 33093.4 | 31588.4 |
| Unknown 012 | 57430.0 | 67313.2 | 76852.1 | 58864.8 | 49702.6 | 53864.8 | 62924.0 | 54798.5 | 71635.1 |
| Unknown 013 | 18184.0 | 14740.2 | 17717.3 | 13737.7 | 14687.8 | 15563.1 | 12536.2 | 26558.1 | 13207.5 |
| Unknown 014 | 8396.4 | 5245.0 | 14311.1 | 8422.9 | 18031.8 | 12609.9 | 9615.1 | 12118.8 | 8768.4 |
| Unknown 015 | 142702.7 | 139654.1 | 181307.5 | 115319.6 | 177663.7 | 165376.7 | 163502.2 | 170923.1 | 138841.7 |
| Unknown 016 | 19935.4 | 20215.3 | 24569.0 | 19743.8 | 19969.0 | 21335.4 | 23053.9 | 20198.8 | 23609.3 |
| Unknown 017 | 64830.3 | 67354.4 | 102092.0 | 45054.2 | 67940.2 | 66027.8 | 77040.0 | 72169.0 | 106453.7 |
| Unknown 018 | 7013.9 | 16986.5 | 15411.1 | 404.0 | 11029.5 | 11452.3 | 13210.1 | 15742.7 | 12502.1 |
| Unknown 019 | 70981.7 | 233856.6 | 296976.6 | 139783.4 | 68793.0 | 72547.1 | 269734.4 | 313575.0 | 251992.1 |
| Unknown 020 | 11637.4 | 330388.4 | 21119.3 | 174786.1 | 62718.3 | 150272.0 | 125902.0 | 65655.2 | 15528.0 |
| Unknown 021 | 17407.3 | 16242.0 | 16174.6 | 15667.7 | 17165.0 | 34529.4 | 13767.6 | 18765.0 | 15182.6 |
| Unknown 022 | 24152.2 | 20053.6 | 167248.0 | 0.0 | 25949.7 | 33340.6 | 35388.1 | 19335.5 | 89279.1 |
| Unknown 023 | 8537.5 | 4268.8 | 12901.4 | 1899.6 | 6724.0 | 8241.3 | 9160.3 | 6423.8 | 5727.7 |
| Unknown 024 | 203606.6 | 193646.8 | 810398.4 | 2407.4 | 264720.4 | 197339.5 | 236232.7 | 158287.1 | 381205.0 |
| Unknown 025 | 5833.0 | 4401.0 | 11135.0 | 3454.6 | 7581.6 | 9277.9 | 6995.1 | 6161.3 | 12080.1 |
| Unknown 026 | 78387.6 | 86746.2 | 94246.4 | 122872.2 | 109910.2 | 85280.7 | 95857.3 | 105998.4 | 70834.1 |
| Unknown 027 | 86275.5 | 92102.8 | 149459.8 | 54661.9 | 71276.1 | 88957.4 | 18918.7 | 80855.0 | 177511.0 |
| Unknown 028 | 23092.2 | 14228.2 | 12012.1 | 29292.5 | 8416.2 | 16873.8 | 7652.9 | 16993.0 | 7246.5 |
| Unknown 029 | 203429.3 | 107911.2 | 124201.3 | 134624.8 | 49043.5 | 59111.4 | 99770.4 | 92400.6 | 88310.8 |
| Unknown 030 | 5236.4 | 75132.0 | 14835.3 | 2614.3 | 91349.9 | 23978.9 | 95086.2 | 111162.0 | 160499.0 |
| Unknown 031 | 57807.3 | 41674.2 | 26521.6 | 29096.8 | 46884.7 | 16028.6 | 73311.1 | 9746.1 | 26910.0 |
| Unknown 032 | 15850.4 | 47190.2 | 84633.0 | 47737.3 | 63032.4 | 34777.5 | 7410.9 | 72999.1 | 86663.1 |
| Unknown 033 | 22915.4 | 188950.7 | 240928.0 | 114027.1 | 162256.9 | 22624.3 | 176264.3 | 307906.5 | 31375.1 |
| Unknown 034 | 230450.0 | 47178.8 | 250832.0 | 146918.2 | 133062.7 | 130074.0 | 214198.4 | 89808.8 | 123912.9 |
| Unknown 035 | 47825.8 | 121772.0 | 63315.0 | 95116.3 | 50058.1 | 27408.5 | 11749.5 | 166983.6 | 107901.5 |
| Unknown 036 | 26764.0 | 43660.0 | 44695.8 | 12325.1 | 37335.6 | 16467.6 | 52577.5 | 80702.1 | 6217.1 |
| Unknown 037 | 114402.0 | 148055.7 | 63477.4 | 9869.2 | 58130.5 | 214402.2 | 151273.0 | 199971.0 | 102321.5 |
| Unknown 038 | 101781.9 | 12964.9 | 6971.5 | 94498.6 | 39128.5 | 67139.0 | 86660.5 | 127650.1 | 27295.2 |

Values are the raw data of spectral counts.

Live-BM-EVs: EVs from live *C. neoformans* infected activated BMDMs;

Hk-BM-EVs: EVs from heat-killed *C. neoformans* infected activated BMDMs;

Non-BM-EVs: EVs from activated BMDMs without *C. neoformans* infection;

Hk: heat-killed.
