# Supplementary material for: Cryptococcus neoformans-Infected Macrophages Release Proinflammatory Extracellular Vesicles: Insight into Their Components by Multi-omics
Source: mBio. 2021 Mar 30;12(2):e00279-21. doi: 10.1128/mBio.00279-21 (PMC8092229; doi:10.1128/mBio.00279-21)
Supplement: TABLE S4 [file mBio.00279-21-st004.docx]

Table S4. Core proteins widely present in all EVs from macrophages.

.

**Table S4. Core proteins present in macrophage EVs from all conditions**

|  | |  | |  | | **Normalized/inputed** | | | | | | | | | |  |
| --- | --- | --- | --- | --- | --- | --- | --- | --- | --- | --- | --- | --- | --- | --- | --- | --- |
| **Protein ID** | **Gene Name** | | **ANOVA** | | **live-BM-EVs** | | **live-BM-EVs** | **live-BM-EVs** | **hk-BM-EVs** | **hk-BM-EVs** | **hk-BM-EVs** | **Non-BM-EVs** | **Non-BM-EVs** | **Non-BM-EVs** |  |  |
| A0A087WR50 | Fn1 | | 0.76 | | 38.88 | | 38.93 | 39.73 | 38.73 | 38.61 | 39.5 | 39.04 | 38.78 | 39.15 |  |  |
| E9PZ16 | Hspg2 | | 0.62 | | 37.45 | | 37.4 | 38.27 | 37.19 | 36.92 | 38.04 | 37.47 | 37.31 | 37.43 |  |  |
| Q8K482 | Emilin2 | | 0.8 | | 35.63 | | 35.74 | 36.15 | 35.5 | 35.21 | 36.24 | 35.77 | 35.6 | 35.85 |  |  |
| P62806 | Hist1h4a | | 0.95 | | 34.98 | | 35.93 | 34.64 | 34.99 | 35.21 | 35.22 | 35 | 35.04 | 35.22 |  |  |
| P60710 | Actb | | 0.8 | | 34.92 | | 35.29 | 35.14 | 35.03 | 35.32 | 35.24 | 35.14 | 35.04 | 35.44 |  |  |
| Q8CBB6 | Hist1h2br | | 0.74 | | 34.82 | | 35.07 | 34.48 | 34.62 | 34.71 | 34.61 | 34.89 | 34.81 | 34.46 |  |  |
| E9QPX1 | Col18a1 | | 0.42 | | 34.79 | | 34.8 | 35.36 | 34.39 | 34.09 | 35.23 | 34.56 | 34.45 | 34.78 |  |  |
| E9PWQ3 | Col6a3 | | 0.15 | | 34.5 | | 34.9 | 35.52 | 33.81 | 33.6 | 34.83 | 34.35 | 34.42 | 34.4 |  |  |
| Q5SXR6 | Cltc | | 0.8 | | 34.41 | | 33.99 | 33.64 | 34.12 | 34.29 | 33.98 | 34.25 | 34.46 | 33.81 |  |  |
| Q07797 | Lgals3bp | | 0.86 | | 34.4 | | 34.84 | 35.18 | 34.35 | 34.8 | 34.85 | 34.5 | 34.46 | 35.1 |  |  |
| A0A1W2P768 | Hist1h3b | | 0.86 | | 34.29 | | 35.25 | 34.68 | 34.67 | 34.7 | 34.58 | 34.78 | 34.67 | 34.87 |  |  |
| P63017 | Hspa8 | | 0.72 | | 34.26 | | 34.51 | 34.26 | 34.21 | 34.48 | 34.81 | 34.35 | 34.27 | 34.68 |  |  |
| Q9R118 | Htra1 | | 0.76 | | 34.24 | | 34.52 | 34.99 | 34.44 | 34.75 | 34.77 | 34.44 | 34.03 | 34.86 |  |  |
| P68433 | Hist1h3a | | 0.19 | | 34.17 | | 34.75 | 33.55 | 33.97 | 32.13 | 33.13 | 33.05 | 33.07 | 33.75 |  |  |
| Q64523 | Hist2h2ac | | 0.89 | | 34.13 | | 34.43 | 34.12 | 33.92 | 34.44 | 34.21 | 34.26 | 34.26 | 33.9 |  |  |
| Q9JLZ6 | Hic2 | | 0.57 | | 34.12 | | 33.99 | 33.78 | 33.89 | 34.15 | 34.27 | 34.01 | 34.01 | 34.15 |  |  |
| Q9WU78 | Pdcd6ip | | 0.96 | | 33.94 | | 34.13 | 33.96 | 33.74 | 33.83 | 34.51 | 33.93 | 33.95 | 34.35 |  |  |
| Q9WV91 | Ptgfrn | | 0.89 | | 33.93 | | 34.21 | 34 | 33.85 | 33.98 | 34.61 | 33.93 | 34.07 | 34.39 |  |  |
| Q99JR5 | Tinagl1 | | 0.62 | | 33.78 | | 33.65 | 34.41 | 33.52 | 33.05 | 34.26 | 33.71 | 33.61 | 33.78 |  |  |
| A2AJY2 | Col15a1 | | 0.52 | | 33.73 | | 33.89 | 33.99 | 33.52 | 33.14 | 34.16 | 33.52 | 33.36 | 33.82 |  |  |
| P10126 | Eef1a1 | | 0.87 | | 33.52 | | 33.55 | 33.34 | 33.36 | 33.76 | 33.46 | 33.52 | 33.68 | 33.41 |  |  |
| P21956 | Mfge8 | | 0.75 | | 33.52 | | 33.1 | 34.06 | 33.19 | 33.8 | 33.95 | 33.19 | 33.48 | 33.56 |  |  |
| P10404 |  | | 0.79 | | 33.42 | | 32.53 | 33.11 | 32.97 | 33.07 | 33.74 | 33.26 | 33.54 | 32.69 |  |  |
| P35762 | Cd81 | | 0.99 | | 33.41 | | 33.09 | 33.41 | 32.66 | 33.5 | 33.84 | 33.71 | 33.27 | 33.03 |  |  |
| P62242 | Rps8 | | 0.33 | | 33.28 | | 32.91 | 32.92 | 32.93 | 32.77 | 33.12 | 32.84 | 32.9 | 32.71 |  |  |
| Q05793 | Hspg2 | | 0.37 | | 33.26 | | 33.15 | 33.78 | 32.9 | 32.35 | 33.61 | 32.99 | 32.91 | 32.94 |  |  |
| P16858 | Gapdh | | 0.98 | | 33.12 | | 33.4 | 33.29 | 33.11 | 33.57 | 33.08 | 33.31 | 33.13 | 33.28 |  |  |
| Q9JHU4 | Dync1h1 | | 0.57 | | 33.05 | | 33.11 | 32.97 | 33.06 | 33.46 | 33.04 | 33.26 | 32.98 | 33.16 |  |  |
| P58022 | Loxl2 | | 0.14 | | 33.04 | | 32.8 | 33.4 | 32.49 | 32.33 | 33.14 | 32.51 | 32.66 | 32.28 |  |  |
| P05213 | Tuba1b | | 0.42 | | 33.01 | | 32.78 | 33.18 | 33.19 | 33.28 | 33 | 33.2 | 33 | 33.17 |  |  |
| Q04857 | Col6a1 | | 0.14 | | 32.99 | | 33.15 | 33.78 | 32.34 | 31.96 | 33.18 | 32.91 | 32.92 | 32.89 |  |  |
| Q3TMX0 | Sdcbp | | 0.77 | | 32.86 | | 32.94 | 33.05 | 32.09 | 32.93 | 33.19 | 32.87 | 32.63 | 33.15 |  |  |
| P26039 | Tln1 | | 0.92 | | 32.85 | | 33.21 | 33.33 | 32.92 | 33.6 | 33.12 | 33.28 | 32.99 | 33.25 |  |  |
| Q9EQK5 | Mvp | | 0.8 | | 32.8 | | 32.94 | 31.58 | 32.31 | 32.69 | 32.97 | 32.65 | 32.68 | 32.66 |  |  |
| P37889 | Fbln2 | | 0.7 | | 32.8 | | 32.42 | 33.86 | 32.77 | 32.28 | 33.2 | 32.85 | 32.5 | 32.71 |  |  |
| E9PYH0 | Vcan | | 0.9 | | 32.79 | | 32.49 | 33.37 | 32.78 | 32.24 | 33.41 | 33.09 | 32.91 | 32.9 |  |  |
| P68372 | Tubb4b | | 0.37 | | 32.74 | | 32.44 | 32.47 | 32.59 | 33.09 | 32.64 | 32.86 | 32.71 | 32.63 |  |  |
| P62983 | Rps27a | | 0.3 | | 32.55 | | 32.96 | 32.42 | 32.61 | 32.63 | 32.9 | 32.35 | 32.22 | 32.66 |  |  |
| E9PVA8 | Gcn1l1 | | 0.17 | | 32.55 | | 32.36 | 32.05 | 32.4 | 32.8 | 32.54 | 32.73 | 32.49 | 33 |  |  |
| P52480 | Pkm | | 0.98 | | 32.54 | | 32.92 | 33.01 | 32.54 | 33.09 | 32.72 | 32.73 | 32.7 | 32.96 |  |  |
| Q99K41 | Emilin1 | | 0.86 | | 32.54 | | 32.82 | 33.77 | 32.54 | 32.47 | 33.47 | 32.94 | 32.96 | 33.04 |  |  |
| P35700 | Prdx1 | | 0.69 | | 32.49 | | 32.32 | 31.81 | 32.42 | 33.2 | 31.87 | 32.83 | 32.79 | 32.02 |  |  |
| Q61398 | Pcolce | | 0.45 | | 32.45 | | 32.87 | 33.33 | 32.35 | 32.69 | 33 | 32.72 | 32.51 | 32.32 |  |  |
| Q9CPX4 | Ftl1 | | 0.38 | | 32.42 | | 32.18 | 32.61 | 33.23 | 33.08 | 32.05 | 32.53 | 32.25 | 32.2 |  |  |
| P27659 | Rpl3 | | 0.56 | | 32.41 | | 32.17 | 31.87 | 31.9 | 31.46 | 32.3 | 32.03 | 32.05 | 31.9 |  |  |
| P09528 | Fth1 | | 0.82 | | 32.39 | | 32.21 | 32.72 | 32.85 | 33.34 | 31.79 | 32.87 | 32.35 | 32.79 |  |  |
| Q9D8E6 | Rpl4 | | 0.05 | | 32.37 | | 32.08 | 32.1 | 31.74 | 31.5 | 31.94 | 31.68 | 31.97 | 31.47 |  |  |
| Q02788 | Col6a2 | | 0.19 | | 32.3 | | 32.71 | 33.39 | 31.59 | 31.51 | 32.7 | 32.34 | 32.43 | 32.02 |  |  |
| P80314 | Cct2 | | 0.81 | | 32.3 | | 32.65 | 32.23 | 32.23 | 32.82 | 32.44 | 32.59 | 32.66 | 32.28 |  |  |
| P11499 | Hsp90ab1 | | 0.27 | | 32.28 | | 32.83 | 32.69 | 32.56 | 33 | 32.56 | 33.17 | 32.58 | 33.55 |  |  |
| P07356 | Anxa2 | | 0.64 | | 32.24 | | 31.91 | 31.31 | 32.22 | 32.39 | 31.85 | 32.66 | 32.53 | 31.37 |  |  |
| P20152 | Vim | | 0.45 | | 32.14 | | 32.12 | 32.27 | 32.14 | 32.27 | 32.7 | 32.22 | 32.33 | 32.56 |  |  |
| P17182 | Eno1 | | 0.28 | | 32.1 | | 32.44 | 32.42 | 32.46 | 32.59 | 32.23 | 32.25 | 32.18 | 32.19 |  |  |
| P14148 | Rpl7 | | 0.11 | | 32.01 | | 32.07 | 31.58 | 31.68 | 31.35 | 31.67 | 31.58 | 31.28 | 31.55 |  |  |
| P12970 | Rpl7a | | 0.14 | | 31.99 | | 32.31 | 31.73 | 31.8 | 31.47 | 31.79 | 31.55 | 31.6 | 31.78 |  |  |
| P47911 | Rpl6 | | 0.13 | | 31.96 | | 31.63 | 31.3 | 31.26 | 31.43 | 31.43 | 31.17 | 31.34 | 31.18 |  |  |
| Q8VDD5 | Myh9 | | 0.72 | | 31.95 | | 31.72 | 31.8 | 31.54 | 31.69 | 31.97 | 31.77 | 31.79 | 31.64 |  |  |
| P20029 | Hspa5 | | 0.72 | | 31.94 | | 31.98 | 31.69 | 31.86 | 31.81 | 31.81 | 31.76 | 31.95 | 32.01 |  |  |
| P80318 | Cct3 | | 0.56 | | 31.9 | | 32.67 | 32.19 | 32.38 | 32.57 | 32.37 | 32.45 | 32.42 | 32.44 |  |  |
| Q01853 | Vcp | | 0.83 | | 31.88 | | 31.11 | 32.68 | 31.49 | 31.37 | 32.25 | 31.84 | 31.45 | 31.61 |  |  |
| Q9CZM2 | Rpl15 | | 0.42 | | 31.87 | | 31.95 | 31.69 | 31.56 | 31.21 | 31.91 | 31.49 | 31.82 | 31.7 |  |  |
| P35980 | Rpl18 | | 0.13 | | 31.85 | | 31.53 | 31.35 | 31.35 | 31.08 | 31.48 | 31.3 | 31.12 | 31.22 |  |  |
| B7FAU9 | Flna | | 0.42 | | 31.83 | | 32.25 | 31.61 | 32.01 | 32.56 | 31.88 | 32.3 | 32.15 | 32.17 |  |  |
| P25444 | Rps2 | | 0.09 | | 31.8 | | 31.82 | 31.83 | 31.53 | 31.67 | 31.74 | 31.73 | 31.73 | 31.56 |  |  |
| P41731 | Cd63 | | 0.91 | | 31.7 | | 31.1 | 31.24 | 31.21 | 31.33 | 31.36 | 30.98 | 31.63 | 31.14 |  |  |
| P19096 | Fasn | | 0.94 | | 31.7 | | 32.12 | 32.18 | 31.93 | 32.36 | 31.86 | 32.14 | 31.84 | 32.21 |  |  |
| P11983 | Tcp1 | | 0.34 | | 31.68 | | 31.93 | 31.64 | 32.07 | 32.13 | 31.71 | 32.25 | 32.17 | 31.7 |  |  |
| P82198 | Tgfbi | | 0.78 | | 31.67 | | 32.28 | 33.05 | 31.82 | 31.77 | 32.76 | 32.35 | 32.18 | 32.72 |  |  |
| Q8HWB2 | H2-Q4 | | 0.3 | | 31.66 | | 31.16 | 31.76 | 31.18 | 31.01 | 31.44 | 30.58 | 30.34 | 31.71 |  |  |
| P15864 | Hist1h1c | | 0.97 | | 31.63 | | 31.7 | 30.6 | 31.3 | 32.23 | 30.04 | 31.72 | 31.81 | 29.93 |  |  |
| P50247 | Ahcy | | 0.85 | | 31.6 | | 31.83 | 32.11 | 31.96 | 32.21 | 31.77 | 31.91 | 31.52 | 32.28 |  |  |
| Q640N1 | Aebp1 | | 0.79 | | 31.58 | | 31.67 | 32.11 | 31.49 | 31.52 | 31.94 | 31.91 | 31.63 | 31.62 |  |  |
| P80316 | Cct5 | | 0.37 | | 31.58 | | 31.78 | 31.32 | 31.53 | 32.17 | 31.75 | 32.08 | 31.83 | 31.67 |  |  |
| A0A0R4J0I9 | Lrp1 | | 0.93 | | 31.57 | | 31.85 | 32.34 | 31.86 | 32.17 | 31.99 | 31.93 | 31.63 | 32.23 |  |  |
| P80315 | Cct4 | | 0.11 | | 31.56 | | 31.92 | 31.31 | 31.82 | 32.27 | 31.92 | 32.2 | 32.16 | 31.84 |  |  |
| P42932 | Cct8 | | 0.06 | | 31.55 | | 31.82 | 31.13 | 31.69 | 31.94 | 31.85 | 32.26 | 32.2 | 31.85 |  |  |
| P11276 | Fn1 | | 0.66 | | 31.54 | | 30.94 | 32.49 | 31.38 | 29.9 | 32.42 | 31.96 | 31.84 | 31.83 |  |  |
| P48036 | Anxa5 | | 0.39 | | 31.42 | | 31.21 | 30.37 | 31.19 | 30.65 | 31.02 | 31.85 | 31.85 | 30.8 |  |  |
| P29477 | Nos2 | | 0.98 | | 31.41 | | 31.14 | 30.52 | 30.87 | 31.32 | 30.65 | 31.37 | 31.37 | 30.2 |  |  |
| Q8VEK3 | Hnrnpu | | 0.27 | | 31.34 | | 30.93 | 31.06 | 30.61 | 30.34 | 30.99 | 31.03 | 31.06 | 30.36 |  |  |
| P62137 | Ppp1ca | | 0.74 | | 31.32 | | 29.98 | 31.13 | 30.42 | 30.32 | 30.81 | 30.86 | 30.75 | 30.41 |  |  |
| P62754 | Rps6 | | 0.32 | | 31.31 | | 31.05 | 31.11 | 30.52 | 31.06 | 31.15 | 30.94 | 31 | 30.61 |  |  |
| P17742 | Ppia | | 0.9 | | 31.29 | | 30.72 | 31.25 | 31.08 | 31.15 | 31.22 | 31.17 | 31.07 | 31 |  |  |
| Q8VHY0 | Cspg4 | | 0.27 | | 31.28 | | 31.36 | 31.17 | 31.23 | 34.26 | 32.04 | 31.38 | 31.51 | 31.46 |  |  |
| E9Q600 | Loxl4 | | 0.6 | | 31.27 | | 30.84 | 31.28 | 30.86 | 30.21 | 31.36 | 31.08 | 30.95 | 30.74 |  |  |
| Q6ZWN5 | Rps9 | | 0.95 | | 31.26 | | 31.82 | 31.11 | 31.33 | 31.17 | 31.49 | 31.21 | 31.05 | 31.71 |  |  |
| Q9DCK3 | Tspan4 | | 0.42 | | 31.23 | | 30.28 | 31.12 | 31.01 | 31.33 | 31.79 | 31.16 | 31.36 | 30.54 |  |  |
| P58252 | Eef2 | | 0.83 | | 31.19 | | 31.67 | 31.39 | 31.15 | 31.48 | 31.36 | 31.31 | 31.29 | 31.7 |  |  |
| E0CZ27 | H3f3a | | 0.35 | | 31.17 | | 32.32 | 31.28 | 31.65 | 29.52 | 30.9 | 30.5 | 31.08 | 31.04 |  |  |
| P80317 | Cct6a | | 0.21 | | 31.12 | | 31.75 | 30.97 | 31.64 | 31.96 | 31.33 | 31.94 | 31.71 | 31.68 |  |  |
| P09411 | Pgk1 | | 0.97 | | 31.12 | | 31.41 | 31.88 | 31.39 | 31.74 | 31.4 | 31.42 | 31.35 | 31.79 |  |  |
| Q3THW5 | H2afv | | 0.14 | | 31.1 | | 32.11 | 31.63 | 31.25 | 30.67 | 30.51 | 31.45 | 30.87 | 31.51 |  |  |
| P62908 | Rps3 | | 0.45 | | 31.05 | | 30.79 | 30.9 | 30.96 | 31.23 | 30.84 | 30.99 | 30.71 | 30.81 |  |  |
| P10107 | Anxa1 | | 0.64 | | 31.01 | | 30.64 | 28.99 | 30.81 | 30.99 | 30.08 | 31.48 | 31.41 | 29.8 |  |  |
| P23116 | Eif3a | | 0.65 | | 31.01 | | 31.4 | 30.47 | 31.15 | 31.5 | 30.8 | 31.76 | 31.45 | 30.73 |  |  |
| P05064 | Aldoa | | 0.53 | | 31.01 | | 31.51 | 31.13 | 30.8 | 31.37 | 31.28 | 31.5 | 31.37 | 31.26 |  |  |
| P97351 | Rps3a | | 0.39 | | 30.99 | | 30.6 | 30.94 | 30.7 | 30.83 | 30.47 | 30.65 | 30.81 | 30.25 |  |  |
| P68033 | Actc1 | | 0.68 | | 30.97 | | 30.75 | 30.63 | 30.51 | 30.88 | 30.58 | 30.81 | 30.56 | 30.85 |  |  |
| P97429 | Anxa4 | | 0.68 | | 30.94 | | 30.54 | 31.29 | 30.92 | 30.65 | 31.86 | 31.15 | 31.11 | 31.44 |  |  |
| P63101 | Ywhaz | | 0.46 | | 30.9 | | 31.08 | 30.48 | 30.95 | 30.88 | 31.49 | 31.01 | 30.9 | 31.35 |  |  |
| Q9CR57 | Rpl14 | | 0.2 | | 30.89 | | 30.67 | 30.6 | 30.71 | 30.34 | 30.31 | 30.44 | 30.65 | 30.4 |  |  |
| A0A1D5RLW5 | Rpl18a | | 0.32 | | 30.87 | | 30.89 | 30.39 | 30.66 | 30.23 | 30.6 | 30.21 | 30.54 | 30.47 |  |  |
| P97298 | Serpinf1 | | 0.71 | | 30.86 | | 31.57 | 31.78 | 31.01 | 30.8 | 31.31 | 31.07 | 30.57 | 31.99 |  |  |
| Q62351 | Tfrc | | 0.96 | | 30.82 | | 30.58 | 30.1 | 30.32 | 30.05 | 31.04 | 30.34 | 30.38 | 30.55 |  |  |
| Q61696 | Hspa1a | | 0.19 | | 30.82 | | 30.97 | 31.01 | 30.7 | 30.75 | 30.9 | 30.58 | 30.54 | 30.93 |  |  |
| Q8CGC7 | Eprs | | 0.12 | | 30.82 | | 31.2 | 30.86 | 30.78 | 31.01 | 30.94 | 31.23 | 31.17 | 31.14 |  |  |
| P11087 | Col1a1 | | 0.59 | | 30.81 | | 31.03 | 31.36 | 30.68 | 29.66 | 31.4 | 30.9 | 30.54 | 31.32 |  |  |
| I7HLV2 | Rpl10 | | 0.22 | | 30.8 | | 30.84 | 30.61 | 30.57 | 30.22 | 30.66 | 30.47 | 30.57 | 30.74 |  |  |
| P80313 | Cct7 | | 0.58 | | 30.79 | | 31.45 | 31.02 | 31.12 | 31.52 | 31.1 | 31.5 | 31.3 | 31.14 |  |  |
| Q06890 | Clu | | 0.99 | | 30.76 | | 31.38 | 31.92 | 31.27 | 31.47 | 31.36 | 31.33 | 30.72 | 31.99 |  |  |
| P08752 | Gnai2 | | 0.71 | | 30.75 | | 30 | 30.02 | 30.3 | 30.38 | 30.4 | 30.82 | 31 | 29.85 |  |  |
| P97449 | Anpep | | 0.91 | | 30.74 | | 30.07 | 29.86 | 30.62 | 30.29 | 30.3 | 31.22 | 31.22 | 29.04 |  |  |
| Q99JI6 | Rap1b | | 0.71 | | 30.69 | | 30.55 | 30.84 | 30.63 | 30.87 | 30.84 | 30.86 | 30.63 | 30.72 |  |  |
| P99024 | Tubb5 | | 0.46 | | 30.66 | | 30.64 | 30.25 | 30.38 | 30.86 | 30.69 | 30.7 | 30.99 | 30.6 |  |  |
| Q8BU30 | Iars | | 0.73 | | 30.64 | | 30.77 | 30.71 | 30.51 | 30.8 | 30.77 | 30.75 | 31.13 | 30.56 |  |  |
| P60843 | Eif4a1 | | 0.57 | | 30.62 | | 31.08 | 30.67 | 30.7 | 30.7 | 30.49 | 30.7 | 30.63 | 31.08 |  |  |
| P16045 | Lgals1 | | 0.95 | | 30.59 | | 30.11 | 30.39 | 30.33 | 30.7 | 30.32 | 30.74 | 30.65 | 29.77 |  |  |
| P14869 | Rplp0 | | 0.06 | | 30.59 | | 30.74 | 30.82 | 29.98 | 30.57 | 30.35 | 29.96 | 30.44 | 30.07 |  |  |
| Q3UER8 | Fgg | | 0.9 | | 30.56 | | 31.04 | 31.37 | 30.85 | 31.29 | 31.06 | 31.11 | 30.93 | 31.22 |  |  |
| Q61838 | A2m | | 0.7 | | 30.55 | | 30.27 | 32.13 | 29.5 | 31.8 | 30.24 | 30.6 | 30.23 | 30.31 |  |  |
| Q01149 | Col1a2 | | 0.96 | | 30.52 | | 30.43 | 30.75 | 30.3 | 30.39 | 31.1 | 30.58 | 30.5 | 30.8 |  |  |
| P14824 | Anxa6 | | 0.58 | | 30.52 | | 30 | 31.17 | 30.42 | 30.18 | 31.56 | 31.01 | 30.83 | 31.31 |  |  |
| P29341 | Pabpc1 | | 0.56 | | 30.5 | | 30.45 | 29.7 | 30.24 | 30.54 | 30.6 | 30.55 | 30.53 | 30.25 |  |  |
| P84078 | Arf1 | | 0.83 | | 30.48 | | 30.47 | 30.47 | 30.34 | 30.41 | 30.48 | 30.49 | 30.2 | 30.64 |  |  |
| E9QAI5 | Cad | | 0.4 | | 30.45 | | 30.56 | 30.02 | 30.48 | 30.6 | 30.51 | 30.27 | 30.36 | 30.44 |  |  |
| P62827 | Ran | | 0.42 | | 30.44 | | 30.72 | 31.22 | 30.53 | 31.02 | 30.62 | 30.14 | 30.52 | 30.7 |  |  |
| Q8R1B4 | Eif3c | | 0.57 | | 30.41 | | 30.55 | 28.62 | 30.39 | 30.79 | 29.6 | 31.03 | 30.58 | 30.02 |  |  |
| P62855 | Rps26 | | 0.68 | | 30.39 | | 30.19 | 29.73 | 30.17 | 29.59 | 30.29 | 29.65 | 29.96 | 30.02 |  |  |
| P55065 | Pltp | | 0.75 | | 30.36 | | 31.07 | 31.06 | 30.56 | 30.84 | 30.92 | 30.67 | 30.28 | 30.95 |  |  |
| P04223 | H2-K1 | | 0.05 | | 30.35 | | 29.76 | 29.83 | 31.19 | 31.29 | 29.83 | 29.68 | 29.22 | 29.39 |  |  |
| K3W4T3 | Atp6v0a1 | | 0.16 | | 30.32 | | 29.46 | 31.73 | 30.62 | 30.74 | 30.92 | 30.81 | 27.6 | 27.46 |  |  |
| P62889 | Rpl30 | | 0.25 | | 30.32 | | 29.88 | 30.2 | 29.78 | 29.75 | 29.72 | 29.84 | 30.02 | 28.94 |  |  |
| P28653 | Bgn | | 0.6 | | 30.32 | | 30.06 | 30.26 | 29.85 | 29.44 | 30.53 | 29.98 | 30.23 | 30.25 |  |  |
| P14206 | Rpsa | | 0.61 | | 30.32 | | 30.36 | 30.72 | 30.36 | 31.09 | 30.5 | 30.6 | 30.09 | 30.53 |  |  |
| Q3V117 | Acly | | 0.52 | | 30.31 | | 30.81 | 30.86 | 30.59 | 31.03 | 30.71 | 30.73 | 30.38 | 30.51 |  |  |
| Q91V55 | Rps5 | | 0.6 | | 30.3 | | 30.06 | 29.95 | 30.12 | 30.8 | 29.93 | 30.43 | 30 | 29.37 |  |  |
| P19253 | Rpl13a | | 0.13 | | 30.29 | | 30.24 | 29.87 | 29.91 | 29.52 | 29.87 | 29.77 | 29.97 | 29.61 |  |  |
| P07901 | Hsp90aa1 | | 0.9 | | 30.29 | | 30.77 | 30.79 | 30.65 | 30.85 | 30.4 | 30.61 | 30.41 | 31.16 |  |  |
| P47962 | Rpl5 | | 0.17 | | 30.27 | | 30.29 | 30.28 | 30.36 | 30.13 | 29.85 | 30.04 | 30.06 | 29.54 |  |  |
| Q9Z2U0 | Psma7 | | 0.9 | | 30.26 | | 31.13 | 31.12 | 30.58 | 30.95 | 30.56 | 30.91 | 30.32 | 31.15 |  |  |
| Q8BPB5 | Efemp1 | | 0.88 | | 30.25 | | 30.25 | 31.45 | 30.4 | 30.82 | 30.91 | 30.79 | 30.36 | 30.45 |  |  |
| P62918 | Rpl8 | | 0.22 | | 30.24 | | 29.66 | 30.15 | 30.03 | 30.08 | 30.34 | 29.8 | 29.77 | 29.9 |  |  |
| Q8QZY1 | Eif3l | | 0.6 | | 30.24 | | 30.82 | 29.11 | 30.51 | 30.83 | 29.7 | 30.99 | 30.6 | 30.21 |  |  |
| Q922B2 | Dars | | 0.87 | | 30.21 | | 30.38 | 30.11 | 30.17 | 30.3 | 30.16 | 30.58 | 30.45 | 29.89 |  |  |
| Q9D8B3 | Chmp4b | | 0.21 | | 30.21 | | 30.27 | 29.7 | 29.97 | 30.59 | 30.81 | 30.34 | 30.74 | 30.65 |  |  |
| Q9CX00 | Ist1 | | 0.68 | | 30.21 | | 30.44 | 30.17 | 30.15 | 30.31 | 31.17 | 30.1 | 30.34 | 30.7 |  |  |
| Q9R0E2 | Plod1 | | 0.76 | | 30.21 | | 31.24 | 31.04 | 30.68 | 30.67 | 30.91 | 30.95 | 30.61 | 31.4 |  |  |
| P46467 | Vps4b | | 0.67 | | 30.19 | | 30.14 | 30.08 | 30.45 | 29.85 | 30.67 | 30.08 | 30.25 | 30.77 |  |  |
| P62911 | Rpl32 | | 0.99 | | 30.18 | | 29.27 | 29.33 | 29.65 | 29.32 | 29.87 | 30.03 | 29.54 | 29.28 |  |  |
| Q7TPV4 | Mybbp1a | | 0.69 | | 30.16 | | 29.64 | 29.54 | 29.83 | 29.11 | 29.73 | 30.01 | 29.68 | 29.54 |  |  |
| A2AN08 | Ubr4 | | 0.51 | | 30.16 | | 30.62 | 30.18 | 31.28 | 30.79 | 30.14 | 30.83 | 30.54 | 30.05 |  |  |
| G3X9T8 | Cp | | 0.85 | | 30.16 | | 30.53 | 31.16 | 30.06 | 30.34 | 30.87 | 30.14 | 30.12 | 31.01 |  |  |
| F6YVP7 | Gm10260 | | 0.87 | | 30.15 | | 30.5 | 29.66 | 29.87 | 30.66 | 29.66 | 30.24 | 30 | 29.53 |  |  |
| P68040 | Gnb2l1 | | 0.59 | | 30.15 | | 29.73 | 30.75 | 30.23 | 30.55 | 30.09 | 29.94 | 30 | 30.1 |  |  |
| Q9CQW9 | Ifitm3 | | 0.7 | | 30.13 | | 30.01 | 28.9 | 29.8 | 30.06 | 30.01 | 29.94 | 29.75 | 29.59 |  |  |
| P62281 | Rps11 | | 0.09 | | 30.13 | | 29.97 | 30.34 | 29.79 | 29.98 | 29.82 | 29.8 | 30.03 | 29.88 |  |  |
| P62702 | Rps4x | | 0.68 | | 30.1 | | 30.04 | 30.26 | 30.43 | 30.69 | 29.67 | 30.57 | 30.34 | 30.22 |  |  |
| Q8C483 | Sars | | 0.86 | | 30.1 | | 30.22 | 30.93 | 30.28 | 30.75 | 30.55 | 30.64 | 30.31 | 30.67 |  |  |
| B2RQC7 | Dip2b | | 0.73 | | 30.07 | | 25.73 | 24.28 | 25.41 | 24.1 | 24.25 | 25.61 | 29.6 | 17.5 |  |  |
| Q6ZWZ4 | Rpl36 | | 0.11 | | 30.06 | | 29.71 | 29.65 | 29.23 | 29.15 | 29.53 | 29.63 | 29.72 | 29.17 |  |  |
| Q9CQM8 | Rpl21 | | 0.55 | | 30.05 | | 29.76 | 29.71 | 29.96 | 29.06 | 29.7 | 29.64 | 29.79 | 29.44 |  |  |
| E9PZ00 | Psap | | 0.99 | | 30.04 | | 30.27 | 32.05 | 30.13 | 30.48 | 31.76 | 30.65 | 30.21 | 31.25 |  |  |
| Q564E2 | Ldha | | 0.77 | | 30.03 | | 30.14 | 30.85 | 30.37 | 30.89 | 30.34 | 30.59 | 30.34 | 30.32 |  |  |
| Q9R1P4 | Psma1 | | 0.76 | | 30.03 | | 30.48 | 30.69 | 30.38 | 30.68 | 30.49 | 30.63 | 30.12 | 31.1 |  |  |
| Q6ZWZ7 | Rpl17 | | 0.34 | | 30.02 | | 29.38 | 29.94 | 29.43 | 29.03 | 29.92 | 29.48 | 29.39 | 29.21 |  |  |
| Q8R366 | Igsf8 | | 0.94 | | 30.02 | | 30.25 | 30.08 | 30.06 | 29.91 | 30.57 | 30.04 | 30.14 | 30.3 |  |  |
| P62259 | Ywhae | | 0.51 | | 30.01 | | 29.99 | 29.75 | 29.56 | 29.73 | 30.18 | 30.12 | 29.94 | 30.04 |  |  |
| P62880 | Gnb2 | | 0.68 | | 30 | | 29.92 | 29.28 | 29.72 | 29.8 | 30.34 | 30 | 30.19 | 29.63 |  |  |
| A2A547 | Rpl19 | | 0.33 | | 29.99 | | 29.25 | 29.93 | 28.75 | 29.64 | 29.32 | 29.46 | 29.78 | 29.38 |  |  |
| P62264 | Rps14 | | 0.54 | | 29.97 | | 30.49 | 28.6 | 31.02 | 29.08 | 31 | 30.33 | 30.37 | 30.57 |  |  |
| Q8VDN2 | Atp1a1 | | 0.21 | | 29.96 | | 29.42 | 28.8 | 29.83 | 29.76 | 29.76 | 30.11 | 30.5 | 29.62 |  |  |
| A0A0G2JGD2 | S100a4 | | 0.65 | | 29.94 | | 30.4 | 30.59 | 29.76 | 30.75 | 30.62 | 30.13 | 30.05 | 30.14 |  |  |
| Q61553 | Fscn1 | | 0.85 | | 29.94 | | 30.24 | 30.29 | 30.29 | 30.21 | 30.18 | 30.14 | 29.84 | 30.45 |  |  |
| P18760 | Cfl1 | | 0.96 | | 29.93 | | 29.56 | 29.87 | 30.11 | 29.81 | 29.63 | 30.49 | 30.06 | 29.1 |  |  |
| P14115 | Rpl27a | | 0.64 | | 29.93 | | 29.48 | 29.8 | 29.49 | 29.48 | 29.71 | 29.72 | 29.87 | 29.3 |  |  |
| P17751 | Tpi1 | | 0.99 | | 29.93 | | 29.8 | 29.92 | 29.71 | 30.32 | 29.66 | 29.87 | 29.76 | 30.05 |  |  |
| Q9Z2U1 | Psma5 | | 0.78 | | 29.93 | | 30.37 | 30.62 | 30.02 | 30.6 | 30.55 | 30.43 | 30.14 | 31 |  |  |
| A0A171EBL2 | Rnf213 | | 0.96 | | 29.91 | | 30.06 | 28.92 | 29.82 | 30.16 | 29.3 | 30.21 | 30.16 | 28.82 |  |  |
| P17427 | Ap2a2 | | 0.17 | | 29.91 | | 29.29 | 29.16 | 29.66 | 30.28 | 29.96 | 30.17 | 30.21 | 29.63 |  |  |
| P61358 | Rpl27 | | 0.09 | | 29.91 | | 29.93 | 29.68 | 29.82 | 29.61 | 29.74 | 29.89 | 29.95 | 30.08 |  |  |
| O35639 | Anxa3 | | 0.32 | | 29.88 | | 28.74 | 28.37 | 28.93 | 28.8 | 28.81 | 30.03 | 29.78 | 28.9 |  |  |
| P49722 | Psma2 | | 0.79 | | 29.88 | | 30.67 | 30.72 | 30.52 | 30.87 | 30.58 | 30.68 | 29.76 | 30.9 |  |  |
| Q9QUM9 | Psma6 | | 0.59 | | 29.87 | | 30.51 | 30.53 | 30.45 | 30.69 | 30.39 | 30.52 | 30.21 | 30.99 |  |  |
| Q9DBG3 | Ap2b1 | | 0.42 | | 29.86 | | 29.75 | 29.44 | 29.86 | 29.93 | 29.99 | 30.3 | 30.25 | 29.48 |  |  |
| Q9Z1Q5 | Clic1 | | 0.49 | | 29.84 | | 29.99 | 29.37 | 29.68 | 29.66 | 29.42 | 29.96 | 29.86 | 29.63 |  |  |
| Q9WVK4 | Ehd1 | | 0.19 | | 29.83 | | 30.1 | 30.17 | 29.72 | 30.33 | 30.32 | 29.82 | 29.83 | 29.46 |  |  |
| Q9D0R2 | Tars | | 0.29 | | 29.82 | | 29.41 | 29.33 | 30.34 | 29.55 | 29.95 | 29.72 | 29.82 | 29.28 |  |  |
| P10605 | Ctsb | | 0.53 | | 29.8 | | 30.33 | 30.94 | 29.97 | 30 | 30.41 | 29.76 | 29.66 | 30.42 |  |  |
| Q9Z175 | Loxl3 | | 0.72 | | 29.76 | | 29.47 | 30.25 | 29.36 | 28.98 | 30.27 | 29.49 | 29.71 | 29.68 |  |  |
| Q9QZF2 | Gpc1 | | 0.99 | | 29.76 | | 29.35 | 30.28 | 29.39 | 29.5 | 30.6 | 29.57 | 29.97 | 29.81 |  |  |
| Q8CIE6 | Copa | | 0.09 | | 29.76 | | 30.08 | 29.4 | 29.92 | 30.25 | 30.23 | 30.45 | 30.18 | 30.14 |  |  |
| P08122 | Col4a2 | | 0.89 | | 29.75 | | 28.42 | 30.57 | 29.83 | 28.65 | 29.84 | 29.36 | 29.55 | 28.93 |  |  |
| P26041 | Msn | | 0.86 | | 29.75 | | 29.76 | 29.7 | 29.58 | 29.8 | 29.75 | 29.89 | 29.69 | 29.43 |  |  |
| Q8BH78 | Rtn4 | | 0.95 | | 29.75 | | 30.19 | 30.42 | 29.98 | 29.8 | 30.58 | 29.98 | 30.21 | 30.38 |  |  |
| P35979 | Rpl12 | | 0.6 | | 29.74 | | 29.29 | 29.23 | 29.35 | 29.58 | 29.02 | 29.14 | 29.45 | 29.01 |  |  |
| Q02053 | Uba1 | | 0.19 | | 29.72 | | 29.65 | 29.78 | 29.95 | 30.23 | 29.78 | 30.04 | 30.08 | 29.74 |  |  |
| A0A087WSN6 | Fn1 | | 0.39 | | 29.71 | | 30.01 | 30.02 | 28.72 | 28.87 | 30.29 | 29.6 | 29.58 | 29.92 |  |  |
| Q64282 | Ifit1 | | 0.68 | | 29.69 | | 29.42 | 27.6 | 29.24 | 29.38 | 28.26 | 29.58 | 29.77 | 28.94 |  |  |
| Q9R1P0 | Psma4 | | 0.74 | | 29.69 | | 30.68 | 30.8 | 30.5 | 30.77 | 30.33 | 30.97 | 30.19 | 30.87 |  |  |
| Q9DBJ1 | Pgam1 | | 0.67 | | 29.68 | | 29.59 | 29.59 | 29.61 | 29.59 | 29.39 | 29.66 | 29.62 | 29.33 |  |  |
| Q91VB8 | Hba | | 0.88 | | 29.68 | | 28.73 | 33.61 | 29.63 | 30.3 | 30.58 | 30.22 | 29.57 | 30.45 |  |  |
| P11370 | Fv4 | | 0.73 | | 29.67 | | 27.7 | 27.83 | 28.81 | 28.72 | 29.25 | 28.73 | 29.37 | 26.79 |  |  |
| Q9R045 | Angptl2 | | 0.89 | | 29.67 | | 28.29 | 29.51 | 29.03 | 28.95 | 29.28 | 29.16 | 29.48 | 29.15 |  |  |
| Q3TLP8 | Rac1 | | 0.54 | | 29.66 | | 29.67 | 29.6 | 29.41 | 29.92 | 28.77 | 29.54 | 29.36 | 29.11 |  |  |
| P12815 | Pdcd6 | | 0.79 | | 29.66 | | 29.5 | 30.17 | 29.4 | 29.28 | 30.1 | 29.56 | 29.69 | 29.68 |  |  |
| Q9D8N0 | Eef1g | | 0.91 | | 29.66 | | 30 | 30.3 | 30.13 | 29.87 | 29.72 | 30.05 | 29.74 | 30.08 |  |  |
| O09061 | Psmb1 | | 0.95 | | 29.64 | | 30.45 | 30.56 | 30.29 | 30.29 | 29.77 | 30.16 | 29.76 | 30.68 |  |  |
| P46638 | Rab11b | | 0.73 | | 29.61 | | 28.81 | 28.5 | 29.13 | 29.05 | 29.05 | 29.25 | 28.99 | 29.37 |  |  |
| Q8VDM4 | Psmd2 | | 0.75 | | 29.6 | | 29.82 | 29.19 | 29.51 | 30.03 | 29.6 | 29.78 | 29.71 | 29.24 |  |  |
| A0A1L1SQA8 | Rps25 | | 0.94 | | 29.59 | | 29.09 | 28.35 | 29.04 | 29.35 | 28.13 | 29.5 | 29.26 | 28.25 |  |  |
| Q07113 | Igf2r | | 0.93 | | 29.59 | | 28.48 | 29.79 | 29.28 | 29.38 | 29.56 | 29.44 | 29.39 | 29.26 |  |  |
| Q9DD06 | Rarres2 | | 0.6 | | 29.59 | | 29.73 | 30.09 | 29.77 | 30.12 | 29.88 | 29.82 | 29.73 | 29.78 |  |  |
| Q9JKF1 | Iqgap1 | | 0.54 | | 29.58 | | 29.54 | 28.98 | 29.56 | 29.67 | 29.7 | 29.77 | 29.66 | 29.07 |  |  |
| Q62419 | Sh3gl1 | | 0.87 | | 29.58 | | 29.21 | 29.58 | 29.14 | 29.2 | 29.77 | 29.33 | 29.43 | 29.35 |  |  |
| Q9Z0J7 | Gdf15 | | 0.8 | | 29.58 | | 29.88 | 29.89 | 29.26 | 30.1 | 29.78 | 29.66 | 29.89 | 30.06 |  |  |
| P60766 | Cdc42 | | 0.6 | | 29.57 | | 29.59 | 29.84 | 29.33 | 29.73 | 29.18 | 29.48 | 29.89 | 29 |  |  |
| P14131 | Rps16 | | 0.89 | | 29.55 | | 29.68 | 28.31 | 29.43 | 29.25 | 29.29 | 29.45 | 29.38 | 29.17 |  |  |
| Q8BH64 | Ehd2 | | 0.16 | | 29.55 | | 29.27 | 29.41 | 29.24 | 29.65 | 29.89 | 29.84 | 29.82 | 29.7 |  |  |
| O88342 | Wdr1 | | 0.43 | | 29.55 | | 29.76 | 29.9 | 29.88 | 30.13 | 29.77 | 29.79 | 29.81 | 30.16 |  |  |
| P02463 | Col4a1 | | 0.89 | | 29.53 | | 28.67 | 29.91 | 29.8 | 28.55 | 29.39 | 29.28 | 29.38 | 28.78 |  |  |
| B1AZS9 | Prdx4 | | 0.93 | | 29.52 | | 29.66 | 28.84 | 29.45 | 30.24 | 28.74 | 29.83 | 29.67 | 28.97 |  |  |
| Q5SW88 | Rab1 | | 0.66 | | 29.52 | | 29.34 | 28.48 | 28.82 | 29.05 | 29.26 | 29.11 | 29.3 | 29.52 |  |  |
| O08553 | Dpysl2 | | 0.52 | | 29.52 | | 29.67 | 29.86 | 29.73 | 30.27 | 29.45 | 29.96 | 29.86 | 30 |  |  |
| O70435 | Psma3 | | 0.46 | | 29.51 | | 30.29 | 30.34 | 30.06 | 30.18 | 29.92 | 30.67 | 29.87 | 30.73 |  |  |
| Q8JZQ9 | Eif3b | | 0.73 | | 29.5 | | 29.62 | 28.3 | 28.98 | 29.69 | 29.19 | 29.97 | 29.73 | 28.84 |  |  |
| Q9Z1T2 | Thbs4 | | 0.75 | | 29.49 | | 29.1 | 30.47 | 29.85 | 30.06 | 29.65 | 29.82 | 29.39 | 29.54 |  |  |
| P08113 | Hsp90b1 | | 0.22 | | 29.46 | | 29.42 | 29.78 | 29.62 | 29.71 | 29.95 | 29.73 | 29.77 | 30.3 |  |  |
| P32261 | Serpinc1 | | 0.84 | | 29.43 | | 29.94 | 30.39 | 29.98 | 29.84 | 30.08 | 29.97 | 29.28 | 30.11 |  |  |
| P10852 | Slc3a2 | | 0.88 | | 29.42 | | 29.36 | 28.66 | 28.91 | 28.99 | 29.42 | 29.15 | 29.41 | 29.12 |  |  |
| P17809 | Slc2a1 | | 0.69 | | 29.42 | | 28.76 | 29.12 | 29.05 | 28.71 | 29.38 | 28.34 | 28.96 | 29.23 |  |  |
| Q8C605 | Pfkp | | 0.21 | | 29.41 | | 28.57 | 28.7 | 29.41 | 29.69 | 29.05 | 28.94 | 29.1 | 28.67 |  |  |
| Q8BGQ7 | Aars | | 0.44 | | 29.41 | | 29.8 | 29.57 | 29.1 | 29.81 | 29.22 | 29.87 | 29.47 | 29.66 |  |  |
| Q8BMJ2 | Lars | | 0.22 | | 29.4 | | 29.47 | 28.9 | 29.36 | 29.29 | 29.42 | 30.03 | 29.77 | 29.28 |  |  |
| Q8VEJ9 | Vps4a | | 0.86 | | 29.4 | | 29.6 | 29.37 | 29.04 | 29.52 | 30.28 | 29.29 | 29.6 | 29.97 |  |  |
| P60229 | Eif3e | | 0.57 | | 29.37 | | 29.79 | 28.62 | 29.59 | 29.91 | 28.88 | 30.1 | 29.77 | 29.29 |  |  |
| Q9QYJ0 | Dnaja2 | | 0.85 | | 29.33 | | 29.01 | 28.53 | 29 | 28.88 | 29.51 | 29.26 | 29.43 | 28.6 |  |  |
| P41105 | Rpl28 | | 0.17 | | 29.33 | | 29.16 | 29.15 | 28.64 | 29.13 | 29.46 | 28.64 | 28.89 | 28.77 |  |  |
| Q9R1P1 | Psmb3 | | 0.96 | | 29.31 | | 29.53 | 30.42 | 29.69 | 29.73 | 29.82 | 29.95 | 29.35 | 30.19 |  |  |
| P43276 | Hist1h1b | | 0.87 | | 29.3 | | 29.67 | 28.17 | 28.97 | 29.98 | 27.99 | 29.83 | 29.79 | 28.38 |  |  |
| P54116 | Stom | | 0.95 | | 29.3 | | 29.71 | 28.51 | 28.58 | 29.07 | 29.72 | 29.44 | 29.39 | 28.9 |  |  |
| P11031 | Sub1 | | 0.88 | | 29.29 | | 29.33 | 28.92 | 28.8 | 29.68 | 28.32 | 29.36 | 29.48 | 28.18 |  |  |
| Q08857 | Cd36 | | 0.46 | | 29.29 | | 28.95 | 28.56 | 28.87 | 28.24 | 28.96 | 29.29 | 29.21 | 28.72 |  |  |
| Q61753 | Phgdh | | 0.99 | | 29.29 | | 29.5 | 29.76 | 29.53 | 29.72 | 29.23 | 29.36 | 29.36 | 29.76 |  |  |
| P40124 | Cap1 | | 0.44 | | 29.26 | | 28.69 | 29.1 | 29.27 | 29.74 | 28.55 | 29.93 | 29.76 | 28.96 |  |  |
| P11152 | Lpl | | 0.58 | | 29.26 | | 29.69 | 29.59 | 29.5 | 29.65 | 29.59 | 29.26 | 29.24 | 29.7 |  |  |
| Q9QZQ8 | H2afy | | 0.89 | | 29.25 | | 28.94 | 26.94 | 28.61 | 29.29 | 27.89 | 29.23 | 29.03 | 27.95 |  |  |
| A0A0R4J0Q4 | Loxl1 | | 0.46 | | 29.24 | | 29.4 | 29.46 | 28.86 | 28.92 | 29.52 | 29.36 | 29.17 | 28.73 |  |  |
| A2AE89 | Gstm1 | | 0.64 | | 29.24 | | 29.07 | 29.33 | 28.88 | 29.39 | 29.79 | 29.64 | 29.3 | 29.38 |  |  |
| P24668 | M6pr | | 0.98 | | 29.23 | | 28.89 | 28.52 | 28.77 | 28.91 | 29.18 | 29.08 | 29.41 | 28.29 |  |  |
| A0A1B0GT92 | Gys1 | | 0.97 | | 29.22 | | 28.79 | 28.07 | 28.57 | 28.76 | 28.64 | 28.79 | 29.18 | 27.8 |  |  |
| Q99P91 | Gpnmb | | 0.63 | | 29.22 | | 28.3 | 28.8 | 27.92 | 28.41 | 29.07 | 28.74 | 28.31 | 28.28 |  |  |
| G3UYM8 | Mcf2l | | 0.61 | | 29.22 | | 29.46 | 29.98 | 29.83 | 29.74 | 29.99 | 29.61 | 29.4 | 30.37 |  |  |
| F6YY69 | Ywhaq | | 0.89 | | 29.2 | | 28.78 | 29.4 | 28.9 | 29.49 | 29.39 | 29.48 | 29.46 | 28.77 |  |  |
| Q9WTI7 | Myo1c | | 0.66 | | 29.18 | | 28.96 | 28 | 29.08 | 28.78 | 28.98 | 29 | 29.22 | 28.76 |  |  |
| Q9Z1Q9 | Vars | | 0.82 | | 29.18 | | 29.43 | 29.77 | 29.5 | 29.94 | 29.35 | 29.74 | 29.37 | 29.31 |  |  |
| Q99JX4 | Eif3m | | 0.62 | | 29.17 | | 29.32 | 28.37 | 28.56 | 29.84 | 28.69 | 29.9 | 29.63 | 28.75 |  |  |
| P50516 | Atp6v1a | | 0.08 | | 29.16 | | 28.88 | 28.39 | 29.1 | 29.37 | 28.85 | 29.57 | 29.48 | 29.28 |  |  |
| P62849 | Rps24 | | 0.51 | | 29.14 | | 28.81 | 28.86 | 29 | 28.04 | 28.62 | 29.04 | 28.13 | 28.79 |  |  |
| Q61187 | Tsg101 | | 0.8 | | 29.13 | | 29.25 | 27.46 | 28.99 | 27.45 | 29.53 | 28.93 | 29.1 | 29.08 |  |  |
| A0A0A0MQA5 | Tuba4a | | 0.94 | | 29.13 | | 29.17 | 29.3 | 29.44 | 29.15 | 29.03 | 29.19 | 29 | 29.3 |  |  |
| Q9R1P3 | Psmb2 | | 0.28 | | 29.12 | | 29.66 | 29.55 | 29.58 | 29.47 | 29.81 | 29.83 | 29.55 | 29.91 |  |  |
| P20491 | Fcer1g | | 0.58 | | 29.11 | | 28.55 | 26.34 | 28.2 | 27.13 | 28.46 | 28.96 | 29.2 | 28.05 |  |  |
| P51150 | Rab7a | | 0.89 | | 29.11 | | 29.36 | 28.89 | 29.24 | 28.95 | 29.46 | 29.34 | 29.36 | 28.86 |  |  |
| P09405 | Ncl | | 0.96 | | 29.1 | | 28.63 | 27.95 | 28.52 | 28.77 | 28.49 | 28.69 | 28.97 | 28.3 |  |  |
| F8VQJ3 | Lamc1 | | 0.73 | | 29.09 | | 28.88 | 29.38 | 28.92 | 28.59 | 29.26 | 31.54 | 28.75 | 28.47 |  |  |
| Q9CR26 | Vta1 | | 0.92 | | 29.08 | | 28.97 | 28.92 | 28.72 | 29.06 | 29.39 | 28.55 | 29.29 | 29.07 |  |  |
| Q9QUI0 | Rhoa | | 0.07 | | 29.07 | | 28.78 | 29.19 | 29.2 | 29.49 | 29.55 | 29.07 | 29.21 | 29.28 |  |  |
| P17426 | Ap2a1 | | 0.34 | | 29.06 | | 28.35 | 27.33 | 29.6 | 29.09 | 28.89 | 28.66 | 29.13 | 27.25 |  |  |
| Q8BP67 | Rpl24 | | 0.34 | | 29.06 | | 28.77 | 29.09 | 28.74 | 28.76 | 29.26 | 28.45 | 28.85 | 28.75 |  |  |
| D3Z0B9 | Aldh16a1 | | 0.72 | | 29.06 | | 29.3 | 29.08 | 28.74 | 29.32 | 29.15 | 29.29 | 29.36 | 29.01 |  |  |
| Q9DCH4 | Eif3f | | 0.48 | | 29.06 | | 29.69 | 27.72 | 29.12 | 29.8 | 29.14 | 29.73 | 29.65 | 29.06 |  |  |
| G5E8D6 | Efemp2 | | 0.97 | | 29.06 | | 28.82 | 29.43 | 29.38 | 28.64 | 29.37 | 29.04 | 28.98 | 29.21 |  |  |
| Q61001 | Lama5 | | 0.38 | | 29.05 | | 29.27 | 30.23 | 28.31 | 28.73 | 29.95 | 28.71 | 29.02 | 28.56 |  |  |
| P62267 | Rps23 | | 0.81 | | 29.05 | | 29.1 | 28.45 | 28.91 | 28.53 | 29.2 | 28.24 | 29.07 | 28.79 |  |  |
| P47880 | Igfbp6 | | 0.09 | | 29.05 | | 29.66 | 29.31 | 28.9 | 29.18 | 28.82 | 28.87 | 28.73 | 29.05 |  |  |
| Q9Z1P8 | Angptl4 | | 0.51 | | 29.04 | | 28.98 | 29.88 | 28.96 | 29.17 | 28.65 | 28.66 | 29 | 29.38 |  |  |
| Q9ET01 | Pygl | | 0.85 | | 29.01 | | 29.26 | 29.44 | 28.66 | 29.71 | 29.48 | 29 | 28.44 | 29.73 |  |  |
| Q8BG32 | Psmd11 | | 0.55 | | 28.99 | | 29.17 | 28.81 | 28.83 | 29.17 | 29.04 | 29.35 | 29.18 | 28.93 |  |  |
| Q91WK2 | Eif3h | | 0.3 | | 28.97 | | 29.16 | 27.59 | 29.13 | 29.76 | 28.85 | 29.72 | 29.36 | 28.98 |  |  |
| Q60737 | Csnk2a1 | | 0.51 | | 28.96 | | 29.12 | 28.74 | 29.15 | 28.45 | 28.86 | 28.65 | 28.86 | 28.56 |  |  |
| O09164 | Sod3 | | 0.76 | | 28.96 | | 28.84 | 30.37 | 28.53 | 27.97 | 30.18 | 29.14 | 29.29 | 29.27 |  |  |
| B2M1R6 | Hnrnpk | | 0.81 | | 28.94 | | 28.83 | 28.38 | 28.9 | 29.04 | 28.38 | 28.66 | 29.05 | 27.96 |  |  |
| Q99PV0 | Prpf8 | | 0.35 | | 28.91 | | 28.32 | 28.06 | 28.48 | 28.21 | 28.86 | 28.94 | 29.13 | 28.55 |  |  |
| Q9JII6 | Akr1a1 | | 0.66 | | 28.9 | | 26.88 | 29.09 | 27.43 | 28.33 | 27.07 | 28.73 | 26.65 | 27.53 |  |  |
| P34884 | Mif | | 0.84 | | 28.89 | | 29 | 29.2 | 29.01 | 29.25 | 28.95 | 29.05 | 28.87 | 29.5 |  |  |
| Q3U5Q7 | Cmpk2 | | 0.63 | | 28.88 | | 28.84 | 26.69 | 28.69 | 29.53 | 28.24 | 29.41 | 29.29 | 27.8 |  |  |
| P16254 | Srp14 | | 0.39 | | 28.88 | | 28.47 | 27.45 | 28.64 | 28.65 | 29.27 | 28.87 | 28.99 | 28.38 |  |  |
| Q9EQH3 | Vps35 | | 0.18 | | 28.88 | | 28.77 | 28.41 | 28.94 | 29.66 | 28.69 | 29.11 | 29.72 | 29.16 |  |  |
| P32067 | Ssb | | 0.7 | | 28.87 | | 27.97 | 28.26 | 28.36 | 28.37 | 28.61 | 28.38 | 28.35 | 27.95 |  |  |
| P09055 | Itgb1 | | 0.78 | | 28.86 | | 28.73 | 28.06 | 28.75 | 28.25 | 28.72 | 28.55 | 28.9 | 27.41 |  |  |
| O70194 | Eif3d | | 0.08 | | 28.86 | | 29.25 | 28.43 | 29.78 | 29.79 | 29.48 | 29.53 | 29.33 | 28.69 |  |  |
| Q8BYI9 | Tnr | | 0.2 | | 28.86 | | 29.27 | 29 | 28.22 | 28.3 | 29.13 | 28.5 | 28.44 | 28.8 |  |  |
| Q61508 | Ecm1 | | 0.46 | | 28.86 | | 28.36 | 29.55 | 28.47 | 28.57 | 29.22 | 28.12 | 28.18 | 28.92 |  |  |
| Q91VI7 | Rnh1 | | 0.96 | | 28.85 | | 28.53 | 27.68 | 28.63 | 29.12 | 27.76 | 29.15 | 28.98 | 27.43 |  |  |
| Q8C2Q7 | Hnrnph1 | | 0.96 | | 28.85 | | 28.79 | 27.78 | 28.58 | 28.59 | 28.38 | 28.41 | 28.84 | 28.43 |  |  |
| O88398 | Avil | | 0.55 | | 28.85 | | 28.53 | 27.9 | 28.1 | 28.4 | 28.77 | 28.68 | 28.75 | 28.69 |  |  |
| Q9CZX8 | Rps19 | | 0.97 | | 28.84 | | 28.56 | 27.3 | 28.4 | 29.11 | 27.42 | 28.79 | 28.72 | 26.83 |  |  |
| P60335 | Pcbp1 | | 0.85 | | 28.81 | | 28.3 | 28.4 | 28.57 | 29.16 | 28.5 | 28.98 | 29.13 | 27.71 |  |  |
| P62960 | Ybx1 | | 0.74 | | 28.8 | | 28.93 | 27.82 | 27.54 | 28.84 | 28.4 | 28.25 | 28.75 | 27.23 |  |  |
| P04918 | Saa3 | | 0.08 | | 28.8 | | 29.29 | 28.78 | 29.21 | 30.34 | 29.29 | 28.42 | 28.98 | 28.43 |  |  |
| O55029 | Copb2 | | 0.17 | | 28.8 | | 28.9 | 28.32 | 29.1 | 29.31 | 28.88 | 29.24 | 29.15 | 28.78 |  |  |
| Q99JY9 | Actr3 | | 0.82 | | 28.8 | | 28.8 | 29.09 | 28.74 | 28.99 | 29.11 | 29.13 | 29.02 | 28.8 |  |  |
| Q9WVJ2 | Psmd13 | | 0.62 | | 28.79 | | 29.45 | 29.19 | 28.82 | 29.16 | 28.9 | 29.3 | 29.03 | 29.01 |  |  |
| P63276 | Rps17 | | 0.79 | | 28.78 | | 28.65 | 28.63 | 28.71 | 29.04 | 27.81 | 28.96 | 28.6 | 27.52 |  |  |
| Q9CQV8 | Ywhab | | 0.93 | | 28.78 | | 28.68 | 29.23 | 28.45 | 29.06 | 29.54 | 29.28 | 29.22 | 28.52 |  |  |
| P11438 | Lamp1 | | 0.9 | | 28.77 | | 27.92 | 28.37 | 28.76 | 28.37 | 28.41 | 28.8 | 28.76 | 27.49 |  |  |
| D3YV69 | Rab6a | | 0.51 | | 28.77 | | 27.95 | 28.84 | 28.21 | 28.42 | 28.46 | 28.16 | 28.4 | 28.07 |  |  |
| E9Q1Y3 | Apob | | 0.06 | | 28.75 | | 29 | 29.13 | 28.48 | 28.87 | 29.01 | 29.25 | 29.42 | 29.2 |  |  |
| B1AWE0 | Clta | | 0.51 | | 28.74 | | 27.95 | 27.81 | 28.53 | 28.53 | 28.19 | 27.71 | 28.83 | 26.58 |  |  |
| P62334 | Psmc6 | | 0.94 | | 28.74 | | 28.7 | 28.55 | 28.33 | 29.14 | 28.16 | 29 | 28.74 | 28.03 |  |  |
| Q8QZY6 | Tspan14 | | 0.31 | | 28.74 | | 28.9 | 29.42 | 28.25 | 28.5 | 29.07 | 28.5 | 28.36 | 28.89 |  |  |
| P54775 | Psmc4 | | 0.38 | | 28.74 | | 29.12 | 28.82 | 28.87 | 29.16 | 29.15 | 29.02 | 29.09 | 29.02 |  |  |
| Q9D1R9 | Rpl34 | | 0.61 | | 28.73 | | 28.7 | 27.76 | 28.42 | 28.32 | 29.03 | 28.3 | 28.34 | 28.15 |  |  |
| Q8K1B8 | Fermt3 | | 0.54 | | 28.73 | | 28.99 | 28.5 | 28.9 | 27.21 | 28.59 | 28.49 | 28.43 | 29.07 |  |  |
| P14685 | Psmd3 | | 0.7 | | 28.72 | | 28.94 | 28.73 | 28.68 | 29.32 | 28.79 | 28.91 | 28.92 | 28.95 |  |  |
| Q9DB34 | Chmp2a | | 0.54 | | 28.71 | | 28.87 | 28.63 | 28.42 | 28.22 | 29.19 | 28.61 | 28.92 | 29.34 |  |  |
| A0A1W2P6F6 | Myl6 | | 0.61 | | 28.7 | | 29.12 | 28.99 | 28.71 | 29.11 | 28.13 | 28.93 | 28.99 | 28.54 |  |  |
| P16460 | Ass1 | | 0.22 | | 28.7 | | 28.18 | 28.5 | 28.78 | 28.86 | 28.48 | 29.07 | 28.5 | 29.2 |  |  |
| Q8K1K2 | Psmc5 | | 0.68 | | 28.69 | | 28.94 | 28.35 | 28.4 | 28.87 | 28.05 | 28.57 | 28.63 | 28.38 |  |  |
| Q9D0I9 | Rars | | 0.46 | | 28.69 | | 29.23 | 28.51 | 28.99 | 29.17 | 28.66 | 29.39 | 29.42 | 28.73 |  |  |
| Q3TVK3 | Dnpep | | 0.94 | | 28.68 | | 28.98 | 29.72 | 28.82 | 29.45 | 29.27 | 29.44 | 29.18 | 29.08 |  |  |
| Q3TWV4 | Ap2m1 | | 0.77 | | 28.67 | | 29.16 | 27.97 | 28.65 | 29.08 | 29.06 | 28.81 | 29.34 | 27.97 |  |  |
| Q62093 | Srsf2 | | 0.55 | | 28.67 | | 28.76 | 26.27 | 28.92 | 29.15 | 27.35 | 29.47 | 29 | 28.19 |  |  |
| Q8R1Q8 | Dync1li1 | | 0.63 | | 28.65 | | 29.02 | 28.2 | 28.33 | 29.12 | 27.73 | 28.88 | 28.68 | 28.75 |  |  |
| Q9CZD3 | Gars | | 0.93 | | 28.65 | | 29.09 | 29.26 | 28.74 | 29.14 | 29.06 | 29.18 | 29.06 | 28.92 |  |  |
| P70195 | Psmb7 | | 0.94 | | 28.65 | | 29.01 | 29.38 | 28.65 | 29.41 | 29.02 | 29.18 | 28.93 | 29.17 |  |  |
| Q9WTM5 | Ruvbl2 | | 0.18 | | 28.64 | | 29.02 | 28.2 | 28.58 | 29.03 | 28.55 | 29.22 | 29.12 | 28.97 |  |  |
| Q61233 | Lcp1 | | 0.6 | | 28.63 | | 28.14 | 25.64 | 28.5 | 28.75 | 27.2 | 28.83 | 28.85 | 27.58 |  |  |
| Q9D2G2 | Dlst | | 0.38 | | 28.63 | | 28.35 | 26.82 | 28.09 | 27.31 | 28.56 | 29.11 | 28.3 | 28.76 |  |  |
| P61982 | Ywhag | | 0.46 | | 28.63 | | 28.75 | 27.95 | 28.56 | 28.34 | 28.11 | 28.64 | 28.43 | 28.94 |  |  |
| E9PZC3 | Blvrb | | 0.68 | | 28.63 | | 28.89 | 29.19 | 28.7 | 29.06 | 28.87 | 28.69 | 28.59 | 28.97 |  |  |
| O55234 | Psmb5 | | 0.56 | | 28.63 | | 29.27 | 29.68 | 28.34 | 29.12 | 29.02 | 29.03 | 28.88 | 29.54 |  |  |
| P43274 | Hist1h1e | | 0.94 | | 28.62 | | 28.41 | 26.81 | 27.88 | 29.39 | 27.02 | 28.74 | 28.92 | 27.12 |  |  |
| Q62167 | Ddx3x | | 0.46 | | 28.61 | | 28.58 | 28.13 | 28.41 | 28.11 | 28.11 | 28.51 | 28.15 | 28.04 |  |  |
| Q64339 | Isg15 | | 0.25 | | 28.6 | | 28.45 | 27.92 | 27.6 | 27.65 | 27.52 | 28.55 | 28.73 | 27.25 |  |  |
| Q3TWW8 | Srsf6 | | 0.88 | | 28.6 | | 28.72 | 27.58 | 28.05 | 29.08 | 27.65 | 29.29 | 28.66 | 27.64 |  |  |
| Q64337 | Sqstm1 | | 0.69 | | 28.6 | | 28.64 | 27.71 | 28.25 | 28.43 | 28.21 | 28.9 | 28.98 | 27.91 |  |  |
| A2AL12 | Hnrnpa3 | | 0.73 | | 28.59 | | 28.2 | 27.02 | 27.86 | 28.07 | 27.91 | 28.38 | 28.18 | 25.44 |  |  |
| J9VV68 |  | | 0.83 | | 28.59 | | 28.65 | 27.13 | 28.81 | 28.33 | 26.89 | 27.19 | 28.51 | 27.4 |  |  |
| O89053 | Coro1a | | 0.1 | | 28.59 | | 27.36 | 28.16 | 28.98 | 29.18 | 28.74 | 28.58 | 28.32 | 27.68 |  |  |
| Q3UDE2 | Ttll12 | | 0.48 | | 28.58 | | 27.45 | 24.69 | 28.2 | 26.83 | 28.49 | 28.93 | 28.55 | 27.43 |  |  |
| Q921R2 | Rps13 | | 0.81 | | 28.58 | | 28.43 | 26.99 | 28.53 | 28.71 | 27.73 | 28.69 | 28.42 | 27.7 |  |  |
| Q91YP3 | Dera | | 0.86 | | 28.58 | | 28.7 | 28.1 | 28.46 | 28.23 | 28.3 | 28.95 | 28.88 | 27.73 |  |  |
| Q99JI4 | Psmd6 | | 0.48 | | 28.58 | | 28.55 | 28.15 | 29.22 | 28.51 | 28.61 | 28.81 | 28.9 | 28.22 |  |  |
| F6SVV1 | Gm9493 | | 0.45 | | 28.58 | | 28.14 | 28.25 | 28.26 | 28.73 | 28.45 | 29.07 | 28.63 | 28.26 |  |  |
| O08688 | Capn5 | | 0.75 | | 28.58 | | 27.99 | 28.34 | 28.84 | 27.82 | 28.6 | 28.04 | 28.16 | 28.38 |  |  |
| Q99L45 | Eif2s2 | | 0.14 | | 28.57 | | 26.06 | 29.73 | 30.95 | 29.86 | 29.69 | 24.54 | 28.69 | 17.5 |  |  |
| O55142 | Rpl35a | | 0.63 | | 28.57 | | 28.85 | 27.58 | 28.35 | 27.74 | 27.93 | 27.97 | 28.12 | 28.11 |  |  |
| Q61598 | Gdi2 | | 0.29 | | 28.57 | | 28.84 | 28.65 | 28.76 | 28.98 | 28.57 | 28.95 | 28.89 | 28.84 |  |  |
| Q9QXS1 | Plec | | 0.25 | | 28.56 | | 27.83 | 28.4 | 28.5 | 28.6 | 28.82 | 28.39 | 28.9 | 28.63 |  |  |
| P60867 | Rps20 | | 0.9 | | 28.55 | | 28.5 | 27.81 | 28.65 | 28.93 | 27.65 | 28.51 | 28.4 | 27.75 |  |  |
| Q60692 | Psmb6 | | 0.87 | | 28.54 | | 29.29 | 29.57 | 29.16 | 29.5 | 29.07 | 29.13 | 28.79 | 29.34 |  |  |
| P62245 | Rps15a | | 0.46 | | 28.53 | | 28.07 | 27.42 | 28.39 | 28.63 | 28.1 | 28.62 | 28.38 | 28.11 |  |  |
| P98063 | Bmp1 | | 0.45 | | 28.52 | | 29.38 | 29.2 | 28.85 | 28.8 | 29.24 | 29.47 | 29.22 | 29.17 |  |  |
| P12804 | Fgl2 | | 0.28 | | 28.52 | | 29.94 | 31.08 | 29 | 28.26 | 29.67 | 29.91 | 30.04 | 30.7 |  |  |
| P28063 | Psmb8 | | 0.4 | | 28.51 | | 28.29 | 28.54 | 28.93 | 28.74 | 28.57 | 28.77 | 28.23 | 29.02 |  |  |
| Q9ESU7 | Slc1a5 | | 0.71 | | 28.5 | | 28.02 | 27.57 | 28.49 | 27.61 | 28.75 | 28.34 | 28.21 | 28.37 |  |  |
| Q9QZE5 | Copg1 | | 0.14 | | 28.49 | | 28.7 | 27.7 | 28.95 | 28.91 | 28.75 | 28.71 | 28.74 | 28.67 |  |  |
| Q9CWJ9 | Atic | | 0.74 | | 28.48 | | 29.47 | 28.19 | 28.98 | 29.26 | 28.8 | 28.99 | 28.42 | 29.3 |  |  |
| O08710 | Tg | | 0.29 | | 28.46 | | 30.77 | 29.63 | 29.02 | 31.29 | 29.32 | 30.78 | 30.56 | 31.58 |  |  |
| P45376 | Akr1b1 | | 0.28 | | 28.45 | | 28.78 | 28.69 | 28.35 | 28.89 | 28.11 | 28.39 | 28.21 | 28.21 |  |  |
| Q9DCD0 | Pgd | | 0.19 | | 28.45 | | 28.34 | 28.67 | 28.5 | 28.7 | 28.47 | 28.72 | 28.59 | 28.85 |  |  |
| E9QB02 | Mars | | 0.39 | | 28.45 | | 29.41 | 28.11 | 29.12 | 28.8 | 29.09 | 29.51 | 29.22 | 28.85 |  |  |
| J9VM28 |  | | 0.77 | | 28.44 | | 28.35 | 27.2 | 27.57 | 28.06 | 27.58 | 28.08 | 27.89 | 27.73 |  |  |
| P09103 | P4hb | | 0.05 | | 28.44 | | 28.27 | 28.45 | 27.9 | 28.35 | 28.4 | 28.85 | 28.49 | 28.88 |  |  |
| P51410 | Rpl9 | | 0.91 | | 28.43 | | 28.25 | 28.09 | 28.16 | 28.65 | 28.24 | 28.37 | 27.94 | 28.72 |  |  |
| A0A2R8VHN3 | Pcbp2 | | 0.73 | | 28.42 | | 27.12 | 27.86 | 27.75 | 27.86 | 27.85 | 28.24 | 28.4 | 27.58 |  |  |
| B7ZCF1 | Psmc3 | | 0.87 | | 28.42 | | 28.61 | 28.55 | 28.83 | 28.63 | 28.06 | 28.46 | 28.35 | 28.47 |  |  |
| Q5XJF6 | Rpl10a | | 0.99 | | 28.42 | | 28.63 | 28.66 | 28.39 | 28.74 | 28.62 | 28.55 | 28.42 | 28.75 |  |  |
| Q8BP47 | Nars | | 0.91 | | 28.41 | | 28.03 | 28.08 | 28.02 | 28.46 | 28.05 | 28.22 | 28.52 | 27.46 |  |  |
| Q9CQD4 | Chmp1b2 | | 0.81 | | 28.41 | | 28.72 | 27.91 | 28.26 | 28.33 | 28.99 | 28.27 | 28.49 | 28.71 |  |  |
| P01899 | H2-D1 | | 0.75 | | 28.39 | | 27.79 | 25.19 | 27.51 | 27.89 | 27.61 | 28.01 | 28.28 | 26.93 |  |  |
| Q9D1C8 | Vps28 | | 0.63 | | 28.39 | | 28.15 | 28 | 28.31 | 27.73 | 27.95 | 27.97 | 28.25 | 27.97 |  |  |
| Q64514 | Tpp2 | | 0.96 | | 28.39 | | 28.58 | 27.23 | 27.67 | 28.15 | 28.75 | 28.32 | 27.86 | 28.34 |  |  |
| P51863 | Atp6v0d1 | | 0.93 | | 28.38 | | 27.38 | 28.16 | 27.9 | 26.88 | 28.68 | 28.21 | 28.27 | 26.68 |  |  |
| E9Q1W0 | Camk2d | | 0.41 | | 28.38 | | 28.31 | 27.93 | 27.9 | 28.15 | 27.76 | 28.3 | 28.46 | 27.84 |  |  |
| P62874 | Gnb1 | | 0.82 | | 28.36 | | 26.97 | 28.29 | 28.25 | 25.39 | 28.36 | 28.27 | 28.44 | 26.92 |  |  |
| Q5SS83 | Flot2 | | 0.64 | | 28.36 | | 28.19 | 27.58 | 28 | 27.92 | 27.87 | 28.08 | 28.27 | 28.04 |  |  |
| G5E866 | Sf3b1 | | 0.1 | | 28.35 | | 28.19 | 28.15 | 28.18 | 28.1 | 28.07 | 27.96 | 28.11 | 27.64 |  |  |
| P26516 | Psmd7 | | 0.97 | | 28.33 | | 28.45 | 28 | 27.62 | 28.63 | 28.33 | 28.6 | 28.46 | 27.75 |  |  |
| Q9D379 | Ephx1 | | 0.67 | | 28.33 | | 28.53 | 26.92 | 28.07 | 28.11 | 28.91 | 28.01 | 28.25 | 27.93 |  |  |
| Q9CYN9 | Atp6ap2 | | 0.97 | | 28.33 | | 29.13 | 29.08 | 28.65 | 28.78 | 29.16 | 28.71 | 28.54 | 29.49 |  |  |
| Q9JLJ2 | Aldh9a1 | | 0.39 | | 28.31 | | 28.56 | 28 | 27.77 | 28.47 | 27.79 | 28.36 | 28.12 | 28.76 |  |  |
| H7BX95 | Srsf1 | | 0.77 | | 28.3 | | 27.43 | 26 | 27.69 | 29.04 | 26.8 | 29.53 | 28.15 | 26.31 |  |  |
| A0A0R4J119 | Cyfip1 | | 0.43 | | 28.3 | | 28.29 | 27.19 | 28.4 | 28.67 | 28.5 | 28.43 | 28.85 | 27.57 |  |  |
| A2AAA9 | Mrc2 | | 0.99 | | 28.3 | | 27.44 | 29.14 | 28.12 | 28.37 | 28.35 | 28.52 | 28.33 | 27.82 |  |  |
| A0A087WQW8 | Fn1 | | 0.75 | | 28.3 | | 27.27 | 29 | 27.42 | 27.51 | 28.73 | 28.38 | 28.51 | 28 |  |  |
| Q8C266 | Rab5c | | 0.24 | | 28.3 | | 27.91 | 27.35 | 28.09 | 28.4 | 28.27 | 28.44 | 28.16 | 28.21 |  |  |
| D3YZ61 | C1qtnf3 | | 0.88 | | 28.29 | | 28.63 | 28.33 | 28.43 | 26.81 | 29.15 | 28.1 | 27.64 | 29.75 |  |  |
| S4R1M0 | Ptprc | | 0.36 | | 28.27 | | 25.27 | 23.26 | 27.46 | 27.24 | 27.26 | 28.1 | 28.57 | 25.96 |  |  |
| P50543 | S100a11 | | 0.18 | | 28.26 | | 28.07 | 28.05 | 26.75 | 26.37 | 27.39 | 28.49 | 28.2 | 26.32 |  |  |
| P25206 | Mcm3 | | 0.26 | | 28.25 | | 24.59 | 29.55 | 30.62 | 24.47 | 28.97 | 24.97 | 24.84 | 24.3 |  |  |
| Q8K2Q7 | Brox | | 0.81 | | 28.22 | | 28.39 | 28.13 | 27.86 | 27.76 | 28.63 | 27.95 | 28.13 | 28.31 |  |  |
| Q91Z25 | Arpc1b | | 0.9 | | 28.21 | | 28.22 | 28.23 | 28.2 | 28.39 | 27.82 | 28.37 | 28.29 | 27.82 |  |  |
| P14426 | H2-D1 | | 0.38 | | 28.21 | | 28.4 | 29.14 | 27.88 | 28.92 | 28.19 | 28.05 | 27.87 | 28.23 |  |  |
| E9QN37 | Mpeg1 | | 0.05 | | 28.2 | | 27.87 | 27.75 | 26.96 | 27.5 | 27.22 | 28.63 | 28.24 | 27.57 |  |  |
| P50608 | Fmod | | 0.11 | | 28.2 | | 28.52 | 28.74 | 28.96 | 28.91 | 27.77 | 27 | 26.97 | 28.28 |  |  |
| A0A0R4J0S2 | Igfals | | 0.98 | | 28.2 | | 28.33 | 29 | 28.91 | 28.55 | 28.04 | 28.2 | 27.73 | 29.34 |  |  |
| P16125 | Ldhb | | 0.84 | | 28.2 | | 29.42 | 29.27 | 29.02 | 29.29 | 29.22 | 29.05 | 28.66 | 29.85 |  |  |
| A0A2C9F2D2 | Anxa7 | | 0.39 | | 28.19 | | 28.14 | 27.4 | 28.31 | 28.28 | 28.76 | 28.36 | 28.57 | 27.5 |  |  |
| P62814 | Atp6v1b2 | | 0.94 | | 28.18 | | 28.18 | 27.75 | 28.16 | 28.52 | 27.78 | 28.6 | 28.54 | 27.35 |  |  |
| O89051 | Itm2b | | 0.15 | | 28.18 | | 27.53 | 27.73 | 27.19 | 27.34 | 27.68 | 27.49 | 27.35 | 27.46 |  |  |
| Q61024 | Asns | | 0.26 | | 28.18 | | 28.53 | 28.8 | 28.5 | 28.92 | 28.7 | 29.4 | 28.71 | 28.78 |  |  |
| P28271 | Aco1 | | 0.76 | | 28.18 | | 29.07 | 28.92 | 28.87 | 29.01 | 28.86 | 28.79 | 28.46 | 29.05 |  |  |
| Q60710 | Samhd1 | | 0.29 | | 28.16 | | 28.12 | 27.31 | 29.11 | 28.61 | 27.97 | 28.35 | 28.24 | 27.48 |  |  |
| P46471 | Psmc2 | | 0.12 | | 28.16 | | 28.28 | 27.24 | 27.75 | 28.51 | 27.99 | 28.74 | 28.89 | 28.46 |  |  |
| E9QN70 | Lamb1 | | 0.95 | | 28.15 | | 28.38 | 29.38 | 28.3 | 28.4 | 29.57 | 29.04 | 28.61 | 28.7 |  |  |
| P97384 | Anxa11 | | 0.95 | | 28.14 | | 28.12 | 27.45 | 28.1 | 26.78 | 28.81 | 27.73 | 28.36 | 28.08 |  |  |
| P40237 | Cd82 | | 0.44 | | 28.13 | | 27.67 | 26.89 | 27.65 | 28.22 | 28.4 | 27.95 | 27.13 | 27.88 |  |  |
| O88200 | Clec11a | | 0.11 | | 28.08 | | 27.94 | 28.51 | 28.64 | 28.62 | 28.54 | 28.37 | 28.2 | 28.55 |  |  |
| O54890 | Itgb3 | | 0.94 | | 28.07 | | 27.51 | 27.44 | 27.55 | 27.64 | 27.87 | 27.64 | 27.94 | 27.26 |  |  |
| O35375 | Nrp2 | | 0.98 | | 28.07 | | 27.53 | 27.66 | 27.68 | 27.61 | 27.96 | 27.88 | 27.93 | 27.32 |  |  |
| P70168 | Kpnb1 | | 0.41 | | 28.07 | | 28.19 | 27.9 | 27.92 | 28.43 | 28.5 | 28.16 | 28.15 | 28.09 |  |  |
| Q8CFE6 | Slc38a2 | | 0.63 | | 28.06 | | 28.07 | 27.58 | 27.52 | 27.7 | 27.46 | 27.89 | 28.36 | 27.04 |  |  |
| P62192 | Psmc1 | | 0.31 | | 28.05 | | 28.32 | 27.81 | 27.76 | 28.04 | 28.25 | 28.49 | 28.56 | 28.02 |  |  |
| Q9D7S9 | Chmp5 | | 0.43 | | 28.05 | | 27.85 | 26.99 | 27.48 | 27.78 | 28.02 | 27.88 | 28.14 | 28.1 |  |  |
| Q91ZJ5 | Ugp2 | | 0.93 | | 28.05 | | 28.93 | 29.09 | 28.8 | 28.47 | 28.62 | 28.87 | 28.32 | 29.05 |  |  |
| A0A0N4SV66 | Hist1h2ah | | 0.92 | | 28.05 | | 30.55 | 28.47 | 30.17 | 28.04 | 27.64 | 27.97 | 28.07 | 31.18 |  |  |
| P17047 | Lamp2 | | 0.5 | | 28.03 | | 27.18 | 26.78 | 27.71 | 27.57 | 27.57 | 28.47 | 27.91 | 27.21 |  |  |
| Q9Z1R3 | Apom | | 0.15 | | 28.03 | | 27.47 | 27.45 | 28.8 | 27.94 | 28.49 | 28.28 | 27.99 | 27.38 |  |  |
| P49817 | Cav1 | | 0.41 | | 28.03 | | 27.02 | 27.1 | 27 | 26.97 | 27.98 | 27.59 | 28.08 | 27.85 |  |  |
| Q8VCJ6 | Mrgprf | | 0.49 | | 28.02 | | 27.56 | 28.22 | 28.19 | 27.44 | 28.57 | 27.76 | 27.23 | 27.89 |  |  |
| P99026 | Psmb4 | | 0.13 | | 28.02 | | 29.06 | 28.7 | 29.01 | 28.56 | 28.46 | 29.47 | 28.93 | 29.49 |  |  |
| A0A0R4J1Q0 | Edc4 | | 0.95 | | 28.01 | | 26.59 | 27.99 | 27.41 | 26.67 | 28.16 | 26.89 | 28.25 | 26.82 |  |  |
| E9PZF0 | Gm20390 | | 0.1 | | 28.01 | | 28.21 | 27.64 | 27.57 | 27.31 | 27.79 | 28.25 | 27.97 | 27.91 |  |  |
| Q61171 | Prdx2 | | 0.75 | | 28.01 | | 27.6 | 26.25 | 26.87 | 27.31 | 26.22 | 26.56 | 26.75 | 27.95 |  |  |
| Q3U3V1 | F10 | | 0.2 | | 28.01 | | 28.09 | 28.18 | 28.36 | 28.43 | 28.22 | 28.51 | 28.1 | 28.93 |  |  |
| P62830 | Rpl23 | | 0.27 | | 28 | | 27.48 | 28.01 | 27.85 | 28.28 | 27.77 | 27.07 | 28.12 | 26.45 |  |  |
| Q91XL3 | Uxs1 | | 0.34 | | 28 | | 28.56 | 29.09 | 27.64 | 28.33 | 28.14 | 27.97 | 28.39 | 28.3 |  |  |
| P63037 | Dnaja1 | | 0.56 | | 28 | | 28.38 | 27.74 | 28.34 | 28.54 | 28.17 | 27.62 | 28.19 | 28.57 |  |  |
| P19324 | Serpinh1 | | 0.98 | | 28 | | 27.39 | 27.54 | 27.19 | 27.81 | 27.68 | 27.02 | 26.98 | 28.72 |  |  |
| Q9D1D4 | Tmed10 | | 0.5 | | 28 | | 27.5 | 27.61 | 27.68 | 27.03 | 28.24 | 27.4 | 28.1 | 29.33 |  |  |
| Q02105 | C1qc | | 0.62 | | 27.97 | | 29.32 | 28.26 | 28.33 | 27.63 | 28.46 | 28.35 | 28.16 | 29.33 |  |  |
| E9Q604 | Itgam | | 0.18 | | 27.96 | | 25.82 | 26.86 | 28.89 | 29.05 | 27.83 | 29.58 | 29.46 | 26.88 |  |  |
| P84104 | Srsf3 | | 0.61 | | 27.95 | | 27.65 | 26.94 | 27.55 | 27.08 | 27.21 | 27.93 | 26.61 | 26.58 |  |  |
| P67871 | Csnk2b | | 0.76 | | 27.94 | | 27.06 | 27.29 | 26.99 | 27.45 | 26.94 | 27.61 | 27.62 | 26 |  |  |
| O70475 | Ugdh | | 0.44 | | 27.94 | | 28.14 | 30.31 | 26.42 | 28.7 | 27.86 | 28.3 | 28.21 | 28.48 |  |  |
| Q9D1A2 | Cndp2 | | 0.93 | | 27.92 | | 28.1 | 28.38 | 28.05 | 28.62 | 27.5 | 27.88 | 28.46 | 27.68 |  |  |
| Q99KP6 | Prpf19 | | 0.99 | | 27.91 | | 27.59 | 27.2 | 27.37 | 27.39 | 27.83 | 27.66 | 27.96 | 27.02 |  |  |
| Q76MZ3 | Ppp2r1a | | 0.55 | | 27.91 | | 28.43 | 27.94 | 27.09 | 28.26 | 27.86 | 27.94 | 27.99 | 28.03 |  |  |
| Q9JI48 | Plac8 | | 0.24 | | 27.91 | | 27.64 | 27.36 | 27.58 | 28.03 | 28.8 | 28.25 | 28.1 | 28.26 |  |  |
| Q9QYB1 | Clic4 | | 0.88 | | 27.89 | | 28.09 | 27.4 | 27.81 | 27.88 | 27.54 | 28.03 | 27.85 | 27.03 |  |  |
| Q9CZN7 | Shmt2 | | 0.49 | | 27.89 | | 27.93 | 27.91 | 28.07 | 28.24 | 27.5 | 27.85 | 27.79 | 27.43 |  |  |
| E9PYL9 | Gm10036 | | 0.58 | | 27.88 | | 28.56 | 28.31 | 28.2 | 28.58 | 27.89 | 27.67 | 28.13 | 28.17 |  |  |
| H3BKH6 | Esd | | 0.59 | | 27.87 | | 27.85 | 27.82 | 28.01 | 29.26 | 27.56 | 28.57 | 27.86 | 28.31 |  |  |
| Q80T21 | Adamtsl4 | | 0.56 | | 27.86 | | 28.1 | 27.1 | 27.95 | 27.86 | 27.37 | 27.91 | 27.57 | 26.02 |  |  |
| P53994 | Rab2a | | 0.35 | | 27.86 | | 26.78 | 26.33 | 28.07 | 27.26 | 27.74 | 27.57 | 27.56 | 27.03 |  |  |
| Q00PI9 | Hnrnpul2 | | 0.54 | | 27.85 | | 25.32 | 26.81 | 27.04 | 27.41 | 24.7 | 26.25 | 24.93 | 25.54 |  |  |
| P63325 | Rps10 | | 0.79 | | 27.85 | | 27.49 | 26.24 | 27.69 | 27.41 | 26.98 | 27.7 | 27.47 | 27.33 |  |  |
| Q9CZU6 | Cs;Csl | | 0.37 | | 27.85 | | 27.87 | 28.09 | 27.82 | 28.24 | 27.95 | 28.39 | 27.98 | 28.13 |  |  |
| A0A1L1SV25 | Actn4 | | 0.78 | | 27.84 | | 27.64 | 27.8 | 27.58 | 28.19 | 27.8 | 27.66 | 27.83 | 28.25 |  |  |
| P27046 | Man2a1 | | 0.58 | | 27.84 | | 27.95 | 27.9 | 27.99 | 27.43 | 28.25 | 27.94 | 27.94 | 28.54 |  |  |
| Q8VI94 | Oasl1 | | 0.14 | | 27.83 | | 27.67 | 27.76 | 27.38 | 26.09 | 27.23 | 26.46 | 27.35 | 27.14 |  |  |
| Q3TW96 | Uap1l1 | | 0.8 | | 27.83 | | 28.02 | 27.91 | 27.86 | 27.97 | 28 | 28.12 | 27.65 | 27.81 |  |  |
| Q9WUU7 | Ctsz | | 0.46 | | 27.83 | | 27.81 | 28.84 | 28.02 | 28.69 | 28.71 | 27.86 | 27.77 | 28.36 |  |  |
| Q62465 | Vat1 | | 0.12 | | 27.82 | | 27.81 | 27.46 | 27.21 | 27.67 | 27.41 | 27.27 | 27.17 | 27.45 |  |  |
| P14069 | S100a6 | | 0.19 | | 27.82 | | 27.78 | 27.39 | 28.21 | 27.24 | 28.44 | 26.84 | 27.31 | 27.48 |  |  |
| P13020 | Gsn | | 0.26 | | 27.81 | | 27.42 | 28.59 | 27.76 | 27.48 | 28.32 | 24.98 | 28.05 | 26.84 |  |  |
| Q8CAG6 | Plek | | 0.66 | | 27.79 | | 28.07 | 26.3 | 27.43 | 28.08 | 27.39 | 28.01 | 27.64 | 24.35 |  |  |
| O55222 | Ilk | | 0.3 | | 27.79 | | 27.14 | 27.96 | 27.21 | 27.8 | 27.5 | 27.95 | 28 | 27.83 |  |  |
| D3Z7C6 | Ptges3 | | 0.37 | | 27.79 | | 28.72 | 28.3 | 28.94 | 28.26 | 27.69 | 28.89 | 28.89 | 28.58 |  |  |
| Q542I8 | Itgb2 | | 0.3 | | 27.78 | | 27.84 | 27.15 | 28.47 | 29.48 | 28.04 | 29.51 | 29.61 | 27.18 |  |  |
| Q6GT24 | Prdx6 | | 0.23 | | 27.77 | | 27.54 | 27.47 | 27.63 | 27.99 | 27.4 | 26.92 | 27.6 | 27.27 |  |  |
| Q3T9X3 | Dnm2 | | 0.24 | | 27.77 | | 26.94 | 27.13 | 26.9 | 27.09 | 27.21 | 28.21 | 27.5 | 27.29 |  |  |
| P68369 | Tuba1a | | 0.53 | | 27.76 | | 26.8 | 27.51 | 27.83 | 27.99 | 27.46 | 27.89 | 27.68 | 27.03 |  |  |
| O88569 | Hnrnpa2b1 | | 0.77 | | 27.74 | | 26.28 | 25.68 | 26.43 | 27.33 | 26.3 | 26.15 | 26.76 | 25.87 |  |  |
| P61161 | Actr2 | | 0.47 | | 27.74 | | 28.66 | 28.7 | 27.94 | 28.4 | 27.56 | 28.15 | 28.11 | 27.75 |  |  |
| Q9JIK5 | Ddx21 | | 0.74 | | 27.73 | | 27.58 | 25.69 | 27.07 | 26.76 | 25.53 | 26.66 | 27.18 | 26.08 |  |  |
| O08917 | Flot1 | | 0.41 | | 27.73 | | 27.72 | 27.78 | 27.6 | 27.1 | 27.89 | 27.91 | 27.81 | 27.67 |  |  |
| Q60841 | Reln | | 0.52 | | 27.7 | | 23.2 | 28.46 | 25.89 | 25.99 | 17.5 | 23.12 | 24.14 | 26.05 |  |  |
| Q7TPC1 | Cdsn | | 0.77 | | 27.69 | | 28.52 | 28.18 | 28.42 | 27.92 | 28.26 | 30.14 | 27.94 | 27.68 |  |  |
| E9Q8S8 | Hk3 | | 0.79 | | 27.68 | | 26.74 | 25.11 | 27.35 | 27.45 | 26.2 | 27.69 | 27.96 | 25.65 |  |  |
| A0A087WPL5 | Dhx9 | | 0.38 | | 27.68 | | 28.25 | 27.88 | 27.7 | 28.24 | 28.23 | 28.45 | 28.05 | 28.3 |  |  |
| P97370 | Atp1b3 | | 0.5 | | 27.67 | | 26.38 | 25.5 | 27 | 27.1 | 27.35 | 27.57 | 27.97 | 26.35 |  |  |
| P40142 | Tkt | | 0.46 | | 27.67 | | 27.78 | 27.29 | 27.75 | 28 | 27.32 | 28.09 | 27.62 | 27.95 |  |  |
| P10639 | Txn | | 0.84 | | 27.63 | | 27.31 | 27.35 | 27.31 | 27.97 | 27.06 | 27.76 | 28.16 | 26.98 |  |  |
| Q8R0J7 | Vps37b | | 0.93 | | 27.63 | | 27.05 | 27.49 | 27.05 | 26.93 | 27.93 | 27.72 | 27.49 | 27.09 |  |  |
| Q3U741 | Ddx17 | | 0.89 | | 27.63 | | 28.1 | 26.44 | 27.78 | 27.87 | 26.53 | 27.94 | 27.6 | 27.33 |  |  |
| Q9EQP2 | Ehd4 | | 0.18 | | 27.62 | | 26.26 | 26.41 | 26.76 | 26.05 | 25.94 | 26.93 | 27.37 | 27.16 |  |  |
| P61255 | Rpl26 | | 0.12 | | 27.62 | | 27.89 | 27.07 | 26.5 | 26.91 | 27.3 | 27.25 | 27.81 | 27.67 |  |  |
| Q62192 | Cd180 | | 0.66 | | 27.61 | | 25.28 | 24.86 | 26.25 | 25.53 | 25.58 | 27.39 | 27.67 | 24.91 |  |  |
| Q9QZD9 | Eif3i | | 0.97 | | 27.61 | | 27.89 | 26.54 | 27.49 | 28.19 | 26.77 | 27.85 | 28.12 | 26.44 |  |  |
| P60122 | Ruvbl1 | | 0.79 | | 27.61 | | 28.4 | 27.73 | 28.31 | 28.42 | 27.59 | 28.31 | 28.42 | 27.67 |  |  |
| E9Q9F5 | 43350 | | 0.82 | | 27.61 | | 27.9 | 27.77 | 28.09 | 27.59 | 27.56 | 27.83 | 27.42 | 27.71 |  |  |
| P61514 | Rpl37a | | 0.26 | | 27.6 | | 27.67 | 27.44 | 27.53 | 27.76 | 27.76 | 27.03 | 27.8 | 25.96 |  |  |
| F8WJK8 | St13 | | 0.94 | | 27.6 | | 26.6 | 27.6 | 26.25 | 28.1 | 28 | 27.8 | 27.4 | 27.14 |  |  |
| Q9D8W5 | Psmd12 | | 0.26 | | 27.6 | | 28.27 | 27.86 | 28.67 | 28.61 | 27.97 | 28.42 | 28.07 | 27.89 |  |  |
| Q8R010 | Aimp2 | | 0.81 | | 27.59 | | 27.59 | 27.44 | 27.65 | 27.74 | 28 | 28.2 | 27.95 | 26.61 |  |  |
| E9Q855 | Scamp3 | | 1 | | 27.58 | | 25.69 | 26.5 | 27.22 | 26.96 | 25.52 | 26.19 | 26.51 | 26.98 |  |  |
| Q8CI94 | Pygb | | 0.83 | | 27.58 | | 28.23 | 27.48 | 28.29 | 28.15 | 27.38 | 27.71 | 27.45 | 28.09 |  |  |
| B7ZCP4 | Cpne1 | | 0.98 | | 27.57 | | 26.63 | 25.54 | 26.69 | 26.22 | 26.67 | 26.92 | 27.18 | 25.84 |  |  |
| Q3TXS7 | Psmd1 | | 0.57 | | 27.55 | | 27.49 | 27.41 | 27.76 | 28.09 | 27.46 | 27.79 | 27.79 | 26.29 |  |  |
| P62962 | Pfn1 | | 0.27 | | 27.55 | | 27.74 | 27.5 | 27.61 | 27.22 | 27.49 | 27.85 | 27.91 | 27.45 |  |  |
| Q3THS6 | Mat2a | | 0.45 | | 27.55 | | 27.46 | 27.23 | 27.16 | 27.64 | 27.04 | 27.47 | 27.49 | 27.58 |  |  |
| Q3UPL0 | Sec31a | | 0.52 | | 27.55 | | 28.2 | 28.24 | 27.85 | 28.39 | 28.64 | 28.13 | 28.43 | 28.24 |  |  |
| P62843 | Rps15 | | 0.8 | | 27.54 | | 28.27 | 26.61 | 25.88 | 27.98 | 27.17 | 26.91 | 27.86 | 26.51 |  |  |
| Q62433 | Ndrg1 | | 0.67 | | 27.54 | | 27.84 | 27.45 | 27.88 | 27.31 | 27.12 | 27.78 | 27.45 | 27.64 |  |  |
| P12960 | Cntn1 | | 0.47 | | 27.54 | | 27.8 | 28.71 | 27.86 | 28.21 | 28.23 | 27.73 | 27.34 | 27.97 |  |  |
| Q61830 | Mrc1 | | 0.65 | | 27.53 | | 24.74 | 27.52 | 27.54 | 27.18 | 27.5 | 27.07 | 27.68 | 26.21 |  |  |
| F8VQC1 | Srp72 | | 0.74 | | 27.52 | | 25.37 | 26.08 | 25.95 | 25.69 | 25.8 | 25.83 | 27.42 | 25.51 |  |  |
| P08207 | S100a10 | | 0.69 | | 27.52 | | 27.01 | 26.51 | 27.61 | 27.61 | 27.54 | 27.85 | 28.01 | 25.66 |  |  |
| A0A0N4SW94 | Myadm | | 0.61 | | 27.52 | | 27.24 | 26.52 | 27.66 | 26.92 | 27.74 | 27.02 | 27.56 | 26.83 |  |  |
| K3W4R2 | Myh14 | | 0.5 | | 27.52 | | 27.56 | 27.55 | 27.53 | 26.15 | 27.6 | 27.28 | 26.62 | 27.3 |  |  |
| Q8BWP8 | B4gat1 | | 0.57 | | 27.52 | | 27.69 | 28.94 | 27.51 | 28.27 | 27.64 | 27.45 | 27.22 | 27.97 |  |  |
| P61222 | Abce1 | | 0.84 | | 27.51 | | 27.61 | 26.61 | 27.69 | 27.74 | 27.1 | 27.81 | 28.1 | 25.22 |  |  |
| Q8VHB5 | Ca9;Car9 | | 0.81 | | 27.51 | | 26.72 | 26.91 | 26.82 | 26.97 | 28.02 | 27.43 | 27.69 | 26.79 |  |  |
| E9QN08 | Eef1d | | 0.89 | | 27.51 | | 27.74 | 27.23 | 27.39 | 28.1 | 27.17 | 27.74 | 27.39 | 27.09 |  |  |
| O70456 | Sfn | | 0.46 | | 27.51 | | 27.95 | 27.39 | 28.34 | 27.72 | 27.72 | 27.52 | 27.83 | 27.82 |  |  |
| Q6ZWZ6 | Rps12 | | 0.31 | | 27.5 | | 27.37 | 27.98 | 27.34 | 27.91 | 26.85 | 27.25 | 27.3 | 25.05 |  |  |
| Q64310 | Surf4 | | 0.79 | | 27.5 | | 25.97 | 26.22 | 27.15 | 24.81 | 26.37 | 27.06 | 27.35 | 25.53 |  |  |
| P27773 | Pdia3 | | 0.8 | | 27.5 | | 27.41 | 26.66 | 27.38 | 26.48 | 27.76 | 27.9 | 27.54 | 26.93 |  |  |
| Q8C0E3 | Trim47 | | 0.07 | | 27.5 | | 27.63 | 27.73 | 27.98 | 27.56 | 28.09 | 27.6 | 27.28 | 27.32 |  |  |
| Q9D7S7 | Rpl22l1 | | 0.34 | | 27.49 | | 27.5 | 26 | 26.17 | 25.77 | 26.83 | 25.27 | 26.64 | 26.42 |  |  |
| Q8BMF4 | Dlat | | 0.83 | | 27.49 | | 26.21 | 26.86 | 29.43 | 25.87 | 26.87 | 27.22 | 27.9 | 26.88 |  |  |
| Q9DBZ5 | Eif3k | | 0.38 | | 27.49 | | 27.67 | 27.17 | 28.29 | 28.65 | 26.96 | 28.5 | 28.78 | 27.46 |  |  |
| D3Z1S1 | 43345 | | 0.29 | | 27.49 | | 27.58 | 26.21 | 28.03 | 27.9 | 27.34 | 27.59 | 27.79 | 27.53 |  |  |
| Q9JKR6 | Hyou1 | | 0.31 | | 27.49 | | 27.69 | 26.99 | 25.7 | 27.23 | 26.61 | 26.87 | 26.53 | 27.93 |  |  |
| Q9CWK8 | Snx2 | | 0.63 | | 27.48 | | 27.73 | 26.4 | 27.61 | 28.66 | 26.67 | 27.87 | 27.83 | 27.55 |  |  |
| Q91YL3 | Uckl1 | | 0.15 | | 27.48 | | 27.33 | 27.15 | 27.06 | 26.95 | 27.62 | 27.46 | 27.73 | 27.83 |  |  |
| D3Z598 | Ltbp4 | | 0.79 | | 27.47 | | 25.46 | 27.86 | 27.64 | 27.75 | 26.82 | 27.94 | 27.71 | 26.45 |  |  |
| P63024 | Vamp3 | | 0.93 | | 27.47 | | 27.63 | 26.82 | 26.81 | 27.06 | 27.72 | 26.85 | 27.66 | 27.03 |  |  |
| P10833 | Rras | | 0.87 | | 27.46 | | 26.34 | 24.74 | 26.72 | 24.8 | 26.05 | 26.08 | 26.28 | 26.42 |  |  |
| A0A0G2JEU1 | Aldh2 | | 0.27 | | 27.46 | | 27.16 | 26.71 | 26.92 | 26.26 | 26.58 | 27.12 | 27.3 | 26.53 |  |  |
| Q3TF41 | Nap1l1 | | 0.44 | | 27.46 | | 27.61 | 27.07 | 26.71 | 27.56 | 27.03 | 27.57 | 27.7 | 27.13 |  |  |
| G3UWC2 | Naalad2 | | 0.71 | | 27.46 | | 27.36 | 26.45 | 27.03 | 26.98 | 27.04 | 26.68 | 27.62 | 27.65 |  |  |
| E9PV24 | Fga | | 0.79 | | 27.46 | | 27.01 | 28.45 | 27.42 | 28.63 | 27.32 | 27.34 | 27.18 | 27.79 |  |  |
| Q9R0G6 | Comp | | 0.58 | | 27.45 | | 25.82 | 28.32 | 25.35 | 27.46 | 26 | 27.64 | 26.2 | 26.68 |  |  |
| Q9DB05 | Napa | | 0.91 | | 27.44 | | 26.39 | 26.91 | 26.89 | 27.11 | 27.11 | 26.67 | 27.44 | 26.59 |  |  |
| Q9WVA4 | Tagln2 | | 0.74 | | 27.44 | | 27.56 | 28.13 | 27.75 | 27.44 | 27.53 | 27.68 | 27.77 | 27.05 |  |  |
| P61164 | Actr1a | | 0.23 | | 27.44 | | 28.06 | 27.27 | 27.65 | 28.16 | 27.42 | 28.4 | 28.28 | 27.8 |  |  |
| Q64727 | Vcl | | 0.69 | | 27.43 | | 28.29 | 28.44 | 27.38 | 27.84 | 28.17 | 27.67 | 27.78 | 27.99 |  |  |
| Q6P8Q0 | Gsta1 | | 0.52 | | 27.42 | | 28.86 | 27.62 | 28.8 | 28.16 | 28.49 | 27.82 | 28.55 | 29.02 |  |  |
| Q6P4T2 | Snrnp200 | | 0.07 | | 27.4 | | 27.58 | 27.48 | 28.39 | 27.81 | 28.46 | 27.9 | 27.99 | 27.27 |  |  |
| P62743 | Ap2s1 | | 0.49 | | 27.4 | | 27.69 | 26.07 | 27.7 | 27.42 | 27.34 | 27.41 | 27.81 | 27.47 |  |  |
| D3YYK8 | Mapre2 | | 0.52 | | 27.4 | | 27.75 | 28.19 | 28.31 | 28.07 | 28.06 | 28.3 | 27.45 | 28.37 |  |  |
| O35593 | Psmd14 | | 0.43 | | 27.37 | | 27.46 | 26.62 | 27.38 | 26.93 | 27.52 | 28.21 | 27.59 | 27.12 |  |  |
| P61027 | Rab10 | | 0.87 | | 27.36 | | 27.09 | 26.76 | 26.91 | 27.11 | 27.75 | 27.32 | 27.62 | 26.56 |  |  |
| Q9R190 | Mta2 | | 0.73 | | 27.36 | | 26.35 | 26.87 | 25.92 | 27.17 | 26.73 | 26.91 | 26.83 | 26.94 |  |  |
| Q9Z1N5 | Ddx39b | | 0.34 | | 27.36 | | 27.82 | 27.04 | 28 | 27.59 | 27.37 | 27.66 | 27.79 | 27.95 |  |  |
| O54962 | Banf1 | | 0.74 | | 27.35 | | 26.19 | 27.29 | 27.74 | 26.87 | 26.74 | 27.03 | 27.72 | 27.13 |  |  |
| P35282 | Rab21 | | 0.92 | | 27.33 | | 26.03 | 26.43 | 26.63 | 26.06 | 26.5 | 27.05 | 26.83 | 25.62 |  |  |
| E9Q616 | Ahnak | | 0.23 | | 27.33 | | 26.54 | 26.56 | 27.44 | 27.61 | 26.76 | 28 | 28.11 | 26.88 |  |  |
| P59999 | Arpc4 | | 0.33 | | 27.33 | | 27.7 | 27.08 | 28.04 | 27.41 | 27.65 | 27.53 | 27.64 | 27.85 |  |  |
| Q8CBB7 | Ap1g1 | | 0.24 | | 27.32 | | 29.2 | 27.13 | 28.69 | 27.74 | 26.57 | 26.78 | 26.29 | 26.63 |  |  |
| D6RGM7 | Katnal2 | | 0.51 | | 27.32 | | 27.31 | 27.3 | 23.45 | 26.94 | 27.07 | 27.13 | 23.29 | 26.99 |  |  |
| Q9CVB6 | Arpc2 | | 0.39 | | 27.32 | | 27.56 | 27.32 | 27.62 | 29.19 | 27.59 | 27.27 | 28.26 | 27.73 |  |  |
| P42669 | Pura | | 0.56 | | 27.31 | | 27.66 | 26.87 | 26.93 | 27.41 | 26.42 | 26.76 | 27.29 | 27 |  |  |
| Q99PT1 | Arhgdia | | 0.26 | | 27.31 | | 25.57 | 27.7 | 27.41 | 28.03 | 26.93 | 28.03 | 28.01 | 27.86 |  |  |
| Q3TH01 | H2-K1 | | 0.49 | | 27.3 | | 27.08 | 26.76 | 27.74 | 27.73 | 27.54 | 27.48 | 27.8 | 24.89 |  |  |
| Z4YKA3 | Hp1bp3 | | 0.84 | | 27.29 | | 27 | 25.8 | 26.82 | 26.63 | 26.47 | 26.7 | 26.67 | 25.99 |  |  |
| Q61937 | Npm1 | | 0.89 | | 27.29 | | 26.9 | 27 | 26.35 | 27.35 | 26.98 | 27.49 | 27.01 | 26.48 |  |  |
| K3W4Q8 | Bsg | | 0.06 | | 27.29 | | 25.48 | 26.13 | 27.85 | 27.83 | 27.4 | 27.07 | 27.23 | 26.86 |  |  |
| Q6KAU4 | Mvb12b | | 0.79 | | 27.28 | | 25.99 | 26.67 | 26.65 | 26.65 | 27.58 | 26.99 | 27.09 | 26.12 |  |  |
| A0A2I3BPG9 | Rpl36a | | 0.52 | | 27.28 | | 27.05 | 27.32 | 27.15 | 26.53 | 27.27 | 26.91 | 27.07 | 27.09 |  |  |
| F8WIR1 | Ctsd | | 0.32 | | 27.28 | | 26.44 | 27.51 | 27.51 | 27.54 | 27.35 | 27.61 | 27.42 | 27.42 |  |  |
| Q9JK53 | Prelp | | 0.97 | | 27.28 | | 26.78 | 28.18 | 27.19 | 27.86 | 27.49 | 27.64 | 26.87 | 27.82 |  |  |
| Q8BT60 | Cpne3 | | 0.74 | | 27.27 | | 26.03 | 25.63 | 26.14 | 26.42 | 26.43 | 27.09 | 26.99 | 25.93 |  |  |
| Q9Z0N1 | Eif2s3x | | 0.59 | | 27.27 | | 27.36 | 27.16 | 27.14 | 27.61 | 26.37 | 26.61 | 27.3 | 26.77 |  |  |
| P48758 | Cbr1 | | 0.28 | | 27.26 | | 26.07 | 26.59 | 27.18 | 27.19 | 26.81 | 26.34 | 26.93 | 25.34 |  |  |
| Q8R2Z5 | Vwa1 | | 0.3 | | 27.25 | | 27.05 | 27.77 | 25.35 | 23.25 | 27.71 | 25.37 | 26.9 | 26.48 |  |  |
| Q60715 | P4ha1 | | 0.28 | | 27.24 | | 26.99 | 26.34 | 26.71 | 26.42 | 26.6 | 27.13 | 26.85 | 27.01 |  |  |
| Q9DCL9 | Paics | | 0.3 | | 27.24 | | 28.2 | 27.38 | 28.16 | 27.99 | 27.87 | 27.86 | 28.04 | 28.04 |  |  |
| Q9Z0E6 | Gbp2 | | 0.71 | | 27.23 | | 25.68 | 25.03 | 26.78 | 25 | 25.59 | 27.46 | 26.58 | 25.38 |  |  |
| D3Z4N2 | Spp1 | | 0.66 | | 27.23 | | 27.16 | 25.94 | 28.18 | 28.45 | 26.05 | 28.61 | 28.98 | 25.71 |  |  |
| A0A2R8VI30 | Cd47 | | 0.46 | | 27.23 | | 26.26 | 24.93 | 25.58 | 26.89 | 26.72 | 26.88 | 27.36 | 26.75 |  |  |
| P61750 | Arf4 | | 0.08 | | 27.23 | | 27.17 | 27.03 | 27.47 | 27.6 | 27.41 | 26.78 | 27.36 | 27.14 |  |  |
| Q9Z2X1 | Hnrnpf | | 0.59 | | 27.22 | | 27.28 | 26.75 | 27.02 | 27.47 | 27.12 | 26.99 | 27.3 | 26.47 |  |  |
| Q91V41 | Rab14 | | 0.07 | | 27.22 | | 28.18 | 27.45 | 26.98 | 26.94 | 26.6 | 27.58 | 27.38 | 28.27 |  |  |
| Q99LF4 | Rtcb | | 0.09 | | 27.21 | | 26.75 | 26.7 | 26.93 | 27.19 | 26.72 | 27.44 | 27.52 | 27.15 |  |  |
| P43275 | Hist1h1a | | 0.63 | | 27.19 | | 27.01 | 17.5 | 26.56 | 27.55 | 25.66 | 26.91 | 27.5 | 23.42 |  |  |
| Q8CG29 | Myo1f | | 0.51 | | 27.19 | | 27 | 27.82 | 27.48 | 26.93 | 27.65 | 27.14 | 27.29 | 26.55 |  |  |
| Q8BKG3 | Ptk7 | | 0.56 | | 27.19 | | 28.09 | 24.95 | 27.55 | 27.77 | 27.54 | 27.08 | 27.04 | 27.63 |  |  |
| Q9QZC7 | Plekhb2 | | 0.13 | | 27.18 | | 26.03 | 27.14 | 27.52 | 27.85 | 28.25 | 26.95 | 27.61 | 26.08 |  |  |
| P99027 | Rplp2 | | 0.26 | | 27.17 | | 27.09 | 28.51 | 31.06 | 28.05 | 27.94 | 27.45 | 27.56 | 27.67 |  |  |
| P63242 | Eif5a | | 0.87 | | 27.16 | | 26.89 | 29.19 | 26.96 | 27.83 | 28.18 | 27.07 | 28.33 | 28.78 |  |  |
| G5E829 | Atp2b1 | | 0.85 | | 27.15 | | 27.33 | 24.91 | 26.65 | 25.44 | 25.96 | 26.85 | 26.47 | 25.18 |  |  |
| A0A0R4J1C8 | Cd68 | | 0.69 | | 27.15 | | 26.99 | 26.8 | 27.41 | 26.91 | 27.78 | 27.76 | 27.92 | 26.31 |  |  |
| Q9R0N0 | Galk1 | | 0.46 | | 27.15 | | 27.78 | 27.7 | 27.52 | 27.85 | 27.31 | 27.35 | 26.73 | 27.56 |  |  |
| A0A0R4J005 | Lims1 | | 0.84 | | 27.14 | | 24.81 | 26.87 | 26.82 | 27.17 | 26.32 | 27.24 | 27.09 | 24.98 |  |  |
| P55258 | Rab8a | | 0.53 | | 27.13 | | 25.96 | 26.1 | 26.84 | 26.06 | 26.84 | 26.92 | 27.16 | 26.53 |  |  |
| F6XC25 | Cc2d1b | | 0.89 | | 27.13 | | 26.85 | 25.44 | 27.1 | 25.36 | 26.91 | 26.77 | 26.72 | 26.69 |  |  |
| P68510 | Ywhah | | 0.6 | | 27.13 | | 27.61 | 25.9 | 27.41 | 27.04 | 27.04 | 27.23 | 27.18 | 27.6 |  |  |
| G3UZ34 | Eftud2 | | 0.11 | | 27.13 | | 27.56 | 26.45 | 27.03 | 27.12 | 27.56 | 27.73 | 27.93 | 27.67 |  |  |
| Q99K48 | Nono | | 0.92 | | 27.12 | | 27.13 | 25.74 | 27.06 | 27 | 25.68 | 26.96 | 26.95 | 26.49 |  |  |
| Q00612 | G6pdx | | 0.71 | | 27.1 | | 27.82 | 26.62 | 27.84 | 28.28 | 26.27 | 28.05 | 28.06 | 27.06 |  |  |
| Q9DBH5 | Lman2 | | 0.27 | | 27.09 | | 25.55 | 25.64 | 27.3 | 25.92 | 27.33 | 27.34 | 27.45 | 26.62 |  |  |
| Q5SWU9 | Acaca | | 0.47 | | 27.09 | | 27.8 | 26.42 | 26.07 | 27.2 | 26.95 | 27.39 | 27.19 | 27.32 |  |  |
| Q6P5F9 | Xpo1 | | 0.82 | | 27.08 | | 26.3 | 27.75 | 27.17 | 27.04 | 27.6 | 27.64 | 27.18 | 26.99 |  |  |
| P62320 | Snrpd3 | | 0.72 | | 27.07 | | 26.35 | 25.99 | 26.8 | 26.31 | 25.61 | 26.57 | 26.75 | 24.22 |  |  |
| Q99LB4 | Capg | | 0.92 | | 27.07 | | 25.89 | 25.03 | 26.17 | 25.96 | 25.16 | 25.88 | 27.18 | 25.08 |  |  |
| P43277 | Hist1h1d | | 0.97 | | 27.07 | | 27.48 | 26.73 | 26.92 | 27.83 | 26.21 | 27.71 | 27.5 | 26.19 |  |  |
| O88844 | Idh1 | | 0.21 | | 27.07 | | 26.91 | 26.7 | 26.83 | 27 | 26.91 | 26.62 | 26.69 | 26.84 |  |  |
| P40240 | Cd9 | | 0.49 | | 27.06 | | 17.5 | 25.1 | 26.73 | 24.41 | 24.77 | 25.06 | 27.49 | 26.51 |  |  |
| Q6P069 | Sri | | 0.84 | | 27.06 | | 27.48 | 27.55 | 27 | 27.49 | 27.46 | 27.49 | 27.24 | 27.57 |  |  |
| Q3TKD0 | Tnpo1 | | 0.58 | | 27.05 | | 25.73 | 26.71 | 26.67 | 27 | 26.91 | 26.83 | 27.27 | 26.46 |  |  |
| F8WIV2 | Serpinb6a | | 0.78 | | 27.05 | | 26.76 | 26.19 | 26.56 | 26.56 | 26.17 | 26.99 | 25.97 | 26.56 |  |  |
| A0A0N4SVB8 | Arl8a | | 0.32 | | 27.05 | | 26.33 | 26.7 | 25.79 | 26.82 | 26.44 | 26.9 | 27.02 | 26.65 |  |  |
| Q9DC51 | Gnai3 | | 0.84 | | 27.04 | | 26.76 | 25.13 | 26.24 | 26.53 | 26.56 | 27.4 | 27.41 | 25.38 |  |  |
| P63330 | Ppp2ca | | 0.38 | | 27.04 | | 25.95 | 26.6 | 26.44 | 26.2 | 25.95 | 27.39 | 26.44 | 26.48 |  |  |
| A0A0R4J0R1 | Vamp8 | | 0.75 | | 27.03 | | 27.11 | 24.07 | 26.66 | 26.04 | 27.11 | 26.5 | 26.81 | 26.79 |  |  |
| Q02013 | Aqp1 | | 0.83 | | 27.02 | | 24.6 | 25.86 | 26.35 | 26.53 | 26.06 | 26.71 | 26.99 | 24.75 |  |  |
| Q4FE56 | Usp9x | | 0.87 | | 27.02 | | 27.18 | 25.12 | 26.97 | 27.57 | 26.09 | 27.08 | 27.53 | 25.48 |  |  |
| Q8R0Y6 | Aldh1l1 | | 0.16 | | 27.02 | | 27.86 | 28.39 | 27.07 | 27.22 | 26.16 | 27.65 | 27.26 | 27.87 |  |  |
| P97464 | Ext1 | | 0.79 | | 27.01 | | 27.2 | 28.35 | 27.53 | 27.93 | 27.82 | 27.78 | 27.64 | 27.66 |  |  |
| Q8BKC5 | Ipo5 | | 0.73 | | 27 | | 26.82 | 26.43 | 27.22 | 26.24 | 26.85 | 27.52 | 26.97 | 26.54 |  |  |
| P53986 | Slc16a1 | | 0.2 | | 26.99 | | 27.4 | 27.34 | 27.07 | 26.82 | 27.76 | 26.98 | 26.65 | 24.72 |  |  |
| B8JK33 | Hnrnpm | | 0.4 | | 26.99 | | 26.3 | 23.93 | 26.67 | 27.09 | 26.94 | 26.84 | 25.94 | 26.37 |  |  |
| P08226 | Apoe | | 0.18 | | 26.99 | | 27.37 | 27.81 | 24.84 | 17.5 | 26.5 | 26.4 | 26.94 | 28.06 |  |  |
| E0CYH4 | Wdr26 | | 0.14 | | 26.98 | | 26.55 | 26.38 | 26.29 | 25.79 | 24.69 | 27.06 | 26.7 | 25.92 |  |  |
| Q9R0P5 | Dstn | | 0.45 | | 26.98 | | 25.52 | 26.78 | 26.67 | 27.39 | 26.97 | 26.68 | 26.88 | 25.99 |  |  |
| P70349 | Hint1 | | 0.41 | | 26.98 | | 26.98 | 27.09 | 26.7 | 26.8 | 26.34 | 27.05 | 27.24 | 26.22 |  |  |
| E9PX84 | Dab2 | | 0.98 | | 26.97 | | 27.4 | 22.95 | 27.01 | 26.24 | 23.78 | 26.36 | 26.83 | 23.41 |  |  |
| Q6ZQ38 | Cand1 | | 0.94 | | 26.97 | | 27.2 | 27.13 | 27.22 | 26.97 | 27 | 26.87 | 26.91 | 27.65 |  |  |
| Q8C129 | Lnpep | | 0.3 | | 26.96 | | 26.13 | 25.89 | 26.8 | 25.99 | 26.08 | 26.69 | 26.83 | 26.94 |  |  |
| P35564 | Canx | | 0.9 | | 26.95 | | 24.73 | 26.75 | 26.59 | 25.52 | 27.09 | 26.1 | 26.92 | 26.4 |  |  |
| Q6ZWV7 | Rpl35 | | 0.97 | | 26.95 | | 27.31 | 25.16 | 26.97 | 26.29 | 25.94 | 24.87 | 27.43 | 27.54 |  |  |
| Q9QUR6 | Prep | | 0.33 | | 26.94 | | 25.54 | 28.02 | 26.27 | 24.89 | 25.93 | 25.02 | 26.75 | 25.33 |  |  |
| O35646 | Capn6 | | 0.99 | | 26.94 | | 26.6 | 23.55 | 26.42 | 24.76 | 25.83 | 25.1 | 25.61 | 26.02 |  |  |
| O54833 | Csnk2a2 | | 0.73 | | 26.94 | | 26.89 | 26.13 | 27.06 | 25.06 | 26.48 | 26.15 | 26.75 | 26.22 |  |  |
| P58854 | Tubgcp3 | | 0.73 | | 26.94 | | 26.82 | 26.26 | 26.75 | 26.22 | 26.44 | 26.52 | 27.19 | 26.37 |  |  |
| E9Q6R3 | Sec22b | | 0.57 | | 26.94 | | 25.59 | 26.6 | 25.8 | 25.81 | 27.11 | 26.56 | 26.92 | 26.84 |  |  |
| J7NUP1 | Irgm1 | | 0.55 | | 26.91 | | 26.71 | 27 | 25.9 | 26.88 | 26.02 | 27.32 | 27.51 | 25.52 |  |  |
| P11688 | Itga5 | | 0.47 | | 26.9 | | 26.48 | 26.2 | 27.22 | 26.57 | 26.55 | 26.83 | 26.21 | 26.13 |  |  |
| P50518 | Atp6v1e1 | | 0.29 | | 26.9 | | 26.41 | 24.94 | 25.27 | 27.57 | 25.43 | 27.46 | 27.22 | 27.1 |  |  |
| Q05DV1 | Por | | 0.53 | | 26.88 | | 24.94 | 25.05 | 26.08 | 26.31 | 26.57 | 25.81 | 27.28 | 25.8 |  |  |
| Q9D1M4 | Eef1e1 | | 0.6 | | 26.88 | | 27.42 | 24.76 | 26.18 | 27.33 | 26.54 | 27.68 | 27.59 | 26.34 |  |  |
| Q9D6Z1 | Nop56 | | 0.65 | | 26.87 | | 26.2 | 25.42 | 27.3 | 25.12 | 26.22 | 26.45 | 25.71 | 24.5 |  |  |
| A0A0A6YVT1 | Cr1l | | 0.88 | | 26.87 | | 24.93 | 26.13 | 25.91 | 25.84 | 26.9 | 26.34 | 27.03 | 25.48 |  |  |
| Q11136 | Pepd | | 0.95 | | 26.86 | | 26.31 | 26.81 | 27.05 | 26.83 | 26.39 | 27 | 26 | 27.04 |  |  |
| P08249 | Mdh2 | | 0.9 | | 26.86 | | 26.4 | 27.65 | 26.87 | 27.7 | 26.77 | 27.39 | 26.85 | 27.21 |  |  |
| G3UW94 | U2af1 | | 0.63 | | 26.85 | | 26.24 | 24.61 | 26.27 | 24.51 | 24.69 | 26.82 | 25.11 | 25.51 |  |  |
| A2A6U3 | 43352 | | 0.72 | | 26.84 | | 26.9 | 24.82 | 25.24 | 26.67 | 28.63 | 26.61 | 26.54 | 24.75 |  |  |
| A0A1B0GR08 | Tubgcp2 | | 0.79 | | 26.84 | | 26.93 | 26.34 | 27.24 | 26.6 | 26.29 | 26.77 | 27.16 | 25.15 |  |  |
| P62317 | Snrpd2 | | 0.46 | | 26.83 | | 26.92 | 26.88 | 25.69 | 27.27 | 26.78 | 26.82 | 26.95 | 17.5 |  |  |
| Q7TNC4 | Luc7l2 | | 0.96 | | 26.83 | | 26.91 | 23.28 | 25.38 | 24.07 | 26.46 | 25.96 | 26.75 | 23.9 |  |  |
| H7BWZ3 | Arpc3 | | 0.68 | | 26.83 | | 27.18 | 26.84 | 27.33 | 27.1 | 26.93 | 27.27 | 27.15 | 26.74 |  |  |
| Q05144 | Rac2 | | 0.32 | | 26.82 | | 25.98 | 24.54 | 26.55 | 27.85 | 26.62 | 26.03 | 27.15 | 25.72 |  |  |
| Q61646 | Hp | | 0.72 | | 26.82 | | 27.61 | 28.54 | 27.31 | 26.98 | 28.27 | 28.25 | 28.28 | 27.4 |  |  |
| Q9Z1T1 | Ap3b1 | | 0.97 | | 26.81 | | 27.25 | 25.33 | 27.09 | 26.83 | 25.91 | 27.43 | 27.16 | 24.7 |  |  |
| P62331 | Arf6 | | 1 | | 26.8 | | 26.05 | 26.85 | 25.5 | 26.91 | 27.25 | 26.38 | 27.05 | 26.23 |  |  |
| Q3TGU7 | Pa2g4 | | 0.28 | | 26.8 | | 25.03 | 26.75 | 25.91 | 24.05 | 25.52 | 25.82 | 26.52 | 26.39 |  |  |
| Q8C0C7 | Farsa | | 0.37 | | 26.78 | | 26.34 | 26.57 | 27.75 | 27.4 | 25.49 | 25.44 | 26.57 | 25.7 |  |  |
| P21981 | Tgm2 | | 0.71 | | 26.77 | | 25.83 | 25.52 | 27.75 | 26.39 | 25.75 | 26.77 | 26.74 | 24.9 |  |  |
| Q922D8 | Mthfd1 | | 0.88 | | 26.77 | | 28.42 | 26.84 | 27.02 | 27.74 | 26.88 | 27.04 | 27.6 | 26.55 |  |  |
| Q9ER00 | Stx12 | | 0.16 | | 26.75 | | 26.87 | 26.69 | 25.02 | 27.08 | 27.37 | 25.83 | 25.01 | 25.48 |  |  |
| Q8K1I3 | Spp2 | | 0.91 | | 26.75 | | 27.38 | 25.97 | 27.1 | 26.93 | 26.49 | 26.81 | 26.58 | 26.69 |  |  |
| Q8BHN3 | Ganab | | 0.22 | | 26.74 | | 26.41 | 26.27 | 27.17 | 26.8 | 26.48 | 26.45 | 26.6 | 26.25 |  |  |
| O35316 | Slc6a6 | | 0.89 | | 26.74 | | 27.31 | 26.5 | 26.83 | 26.53 | 27.35 | 26.92 | 26.95 | 26.4 |  |  |
| Q8BH35 | C8b | | 0.29 | | 26.74 | | 26.74 | 27.54 | 27.46 | 27.47 | 27.11 | 27.04 | 27.02 | 26.73 |  |  |
| A0A2U3TZ67 | Dnm1l | | 0.19 | | 26.72 | | 25.56 | 26.91 | 26.22 | 26.17 | 25.38 | 26.04 | 25.21 | 24.45 |  |  |
| Q00519 | Xdh | | 0.27 | | 26.71 | | 24.32 | 20.95 | 27.23 | 27.62 | 24.31 | 24.38 | 23.84 | 22.66 |  |  |
| Q9JJ00 | Plscr1 | | 0.23 | | 26.71 | | 26.19 | 26.61 | 26.3 | 25.45 | 26.05 | 25.93 | 26.54 | 25.82 |  |  |
| D3YWF6 | Otub1 | | 0.73 | | 26.71 | | 26.73 | 27.25 | 27.11 | 27.15 | 26.82 | 26.74 | 27.01 | 26.97 |  |  |
| E9Q390 | Myof | | 0.43 | | 26.7 | | 24.64 | 20.61 | 27.17 | 25.24 | 25.1 | 26.53 | 26.42 | 25.01 |  |  |
| A0A140T8V5 | Pcna | | 0.13 | | 26.7 | | 26.64 | 26.49 | 25.2 | 26.15 | 25.67 | 26.88 | 26.11 | 25.53 |  |  |
| Q9QZH3 | Ppie | | 0.34 | | 26.7 | | 26.34 | 27.13 | 26.11 | 26.86 | 26.88 | 27.14 | 26.74 | 27.49 |  |  |
| Q8R105 | Vps37c | | 0.32 | | 26.69 | | 27.5 | 27.28 | 27.22 | 26.83 | 27.8 | 25.38 | 27.34 | 26.55 |  |  |
| Q9JIZ9 | Plscr3 | | 0.68 | | 26.66 | | 26.33 | 24.46 | 25.44 | 24.66 | 25.85 | 25.99 | 26.63 | 25.14 |  |  |
| Q9D1G1 | Rab1b | | 0.08 | | 26.66 | | 27.28 | 26.3 | 26.41 | 26.7 | 26.65 | 26 | 25.78 | 26.26 |  |  |
| B1AVZ0 | Uprt | | 0.75 | | 26.66 | | 26.36 | 25.22 | 26.32 | 26.21 | 26.38 | 25.58 | 26.79 | 27.11 |  |  |
| Q9CPW4 | Arpc5 | | 0.05 | | 26.65 | | 26.75 | 26.87 | 25.1 | 25.79 | 25.7 | 26.94 | 26.65 | 25.48 |  |  |
| A0A2I3BR03 | App | | 0.36 | | 26.65 | | 26.76 | 27.45 | 25.92 | 26.57 | 26.61 | 26.75 | 27.29 | 26.17 |  |  |
| P47856 | Gfpt1 | | 0.44 | | 26.64 | | 26.89 | 25.17 | 26.25 | 26.4 | 26.13 | 26.01 | 26.2 | 23.84 |  |  |
| A2AMW0 | Capzb | | 0.3 | | 26.64 | | 26.26 | 26.1 | 26.76 | 26.54 | 26.44 | 27.05 | 26.95 | 26.28 |  |  |
| Q9R1Q7 | Plp2 | | 0.86 | | 26.63 | | 26.02 | 24.68 | 24.87 | 26.73 | 26.89 | 26.7 | 26.8 | 25.07 |  |  |
| Q9CPY7 | Lap3 | | 0.67 | | 26.63 | | 26.96 | 26.52 | 27.19 | 26.76 | 26.62 | 26.78 | 26.89 | 26.82 |  |  |
| O88811 | Stam2 | | 0.44 | | 26.63 | | 31.12 | 31.5 | 31.07 | 27.01 | 28.14 | 31.06 | 30.57 | 31.2 |  |  |
| Q61009 | Scarb1 | | 0.75 | | 26.6 | | 26.66 | 25.54 | 23.86 | 26.59 | 26.87 | 25.49 | 25.7 | 25.78 |  |  |
| A0A0A6YWP6 | Atp6v1h | | 0.44 | | 26.6 | | 24.94 | 26.26 | 26.38 | 26.55 | 26.31 | 26.98 | 26.68 | 26.02 |  |  |
| A0A2I3BQH3 | Hnrnpc | | 0.87 | | 26.6 | | 25.42 | 25.5 | 26.66 | 25.6 | 25.74 | 25.53 | 26.49 | 26.24 |  |  |
| Q9JHF5 | Tcirg1 | | 0.99 | | 26.59 | | 24.5 | 25.42 | 25.36 | 25.4 | 26.05 | 26.87 | 26.47 | 23.05 |  |  |
| A2AIS9 | Arrdc1 | | 0.95 | | 26.59 | | 27.03 | 27.26 | 26.55 | 26.42 | 27.5 | 26.18 | 26.94 | 27.46 |  |  |
| Q9QUM0 | Itga2b | | 0.35 | | 26.58 | | 17.5 | 25.18 | 26.92 | 25.61 | 26.47 | 26.39 | 25.91 | 26.59 |  |  |
| Q3UZG4 | Aimp1 | | 0.54 | | 26.57 | | 27.72 | 27.29 | 26.14 | 26.99 | 27.12 | 27.45 | 27.57 | 26.6 |  |  |
| P16301 | Lcat | | 0.91 | | 26.57 | | 27.26 | 27.54 | 27.47 | 27.39 | 26.93 | 27.31 | 26.57 | 28.01 |  |  |
| Z4YKV1 | Gnas | | 0.3 | | 26.56 | | 24.78 | 26.02 | 26.43 | 26.52 | 26.92 | 26.64 | 26.28 | 25.85 |  |  |
| Q5XJY5 | Arcn1 | | 0.29 | | 26.56 | | 26.38 | 25.06 | 26.96 | 27.25 | 26.31 | 26.68 | 27.17 | 26.16 |  |  |
| O09131 | Gsto1 | | 0.87 | | 26.56 | | 26.81 | 28.35 | 26.88 | 27.47 | 26.68 | 26.95 | 26.34 | 27.57 |  |  |
| P35550 | Fbl | | 0.12 | | 26.55 | | 26.71 | 26.24 | 26.62 | 25.18 | 25.5 | 25.28 | 24.72 | 25.97 |  |  |
| O09043 | Napsa | | 0.3 | | 26.54 | | 27.16 | 27.08 | 26.28 | 25.87 | 26.91 | 27.01 | 26.32 | 26.91 |  |  |
| Q9CYL5 | Glipr2 | | 0.52 | | 26.54 | | 26.99 | 26.71 | 26.65 | 25.38 | 26.5 | 25.54 | 26.61 | 26.92 |  |  |
| P30412 | Ppic | | 0.12 | | 26.52 | | 26.7 | 26.29 | 26.87 | 26.52 | 26.92 | 25.73 | 25.89 | 26.67 |  |  |
| A2AUR7 | Rsu1 | | 0.12 | | 26.51 | | 27.39 | 27.3 | 27.07 | 26.75 | 26.88 | 26.37 | 25.83 | 26.72 |  |  |
| P27048 | Snrpb | | 0.88 | | 26.5 | | 26.43 | 24.17 | 25.93 | 26.44 | 25.58 | 26.33 | 26.53 | 25.29 |  |  |
| Q8BGJ5 | Ptbp1 | | 0.1 | | 26.5 | | 25.58 | 25.61 | 25.33 | 24.67 | 25.61 | 25.92 | 26.01 | 26.18 |  |  |
| P70460 | Vasp | | 0.95 | | 26.49 | | 25.85 | 27.36 | 27.69 | 26.44 | 25.56 | 26.83 | 26.73 | 26.64 |  |  |
| G3X977 | Itih2 | | 0.46 | | 26.49 | | 27.56 | 27.44 | 27.53 | 27.49 | 27.61 | 27.39 | 27.38 | 26.92 |  |  |
| Q9WUM4 | Coro1c | | 0.62 | | 26.48 | | 25.18 | 25.45 | 26.52 | 27.25 | 25.21 | 26.46 | 26.5 | 25.52 |  |  |
| Q3U9N4 | Grn | | 0.88 | | 26.48 | | 26.25 | 27.01 | 27.32 | 25.86 | 27.18 | 26.72 | 26.94 | 26.04 |  |  |
| P32921 | Wars | | 0.98 | | 26.48 | | 26.44 | 25.55 | 26.99 | 26.23 | 25.35 | 25.43 | 26.95 | 26.4 |  |  |
| A0A0U1RNK7 | Dock7 | | 0.54 | | 26.47 | | 25.95 | 21.84 | 26.42 | 24.54 | 26.49 | 23.99 | 26.06 | 21.53 |  |  |
| A0A0R4J027 | Irg1 | | 0.63 | | 26.47 | | 26.36 | 25.82 | 26.91 | 27.66 | 25.8 | 27.38 | 27.5 | 25.61 |  |  |
| Q62318 | Trim28 | | 0.89 | | 26.46 | | 25.78 | 25.44 | 25.24 | 25.36 | 27.51 | 25.79 | 26.41 | 26.45 |  |  |
| Q62000 | Ogn | | 0.8 | | 26.45 | | 26.85 | 28.13 | 27.9 | 27.02 | 27.79 | 26.39 | 27.48 | 28.19 |  |  |
| Q9QZ85 | Iigp1 | | 0.63 | | 26.44 | | 25.59 | 23.82 | 26.25 | 24.8 | 20.94 | 24.28 | 25.88 | 25.72 |  |  |
| P56480 | Atp5b | | 0.88 | | 26.42 | | 24.64 | 25.51 | 25.72 | 24.81 | 25.33 | 23.46 | 26.15 | 28.05 |  |  |
| Q3U1V6 | Uevld | | 0.48 | | 26.4 | | 24.83 | 25.12 | 23.51 | 23.2 | 25.33 | 25.29 | 25.09 | 17.5 |  |  |
| P13597 | Icam1 | | 0.58 | | 26.4 | | 24.93 | 17.5 | 26.45 | 25.71 | 23.55 | 26.79 | 26.75 | 22.94 |  |  |
| Q8C3J5 | Dock2 | | 0.8 | | 26.4 | | 25.63 | 25.33 | 25.91 | 26.13 | 24.4 | 25.5 | 26.14 | 24.44 |  |  |
| Q921F2 | Tardbp | | 0.5 | | 26.4 | | 25.59 | 26.63 | 25.25 | 26.29 | 26.26 | 26.25 | 26.64 | 26.37 |  |  |
| P24547 | Impdh2 | | 0.08 | | 26.4 | | 25.96 | 25.49 | 26.25 | 27.64 | 27.14 | 27.59 | 26.73 | 26.87 |  |  |
| O88792 | F11r | | 0.86 | | 26.4 | | 26.24 | 31.01 | 26.86 | 27.02 | 27.4 | 26.44 | 27.09 | 28.79 |  |  |
| Q91VH6 | Memo1 | | 0.07 | | 26.39 | | 25.73 | 26.04 | 24.23 | 25.48 | 25.49 | 26.12 | 26.52 | 25.85 |  |  |
| Q8BXQ8 | Fam53c | | 0.76 | | 26.38 | | 26.6 | 27.25 | 26.55 | 26.58 | 26.53 | 26.67 | 26.08 | 26.89 |  |  |
| Q8R5L1 | C1qbp | | 0.51 | | 26.37 | | 24.89 | 22.84 | 24.75 | 25.5 | 17.5 | 25.66 | 23.9 | 26.23 |  |  |
| Q8BH61 | F13a1 | | 0.98 | | 26.36 | | 26.94 | 27.22 | 26.77 | 26.76 | 27.09 | 26.61 | 26.49 | 27.3 |  |  |
| D3YZ62 | Myo5a | | 0.42 | | 26.35 | | 24.81 | 26.91 | 26.7 | 25.83 | 26.85 | 25.05 | 26.2 | 25.55 |  |  |
| B1B0C7 | Hspg2 | | 0.43 | | 26.35 | | 26.18 | 26.51 | 26.03 | 24.97 | 26.38 | 17.5 | 26.08 | 26.15 |  |  |
| P62141 | Ppp1cb | | 0.82 | | 26.35 | | 25.35 | 25.84 | 25.68 | 25.82 | 25.78 | 25.84 | 24.57 | 26.24 |  |  |
| P29351 | Ptpn6 | | 0.58 | | 26.34 | | 26.83 | 23.93 | 25.7 | 28.44 | 26.27 | 26.3 | 26.13 | 26.47 |  |  |
| A2ALB3 | Adamts13 | | 0.45 | | 26.34 | | 26.97 | 27.29 | 27.47 | 27.21 | 27.36 | 27.3 | 26.7 | 28.34 |  |  |
| Q3UHK6 | Tenm4 | | 0.73 | | 26.32 | | 25.79 | 24.54 | 26.21 | 24.86 | 25.98 | 25.48 | 26.11 | 26.49 |  |  |
| E9Q7P0 | Dnah17 | | 0.45 | | 26.32 | | 26.62 | 26.46 | 26.74 | 26.53 | 26.76 | 26.5 | 26.5 | 29.79 |  |  |
| Q9EPU0 | Upf1 | | 0.84 | | 26.3 | | 25.37 | 25.66 | 24.5 | 26.01 | 26.27 | 25.27 | 26.46 | 24.35 |  |  |
| P84096 | Rhog | | 0.32 | | 26.3 | | 26.61 | 25.17 | 26.99 | 25.74 | 26.64 | 26.12 | 25.71 | 24.42 |  |  |
| Q9WV55 | Vapa | | 0.45 | | 26.3 | | 25.8 | 25.42 | 26.46 | 26.43 | 26.48 | 25.87 | 26.8 | 24.55 |  |  |
| P48962 | Slc25a4 | | 0.55 | | 26.29 | | 25.34 | 25.38 | 25.96 | 25.62 | 25.94 | 26.24 | 26.49 | 25.5 |  |  |
| Q9Z127 | Slc7a5 | | 0.86 | | 26.29 | | 26.84 | 25.47 | 26.23 | 26.1 | 26.76 | 26.13 | 26.3 | 26.1 |  |  |
| G3X8R0 | Reep5 | | 0.47 | | 26.29 | | 25.39 | 25.96 | 25.9 | 26.53 | 25.44 | 26.35 | 26.44 | 26.13 |  |  |
| P30993 | C5ar1 | | 0.33 | | 26.28 | | 25.76 | 27.44 | 23.91 | 17.5 | 26.64 | 26.22 | 26.26 | 23.39 |  |  |
| A8XY17 | Vps25 | | 0.56 | | 26.28 | | 26.19 | 24.63 | 25.95 | 26.19 | 26.17 | 26.08 | 25.86 | 24.18 |  |  |
| G3UXX3 | Spr | | 0.32 | | 26.28 | | 25.95 | 26.28 | 26.16 | 17.5 | 25.15 | 26.24 | 25.83 | 26.59 |  |  |
| Q9DBC7 | Prkar1a | | 0.86 | | 26.28 | | 26.73 | 25.72 | 26.41 | 26.69 | 26.22 | 26.46 | 25.81 | 26.78 |  |  |
| A0A171KXD3 | Prmt1 | | 0.49 | | 26.27 | | 24.85 | 26.98 | 26.16 | 25.69 | 25.7 | 26.4 | 25.93 | 17.5 |  |  |
| E9Q5H2 | Anp32e | | 0.52 | | 26.27 | | 17.5 | 25.9 | 25.78 | 25.49 | 25.83 | 26.28 | 26.55 | 24.39 |  |  |
| Q8K310 | Matr3 | | 0.32 | | 26.27 | | 25.66 | 26.58 | 25.19 | 25.95 | 25.78 | 25.31 | 26.05 | 25.83 |  |  |
| Q8BND5 | Qsox1 | | 1 | | 26.27 | | 25.19 | 26.82 | 26.77 | 25.91 | 25.5 | 26.39 | 25.21 | 26.58 |  |  |
| O89023 | Tpp1 | | 0.47 | | 26.26 | | 26.47 | 26.28 | 24.96 | 26.33 | 26.29 | 26.15 | 26.18 | 26.32 |  |  |
| A0A087WS48 | Bzw1 | | 0.46 | | 26.26 | | 25.58 | 30.09 | 25.68 | 25.29 | 25.53 | 25.88 | 25.92 | 29.37 |  |  |
| Q9R182 | Angptl3 | | 0.9 | | 26.24 | | 25.57 | 26.14 | 26.17 | 26.1 | 25.81 | 26.13 | 25.68 | 26.5 |  |  |
| P63260 | Actg1 | | 0.32 | | 26.23 | | 26.68 | 26.65 | 25.3 | 25.54 | 26.19 | 26.67 | 27.5 | 25.28 |  |  |
| A0A0A6YX26 | Rpl31 | | 0.41 | | 26.23 | | 26.27 | 26.29 | 26.99 | 26.61 | 26.71 | 26.12 | 27.19 | 25.55 |  |  |
| P21550 | Eno3 | | 0.62 | | 26.23 | | 26.24 | 27.38 | 27.37 | 25.9 | 24.46 | 26.97 | 17.5 | 27.3 |  |  |
| P06797 | Ctsl | | 0.66 | | 26.22 | | 17.5 | 28.33 | 24.89 | 28.07 | 26.33 | 26.77 | 26.22 | 25.58 |  |  |
| A2AAN2 | Srp68 | | 0.07 | | 26.22 | | 26.29 | 26.06 | 25.91 | 23.46 | 25.25 | 26.72 | 26.7 | 26.29 |  |  |
| Q3KQM4 | U2af2 | | 0.07 | | 26.21 | | 25.11 | 25.03 | 25.09 | 23.93 | 24.33 | 25.74 | 25.47 | 25.36 |  |  |
| P14211 | Calr | | 0.51 | | 26.21 | | 25.6 | 26.3 | 26.49 | 24.46 | 25.8 | 26.75 | 25.89 | 26.14 |  |  |
| Q9Z1D1 | Eif3g | | 0.28 | | 26.21 | | 25.35 | 24.18 | 25.37 | 25.24 | 17.5 | 26.69 | 26.62 | 26.26 |  |  |
| F8WJG3 | Tra2b | | 0.68 | | 26.2 | | 26.62 | 23.63 | 25.7 | 26.25 | 23.12 | 26.83 | 26.52 | 24.91 |  |  |
| D3YVS7 | Cystm1 | | 0.56 | | 26.2 | | 26.48 | 25.83 | 25.8 | 26.54 | 26.92 | 26.09 | 25.94 | 26.18 |  |  |
| Q8BL97 | Srsf7 | | 0.93 | | 26.19 | | 24.65 | 23.47 | 24.41 | 25.2 | 25.02 | 25.88 | 26.19 | 23.33 |  |  |
| Q6ZWQ9 | Myl12a | | 0.35 | | 26.19 | | 25.58 | 17.5 | 24.66 | 26.72 | 26.27 | 26.83 | 26.85 | 26.25 |  |  |
| G3UY38 | Hnrnpl | | 0.93 | | 26.18 | | 25.97 | 25.72 | 26.61 | 25.67 | 26.01 | 26.2 | 26.9 | 24.46 |  |  |
| J9VKM9 |  | | 0.99 | | 26.18 | | 27.07 | 27.05 | 26.71 | 26.78 | 26.82 | 26.94 | 26.27 | 27.2 |  |  |
| Q922R8 | Pdia6 | | 0.66 | | 26.17 | | 25.44 | 26.44 | 25.98 | 26.66 | 25.76 | 26.74 | 26.18 | 26.16 |  |  |
| E9Q7G1 | Tmed7 | | 0.7 | | 26.16 | | 22.67 | 26.48 | 24.93 | 22 | 25.45 | 25.04 | 26.03 | 24.51 |  |  |
| Q8JZK9 | Hmgcs1 | | 0.89 | | 26.16 | | 24.95 | 26.68 | 26.57 | 26.47 | 25.45 | 26.22 | 26.07 | 25.54 |  |  |
| A0A1D5RM92 | Wwp2 | | 0.97 | | 26.15 | | 26.33 | 23.85 | 26.32 | 24.29 | 25.81 | 26.12 | 26.1 | 24.64 |  |  |
| Q7TMM9 | Tubb2a | | 0.25 | | 26.15 | | 25.79 | 25.83 | 26.21 | 26.22 | 25.94 | 27 | 27.02 | 25.74 |  |  |
| J3QNB3 | Adam17 | | 0.76 | | 26.15 | | 26.33 | 26.38 | 26.68 | 26.11 | 25.07 | 25.91 | 25.85 | 26.62 |  |  |
| Q8R550 | Sh3kbp1 | | 0.08 | | 26.14 | | 24.16 | 25.83 | 26.98 | 26.28 | 27.15 | 26.46 | 26.52 | 26.73 |  |  |
| Q8VH51 | Rbm39 | | 0.96 | | 26.13 | | 25.42 | 23.22 | 26.18 | 25.21 | 24.17 | 24.89 | 26.39 | 24.12 |  |  |
| Q9ESX5 | Dkc1 | | 0.61 | | 26.13 | | 24.48 | 25.23 | 24.78 | 24.92 | 24.92 | 24.48 | 25.5 | 26.59 |  |  |
| O35604 | Npc1 | | 0.24 | | 26.12 | | 25.33 | 24.35 | 25.86 | 25.39 | 26.72 | 17.5 | 26.14 | 22.89 |  |  |
| Q8BU31 | Rap2c | | 0.41 | | 26.12 | | 25.92 | 25.54 | 26.23 | 25.73 | 26.08 | 26.58 | 26.76 | 25.65 |  |  |
| O35286 | Dhx15 | | 0.87 | | 26.12 | | 26.57 | 24.58 | 26.38 | 25.57 | 25.03 | 26.12 | 25.81 | 25.98 |  |  |
| P14152 | Mdh1 | | 0.16 | | 26.12 | | 26.8 | 26.15 | 27.23 | 26.56 | 27.28 | 26.9 | 26.28 | 26.07 |  |  |
| Q8BM72 | Hspa13 | | 0.79 | | 26.11 | | 26.8 | 26.5 | 25.47 | 27.01 | 26.76 | 26.58 | 26.38 | 27.21 |  |  |
| O35103 | Omd | | 0.45 | | 26.1 | | 25.45 | 26.96 | 25.01 | 26.74 | 26.25 | 26.27 | 25.96 | 17.5 |  |  |
| Q9JJI8 | Rpl38 | | 0.56 | | 26.09 | | 25.95 | 25.66 | 26.15 | 17.5 | 26.02 | 25.67 | 25.89 | 23.49 |  |  |
| Q99K94 | Stat1 | | 0.14 | | 26.09 | | 26.39 | 25.36 | 24.18 | 25.48 | 24.03 | 25.88 | 25.75 | 24.41 |  |  |
| F6QTS1 | Cops4 | | 0.6 | | 26.09 | | 26.08 | 24.69 | 26.03 | 26.17 | 25.88 | 26.38 | 25.41 | 24.44 |  |  |
| D3Z645 | Vps29 | | 0.64 | | 26.09 | | 24.93 | 26.71 | 25.79 | 26.13 | 25.66 | 25.48 | 24.32 | 26.25 |  |  |
| B0V2N1 | Ptprs | | 0.32 | | 26.08 | | 25.75 | 26.21 | 25.79 | 25.87 | 26.92 | 25.71 | 23.8 | 26.02 |  |  |
| Q9Z1Z0 | Uso1 | | 0.97 | | 26.07 | | 25.55 | 25.85 | 25.38 | 26.74 | 25.32 | 26.43 | 26.35 | 24.97 |  |  |
| Q8BZN7 | Thrap3 | | 0.88 | | 26.07 | | 26.29 | 25.85 | 25.9 | 26.77 | 26.05 | 26.57 | 26.48 | 25.56 |  |  |
| D6RGM3 | Eml2 | | 0.8 | | 26.07 | | 25.95 | 26.71 | 26.89 | 26.62 | 26.02 | 25.2 | 26.69 | 26.68 |  |  |
| Q921W0 | Chmp1a | | 0.65 | | 26.07 | | 26.76 | 26.47 | 26.3 | 26.69 | 27.07 | 26.24 | 26.43 | 26.79 |  |  |
| Q8CG16 | C1ra | | 0.84 | | 26.07 | | 26.37 | 26.85 | 27.19 | 25.54 | 27.34 | 26.44 | 26.82 | 26.83 |  |  |
| Q8CBD1 | Nrip1 | | 0.77 | | 26.06 | | 24.85 | 26.31 | 25.86 | 25.09 | 25.32 | 25.61 | 25.91 | 25.29 |  |  |
| A0A087WPE4 | Tceb1 | | 0.26 | | 26.06 | | 25.53 | 25.85 | 25.67 | 26.23 | 26.24 | 26.03 | 26.32 | 26.28 |  |  |
| Q9QUJ7 | Acsl4 | | 0.47 | | 26.05 | | 23.86 | 24.87 | 24.54 | 25.99 | 26.43 | 25.3 | 25.04 | 23.27 |  |  |
| H3BJY1 | Syngr2 | | 0.35 | | 26.05 | | 24.59 | 17.5 | 26.01 | 25.34 | 25.37 | 25.56 | 26.03 | 25.53 |  |  |
| E9QKA4 | Srrm1 | | 0.44 | | 26.04 | | 24.16 | 17.5 | 25.32 | 25.19 | 24.59 | 25.39 | 25.55 | 24.51 |  |  |
| D3Z3E8 | Mov10 | | 0.98 | | 26.04 | | 25.98 | 23.55 | 25.27 | 24.33 | 25.78 | 25.69 | 25.35 | 24.69 |  |  |
| G3UZJ4 | Prdx5 | | 0.35 | | 26.04 | | 25.93 | 23.74 | 26.28 | 25.68 | 27.89 | 26.64 | 26.39 | 25.29 |  |  |
| Q3U7R1 | Esyt1 | | 0.9 | | 26.03 | | 26.14 | 24.97 | 25.22 | 26.4 | 26.28 | 26.72 | 27.22 | 24.31 |  |  |
| G3UX26 | Vdac2 | | 0.5 | | 26.02 | | 22.86 | 17.5 | 25.63 | 23.04 | 22.98 | 25.48 | 26.01 | 23.2 |  |  |
| Q9CPN8 | Igf2bp3 | | 0.59 | | 26.02 | | 24.32 | 23.53 | 25.13 | 24.46 | 25.06 | 24.89 | 26.07 | 25.08 |  |  |
| O08529 | Capn2 | | 0.74 | | 26.01 | | 24.57 | 22.84 | 23.86 | 26.19 | 24.7 | 25.19 | 26.42 | 17.5 |  |  |
| E9Q1S3 | Sec23a | | 0.72 | | 26 | | 24.62 | 25.41 | 23.85 | 25.49 | 24.62 | 24.72 | 26.62 | 23.81 |  |  |
| Q8BMN7 | Gbp5 | | 0.66 | | 25.99 | | 25.6 | 17.5 | 25.54 | 25.19 | 24.29 | 25.36 | 24.98 | 23.96 |  |  |
| Q78HU3 | Mvb12a | | 0.5 | | 25.99 | | 24.95 | 24.73 | 26.24 | 20.44 | 25.26 | 25.75 | 26.4 | 25.33 |  |  |
| G3UVV4 | Hk1 | | 0.6 | | 25.98 | | 26.29 | 27.57 | 22.06 | 27.59 | 25.78 | 25.89 | 25.62 | 27.03 |  |  |
| P32883 | Kras | | 0.4 | | 25.97 | | 24.22 | 23.15 | 25.81 | 24.39 | 23.94 | 24.32 | 24.3 | 17.5 |  |  |
| P37217 | Cd69 | | 0.72 | | 25.97 | | 25.24 | 24.16 | 25.98 | 26.07 | 24.67 | 25.59 | 26.19 | 24.96 |  |  |
| P63085 | Mapk1 | | 0.21 | | 25.97 | | 26.7 | 26.27 | 26.4 | 25.74 | 25.14 | 26.87 | 26.62 | 26.1 |  |  |
| Q3U6K9 | Psat1 | | 0.77 | | 25.97 | | 27.18 | 27.57 | 26.95 | 27.5 | 26.47 | 27.17 | 27.08 | 27.46 |  |  |
| Q91VH2 | Snx9 | | 0.28 | | 25.96 | | 25.49 | 24.8 | 23.84 | 24.56 | 25.17 | 25.53 | 25.4 | 24.4 |  |  |
| A0A0B4J1E5 | Uck2 | | 0.15 | | 25.96 | | 24.93 | 25.38 | 26.29 | 26.41 | 26.33 | 25.44 | 26.59 | 25.34 |  |  |
| O35295 | Purb | | 0.55 | | 25.95 | | 25.6 | 17.5 | 24.14 | 25.61 | 25.17 | 26.26 | 26.32 | 24.19 |  |  |
| A0A0J9YUS5 | Eif4g1 | | 0.64 | | 25.95 | | 24.87 | 23.21 | 25.37 | 25.02 | 24.91 | 25.64 | 26.08 | 24.52 |  |  |
| Q9D1Q6 | Erp44 | | 0.07 | | 25.95 | | 26.16 | 26.24 | 26.36 | 26.59 | 26.7 | 25.94 | 26.02 | 26.43 |  |  |
| E9Q3N1 | Slc7a1 | | 0.31 | | 25.94 | | 25.72 | 25.82 | 25.69 | 25.31 | 25.93 | 25.61 | 25.68 | 25.24 |  |  |
| Q5SUF2 | Luc7l3 | | 0.54 | | 25.93 | | 25.22 | 23.25 | 23.79 | 23.81 | 23.58 | 25.16 | 24.47 | 17.5 |  |  |
| Q9JLI2 | Col5a3 | | 0.28 | | 25.93 | | 26.18 | 26.57 | 24.53 | 24.77 | 26.5 | 25.96 | 25.25 | 23.94 |  |  |
| B7ZCL8 | Mpp1 | | 0.47 | | 25.93 | | 25.58 | 24.86 | 23.67 | 25.72 | 24.36 | 24.52 | 26 | 24.72 |  |  |
| G3UXR2 | Fap | | 0.27 | | 25.92 | | 25.3 | 26.71 | 26.41 | 26.66 | 27.08 | 26.69 | 25.96 | 26.35 |  |  |
| Q8BZZ3 | Wwp1 | | 0.61 | | 25.91 | | 25.88 | 25.7 | 25.76 | 25.45 | 26.19 | 24.87 | 25.11 | 26.35 |  |  |
| Q62376 | Snrnp70 | | 0.53 | | 25.9 | | 24.99 | 17.5 | 25.42 | 24.5 | 24.59 | 25.46 | 25.23 | 24.95 |  |  |
| P97499 | Tep1 | | 0.37 | | 25.88 | | 25.16 | 26.12 | 25.88 | 25.85 | 22.17 | 22.64 | 25.72 | 22.71 |  |  |
| A0A0G2JDL3 | Cdk5 | | 0.15 | | 25.88 | | 26.06 | 25.52 | 25.96 | 26.08 | 25.47 | 26.19 | 26.28 | 26.2 |  |  |
| D3YX76 | Gstm2 | | 0.63 | | 25.88 | | 25.85 | 26.54 | 26.6 | 26.39 | 26.02 | 26.03 | 26.3 | 26.62 |  |  |
| P27601 | Gna13 | | 0.44 | | 25.87 | | 22.77 | 17.5 | 24.14 | 24.3 | 25.11 | 26.06 | 25.47 | 22.68 |  |  |
| Q99LL5 | Pwp1 | | 0.62 | | 25.87 | | 24.24 | 24.3 | 23.78 | 23.94 | 25.6 | 25.3 | 23.59 | 22.85 |  |  |
| A0A1D5RM72 | Dmbt1 | | 0.21 | | 25.87 | | 24.6 | 24.74 | 27.14 | 26.07 | 25.31 | 25.84 | 25.57 | 25.51 |  |  |
| P08121 | Col3a1 | | 0.62 | | 25.87 | | 26.28 | 27.07 | 24.89 | 25.65 | 27 | 26.03 | 25.41 | 26.18 |  |  |
| B8JJM9 | C2 | | 0.37 | | 25.87 | | 26.47 | 26.7 | 25.67 | 25.75 | 26.17 | 25.33 | 25.33 | 26.58 |  |  |
| Q3UMB9 | Kiaa1033 | | 0.39 | | 25.86 | | 26.16 | 26.3 | 25.87 | 24.98 | 26.23 | 25.92 | 25.53 | 25.53 |  |  |
| O89079 | Cope | | 0.68 | | 25.86 | | 24.65 | 24.95 | 26.17 | 24.15 | 24.75 | 25.61 | 25.42 | 25.54 |  |  |
| Q04447 | Ckb | | 0.25 | | 25.86 | | 26.78 | 25.79 | 26.33 | 24.45 | 25.78 | 26.46 | 26.65 | 26.35 |  |  |
| S4R1I6 | Ddx5 | | 0.72 | | 25.85 | | 26.04 | 25.49 | 25.87 | 25.04 | 25.97 | 25.81 | 25.9 | 25.82 |  |  |
| G3X922 | Dnajc13 | | 0.36 | | 25.85 | | 25.63 | 25 | 29.87 | 26.05 | 25.77 | 25.28 | 26.74 | 25.83 |  |  |
| Q9WVE8 | Pacsin2 | | 0.35 | | 25.84 | | 25.7 | 24.78 | 26.14 | 26.19 | 26.46 | 26.05 | 26.59 | 24.77 |  |  |
| A0A0N4SVV4 |  | | 0.93 | | 25.84 | | 25.69 | 26.18 | 26.13 | 25.98 | 25.72 | 25.99 | 26.1 | 25.5 |  |  |
| Q8R422 | Cd109 | | 0.29 | | 25.83 | | 25.32 | 25.85 | 24.03 | 25.59 | 24.5 | 25.13 | 24.22 | 17.5 |  |  |
| G3UXY9 | Enpp2 | | 0.69 | | 25.83 | | 26.04 | 27.21 | 25.83 | 25.68 | 27.2 | 25.51 | 25.52 | 26.53 |  |  |
| Q3TFP0 | Srsf10 | | 0.29 | | 25.82 | | 25.13 | 24.13 | 24.61 | 17.5 | 23.55 | 24.25 | 23.91 | 24 |  |  |
| P51942 | Matn1 | | 0.68 | | 25.82 | | 25.93 | 25.17 | 25.59 | 25.33 | 25.62 | 23.87 | 25.82 | 25.76 |  |  |
| Q99KF1 | Tmed9 | | 0.42 | | 25.81 | | 17.5 | 23.66 | 24.82 | 24.6 | 25.51 | 24.96 | 26.43 | 23.49 |  |  |
| A0A0G2JFB4 | Eif4e | | 0.89 | | 25.81 | | 23.37 | 26.3 | 24.42 | 26.68 | 24.25 | 23.86 | 25.63 | 24.59 |  |  |
| Q91Y97 | Aldob | | 0.66 | | 25.81 | | 26.61 | 26.38 | 26.68 | 26.5 | 26.32 | 26.65 | 26.01 | 26.92 |  |  |
| Q9D0F9 | Pgm1 | | 0.85 | | 25.8 | | 27.38 | 27.52 | 27 | 27.15 | 26.87 | 26.53 | 26 | 27.5 |  |  |
| P62748 | Hpcal1 | | 0.4 | | 25.79 | | 23.37 | 23.96 | 25.23 | 24.78 | 25.26 | 25.82 | 25.71 | 24.51 |  |  |
| Q99KJ8 | Dctn2 | | 0.58 | | 25.78 | | 23.37 | 23.24 | 25.53 | 26.72 | 23.53 | 25.76 | 26.35 | 23.73 |  |  |
| Q6DFW4 | Nop58 | | 0.11 | | 25.77 | | 25.82 | 25.75 | 25.05 | 24.23 | 25.29 | 24.28 | 25.63 | 24.51 |  |  |
| P68181 | Prkacb | | 0.25 | | 25.77 | | 26.03 | 26.33 | 26.83 | 26.41 | 26.17 | 26.58 | 26.89 | 26.08 |  |  |
| A2BFF8 | Dync1i2 | | 0.65 | | 25.76 | | 26.51 | 24.27 | 23.83 | 26.45 | 23.29 | 25.68 | 26.28 | 24.07 |  |  |
| A0A1C7CYU3 | Nucb1 | | 0.96 | | 25.76 | | 23.82 | 27.94 | 27.53 | 24.83 | 26.02 | 26.03 | 26.06 | 26.23 |  |  |
| Q91VC3 | Eif4a3 | | 0.77 | | 25.76 | | 26.44 | 26.39 | 26.61 | 26.6 | 26.02 | 25.79 | 26.38 | 26.55 |  |  |
| G3UXL2 | Prps1l3 | | 0.35 | | 25.75 | | 23.96 | 25.44 | 25.89 | 26.22 | 25.72 | 25.89 | 25.42 | 24.2 |  |  |
| Q6GTM0 | Ifit2 | | 0.37 | | 25.74 | | 25.65 | 22.95 | 23.91 | 25.55 | 23.57 | 25.79 | 25.99 | 25.32 |  |  |
| Q9R1Q9 | Atp6ap1 | | 0.86 | | 25.74 | | 25.88 | 26.46 | 25.05 | 25.75 | 26.58 | 25.8 | 25.53 | 26.84 |  |  |
| Q8BJF9 | Chmp2b | | 0.71 | | 25.73 | | 26.25 | 25.34 | 25.07 | 26.27 | 26.69 | 25.42 | 26.02 | 25.36 |  |  |
| P55264 | Adk | | 0.13 | | 25.73 | | 25.94 | 26.29 | 26.62 | 26.55 | 26.18 | 26.22 | 26.01 | 25.56 |  |  |
| Q9JIM1 | Slc29a1 | | 0.2 | | 25.73 | | 24.25 | 25.07 | 25.98 | 24.89 | 26.19 | 25.84 | 26.09 | 26.09 |  |  |
| Q9QXY6 | Ehd3 | | 0.32 | | 25.73 | | 24.85 | 26.71 | 25.6 | 17.5 | 25.26 | 25.49 | 26.7 | 26.48 |  |  |
| D3YX34 | Dctn1 | | 0.81 | | 25.7 | | 25.88 | 23.2 | 25.49 | 26.94 | 24.67 | 26.17 | 26.28 | 23.38 |  |  |
| Q3UPV6 | Kcnab2 | | 0.84 | | 25.68 | | 24.93 | 24.84 | 24.78 | 26.56 | 25.89 | 26.84 | 23.22 | 26.07 |  |  |
| Q6P9R2 | Oxsr1 | | 0.69 | | 25.67 | | 25.54 | 23.17 | 25.35 | 25.76 | 24.76 | 25.6 | 25.56 | 25 |  |  |
| A2AQE4 | Cops2 | | 0.27 | | 25.67 | | 24.32 | 25.1 | 23.99 | 24.19 | 25.38 | 25.27 | 25.64 | 25.25 |  |  |
| B1AXY5 | B4galt1 | | 0.79 | | 25.66 | | 26.05 | 26.73 | 26.21 | 25.9 | 25.78 | 25.94 | 25.8 | 27.03 |  |  |
| Q8VHX6 | Flnc | | 0.49 | | 25.65 | | 24.83 | 26.09 | 26.54 | 26.59 | 25.78 | 26.21 | 26.33 | 24.08 |  |  |
| Q99LU0 | Chmp1b1 | | 0.47 | | 25.65 | | 25.46 | 25.69 | 24.97 | 25.71 | 25.61 | 17.5 | 25.76 | 25.66 |  |  |
| Q9ES97 | Rtn3 | | 0.34 | | 25.65 | | 26.3 | 17.5 | 27.38 | 26.17 | 26.4 | 25.41 | 26.07 | 27.59 |  |  |
| P0DOV1 | Ifi205a | | 0.69 | | 25.61 | | 25.99 | 23.31 | 25.25 | 25.58 | 25.43 | 26.31 | 26.2 | 17.5 |  |  |
| Q5SVG5 | Ap1b1 | | 0.52 | | 25.6 | | 25.18 | 24.91 | 25.23 | 26.68 | 26.11 | 25.88 | 26.01 | 22.16 |  |  |
| Q9CQR4 | Acot13 | | 0.43 | | 25.6 | | 25.33 | 24.36 | 25.72 | 26.03 | 25.22 | 25.59 | 25.5 | 24.87 |  |  |
| Q3U4W8 | Usp5 | | 0.07 | | 25.6 | | 25.43 | 25.59 | 25.92 | 26.58 | 25.5 | 24.95 | 25.39 | 25.23 |  |  |
| Q3TBV5 | Il1rn | | 0.83 | | 25.6 | | 24.95 | 25.22 | 25.91 | 24.38 | 24.31 | 23.94 | 25.42 | 25.73 |  |  |
| D3Z275 | Pcyox1 | | 0.49 | | 25.6 | | 27.03 | 26.33 | 21.72 | 26.67 | 26.01 | 25.94 | 25.82 | 26.86 |  |  |
| Q8CBA2 | Slfn5 | | 0.27 | | 25.59 | | 25.21 | 24.99 | 24.98 | 25.47 | 25.19 | 24.66 | 25.36 | 23.8 |  |  |
| D3YTP0 | Steap3 | | 0.71 | | 25.59 | | 24.98 | 24.13 | 25.01 | 25.14 | 25.32 | 24.84 | 25.35 | 25.39 |  |  |
| E9Q242 | Adsl | | 0.61 | | 25.58 | | 27.16 | 27.35 | 27.23 | 27.42 | 27.01 | 26.69 | 26.63 | 27.4 |  |  |
| P70452 | Stx4 | | 0.35 | | 25.57 | | 24.37 | 24.99 | 24.15 | 17.5 | 24.55 | 24.22 | 24.03 | 23.79 |  |  |
| P57787 | Slc16a3 | | 0.57 | | 25.56 | | 24.08 | 17.5 | 24.8 | 25.04 | 23.3 | 25.25 | 26.33 | 22.55 |  |  |
| Q8VIJ6 | Sfpq | | 0.13 | | 25.56 | | 23.11 | 24.23 | 26.6 | 25.69 | 25.29 | 25.22 | 25.39 | 26.02 |  |  |
| P08030 | Aprt | | 0.48 | | 25.55 | | 26.01 | 26.31 | 26.11 | 26.69 | 26.09 | 26.78 | 26.31 | 25.9 |  |  |
| P32037 | Slc2a3 | | 0.58 | | 25.54 | | 24.88 | 24.7 | 25.35 | 24.55 | 25.63 | 25.33 | 25.36 | 25.53 |  |  |
| P63038 | Hspd1 | | 0.06 | | 25.54 | | 25.8 | 25.98 | 26.7 | 26.68 | 26.03 | 26.41 | 26.63 | 26.06 |  |  |
| Q9EP73 | Cd274 | | 0.43 | | 25.53 | | 25.86 | 24.48 | 25.68 | 24.89 | 17.5 | 25.48 | 25.56 | 24.85 |  |  |
| O35864 | Cops5 | | 0.97 | | 25.53 | | 25.69 | 25.84 | 25.24 | 25.95 | 26.02 | 25.57 | 25.83 | 25.73 |  |  |
| Q8BFZ3 | Actbl2 | | 0.36 | | 25.52 | | 23.95 | 25.68 | 25.59 | 26.73 | 26.35 | 26.12 | 26.59 | 24.36 |  |  |
| P97333 | Nrp1 | | 0.19 | | 25.51 | | 23.46 | 24.97 | 26.17 | 25.2 | 24.71 | 26.07 | 25.88 | 25.91 |  |  |
| Q8BH43 | Wasf2 | | 0.35 | | 25.5 | | 25.65 | 25.56 | 26.96 | 26.1 | 25.05 | 25.81 | 25.1 | 17.5 |  |  |
| Q9EPL8 | Ipo7 | | 0.26 | | 25.5 | | 25.03 | 25.27 | 25.51 | 24.86 | 25.34 | 25.2 | 24.85 | 23.65 |  |  |
| H7BWY2 | Ap3m1 | | 0.74 | | 25.5 | | 23.86 | 24.06 | 24.56 | 24.04 | 23.92 | 25.96 | 24.39 | 23.79 |  |  |
| Q9Z1W8 | Atp12a | | 0.34 | | 25.5 | | 25.41 | 17.5 | 26.01 | 26.41 | 26.02 | 25.25 | 25.36 | 25.24 |  |  |
| P98064 | Masp1 | | 0.94 | | 25.48 | | 28 | 28.12 | 26.91 | 27.1 | 27.35 | 26.53 | 27.26 | 27.02 |  |  |
| B2C3G8 | Stat5a | | 0.37 | | 25.47 | | 25.51 | 25.44 | 25.17 | 24.78 | 24.42 | 24.79 | 25.17 | 17.5 |  |  |
| Q8BFY6 | Pef1 | | 0.62 | | 25.47 | | 24.9 | 24.68 | 24.03 | 25.45 | 25.57 | 25.33 | 24.91 | 22.66 |  |  |
| Q921M3 | Sf3b3 | | 0.73 | | 25.47 | | 26.29 | 25.71 | 25.74 | 26.73 | 25.54 | 25.35 | 25.9 | 25.81 |  |  |
| O35566 | Cd151 | | 0.96 | | 25.47 | | 25.39 | 25.73 | 24.73 | 25.05 | 26.64 | 25.26 | 25.34 | 26.29 |  |  |
| J9VGG9 |  | | 0.49 | | 25.46 | | 17.5 | 26.5 | 25.58 | 25.39 | 26.1 | 25.61 | 25.71 | 25.93 |  |  |
| Q6PHN9 | Rab35 | | 0.53 | | 25.45 | | 17.5 | 25.29 | 24.93 | 25.41 | 24.56 | 25.23 | 25.33 | 24.28 |  |  |
| Q9D7M1 | Gid8 | | 0.67 | | 25.44 | | 24.48 | 26.06 | 24.86 | 26.24 | 25.72 | 25.42 | 25.78 | 23.53 |  |  |
| Q61768 | Kif5b | | 0.97 | | 25.43 | | 25.91 | 24.94 | 24.59 | 26.19 | 25.41 | 25.49 | 25.64 | 25.35 |  |  |
| B2RXS4 | Plxnb2 | | 0.83 | | 25.42 | | 24.24 | 24.05 | 25.77 | 23.81 | 25.31 | 24.82 | 25.84 | 24.14 |  |  |
| Q9CZT5 | Vasn | | 0.26 | | 25.42 | | 25.42 | 26.41 | 26.54 | 26.44 | 26.14 | 26.46 | 25.8 | 27.03 |  |  |
| P45377 | Akr1b8 | | 0.72 | | 25.4 | | 25.45 | 26.49 | 27.14 | 26.62 | 25.33 | 26.49 | 25.01 | 27.25 |  |  |
| E9Q634 | Myo1e | | 0.33 | | 25.39 | | 24.19 | 22.87 | 25.43 | 24.34 | 25.38 | 25.28 | 25.28 | 24.84 |  |  |
| Q8C5G6 | Tollip | | 0.82 | | 25.38 | | 22.82 | 24.41 | 25.05 | 24.01 | 24.58 | 23.93 | 25.23 | 24.73 |  |  |
| A0A2I3BQL9 | Pck2 | | 0.63 | | 25.38 | | 25.32 | 23.03 | 23.52 | 25.85 | 24.36 | 25.61 | 24.79 | 25.56 |  |  |
| P61028 | Rab8b | | 0.06 | | 25.37 | | 25.54 | 25.01 | 25.82 | 25.53 | 25.75 | 26.59 | 26.54 | 25.58 |  |  |
| Q8R2Q8 | Bst2 | | 0.24 | | 25.36 | | 25.4 | 25.69 | 26.22 | 25.13 | 25.28 | 24.82 | 25.32 | 24.83 |  |  |
| D6RFU9 | Sypl | | 0.34 | | 25.35 | | 24.96 | 25.78 | 25.17 | 26.58 | 26.63 | 25.66 | 25.93 | 24.88 |  |  |
| P62315 | Snrpd1 | | 0.61 | | 25.35 | | 24.78 | 23.73 | 25.9 | 21.74 | 24.67 | 25.33 | 25.34 | 25 |  |  |
| B1AT92 | Grb2 | | 0.5 | | 25.34 | | 25.47 | 23.25 | 26.16 | 26.58 | 24.35 | 24.84 | 25.53 | 24.55 |  |  |
| O09010 | Lfng | | 0.56 | | 25.34 | | 25.37 | 26.78 | 17.5 | 26.73 | 26.46 | 26.55 | 26.32 | 25.52 |  |  |
| J9VQN7 |  | | 0.06 | | 25.33 | | 25.17 | 25.68 | 26.02 | 25.72 | 25.16 | 25.02 | 24.93 | 24.76 |  |  |
| P14901 | Hmox1 | | 0.49 | | 25.33 | | 24.03 | 26.6 | 17.5 | 25.28 | 25.74 | 25.46 | 25.47 | 24.96 |  |  |
| P06795 | Abcb1b | | 0.96 | | 25.32 | | 24.72 | 23.67 | 24.49 | 24.4 | 24.61 | 24.89 | 24.49 | 24.43 |  |  |
| O88374 | Bcat2 | | 0.74 | | 25.32 | | 24.85 | 25.07 | 25.93 | 25.86 | 24.27 | 25.53 | 25.07 | 25.78 |  |  |
| Q9DC16 | Ergic1 | | 0.78 | | 25.31 | | 24.6 | 19.19 | 24.96 | 21.98 | 26.24 | 23.39 | 26.02 | 23 |  |  |
| Q9CQI6 | Cotl1 | | 0.52 | | 25.31 | | 26.24 | 25.86 | 26.29 | 26 | 25.51 | 26.4 | 26.26 | 25.87 |  |  |
| A0A0J9YTY0 | 43354 | | 0.1 | | 25.31 | | 26.72 | 25.42 | 25.8 | 25.94 | 25.8 | 26.82 | 26.63 | 26.61 |  |  |
| A0A1W2P869 | Sar1a | | 0.5 | | 25.3 | | 17.5 | 24.1 | 23.46 | 23.38 | 25.67 | 25.08 | 25.08 | 24.14 |  |  |
| P35293 | Rab18 | | 0.73 | | 25.3 | | 22.72 | 24.5 | 24.39 | 26.57 | 17.5 | 25.08 | 23.97 | 24.84 |  |  |
| F6QL70 | Gm17669 | | 0.79 | | 25.29 | | 25.74 | 24.7 | 24.91 | 25.38 | 24.99 | 25.56 | 25.33 | 25 |  |  |
| P19157 | Gstp1 | | 0.5 | | 25.29 | | 25.85 | 28.08 | 27.61 | 27.89 | 26.25 | 27.88 | 27.66 | 26.67 |  |  |
| Q60972 | Rbbp4 | | 0.38 | | 25.28 | | 24.44 | 25.09 | 24.03 | 25.17 | 23.59 | 24.9 | 24.96 | 24.41 |  |  |
| Q9ER41 | Tor1b | | 0.21 | | 25.28 | | 26.27 | 26.1 | 25.77 | 25.78 | 25.96 | 25.48 | 25.02 | 25.56 |  |  |
| P14106 | C1qb | | 0.38 | | 25.28 | | 25.42 | 25 | 25.21 | 17.5 | 24.5 | 23.79 | 25.59 | 25.63 |  |  |
| Q61739 | Itga6 | | 0.35 | | 25.27 | | 22.92 | 22.54 | 24.84 | 24.7 | 24.84 | 24.75 | 25.02 | 23.51 |  |  |
| G5E902 | Slc25a3 | | 0.34 | | 25.27 | | 24.13 | 23.78 | 24.08 | 23.72 | 23.96 | 23.76 | 25.15 | 26.01 |  |  |
| J3QQ16 | Col6a3 | | 0.63 | | 25.26 | | 26.31 | 24.65 | 24.56 | 23.7 | 25.91 | 24.48 | 25.54 | 24.72 |  |  |
| G3UYD5 | Gne | | 0.57 | | 25.26 | | 25.89 | 17.5 | 24.41 | 23.07 | 22.82 | 24.86 | 25.1 | 25.87 |  |  |
| F6QCP8 | Ace | | 0.58 | | 25.26 | | 26.03 | 26.68 | 26.11 | 26.29 | 26.45 | 25.87 | 25.36 | 26.31 |  |  |
| Q8BLF1 | Nceh1 | | 0.36 | | 25.25 | | 17.5 | 24.11 | 25.19 | 23.76 | 24.94 | 25.26 | 25.63 | 24.9 |  |  |
| A6H5X4 | Phf11 | | 0.08 | | 25.22 | | 23.67 | 22.54 | 21.28 | 22.72 | 17.5 | 23.56 | 24.56 | 24.27 |  |  |
| Q3ULG5 | Mcm6 | | 0.81 | | 25.22 | | 25.7 | 17.5 | 25.19 | 24.09 | 21.87 | 24.38 | 23.86 | 24.74 |  |  |
| A0A1W2P701 | Snx6 | | 0.08 | | 25.2 | | 25.4 | 25.42 | 24.67 | 25.54 | 25.32 | 26.24 | 25.63 | 25.8 |  |  |
| D3Z5I1 | Zc3hav1 | | 0.69 | | 25.19 | | 24.17 | 24.61 | 25.67 | 22.31 | 23.74 | 23.38 | 24.74 | 24.14 |  |  |
| F6RV17 | Ppp2r2d | | 0.1 | | 25.19 | | 24.89 | 25.18 | 25.29 | 25.28 | 25.32 | 25.06 | 25.19 | 25.11 |  |  |
| Q9D0F3 | Lman1 | | 0.21 | | 25.19 | | 25.55 | 25.53 | 25.77 | 26 | 27.12 | 25.11 | 26.08 | 26.03 |  |  |
| F2Z456 | Cyb5r3 | | 0.65 | | 25.18 | | 24.12 | 23.52 | 24.44 | 23.57 | 24.71 | 24.84 | 25.05 | 24.2 |  |  |
| P10400 | Pol | | 0.5 | | 25.18 | | 24.37 | 23.36 | 24.95 | 24.43 | 25.23 | 24.71 | 24.89 | 24.61 |  |  |
| Q810B6 | Ankfy1 | | 0.81 | | 25.18 | | 25.5 | 23.33 | 24.49 | 25.92 | 23.99 | 25.65 | 24.88 | 24.9 |  |  |
| D3Z233 | Fam129a | | 0.11 | | 25.17 | | 22.58 | 24.91 | 22.44 | 17.5 | 21.7 | 25.4 | 24.82 | 22.18 |  |  |
| J9VJZ0 |  | | 0.84 | | 25.17 | | 25.01 | 24.53 | 24.24 | 25.02 | 25.13 | 25.69 | 25.45 | 24.09 |  |  |
| A2AS98 | Nckap1 | | 0.31 | | 25.17 | | 26.64 | 25.11 | 25.46 | 25.27 | 24.92 | 25.77 | 25.67 | 28.23 |  |  |
| G5E8J9 | Scyl2 | | 0.5 | | 25.16 | | 22.37 | 24.29 | 23.72 | 21.76 | 22.81 | 23.72 | 23.68 | 17.5 |  |  |
| F7D5L2 | Wdr77 | | 0.28 | | 25.16 | | 25.55 | 24.98 | 25.01 | 25.4 | 25.22 | 24.74 | 24.45 | 17.5 |  |  |
| Q9CQM2 | Kdelr2 | | 0.57 | | 25.16 | | 24.47 | 23.57 | 25.14 | 23.76 | 25.21 | 24.92 | 25.29 | 24.83 |  |  |
| Q64674 | Srm | | 0.23 | | 25.15 | | 25.69 | 25.78 | 25.06 | 25.32 | 25.13 | 25.77 | 25.2 | 26.21 |  |  |
| P06869 | Plau | | 0.69 | | 25.14 | | 23.99 | 20.8 | 24.94 | 23.92 | 23.76 | 25.04 | 23.56 | 23.96 |  |  |
| A2AMH5 | Slc44a1 | | 0.91 | | 25.13 | | 24.46 | 24.55 | 24.98 | 23.58 | 24.79 | 24.73 | 25.51 | 23.14 |  |  |
| Q8CG76 | Akr7a2 | | 0.36 | | 25.13 | | 24.26 | 24.01 | 24.94 | 23.84 | 23.78 | 23.25 | 23.66 | 24.27 |  |  |
| Q9CZ04 | Cops7a | | 0.83 | | 25.11 | | 22.7 | 23.31 | 22.81 | 24.87 | 22.85 | 24.84 | 24.47 | 22.93 |  |  |
| Q80W68 | Kirrel | | 0.92 | | 25.11 | | 27.2 | 25.04 | 25.63 | 24.73 | 25.82 | 25.77 | 23.87 | 27.67 |  |  |
| A0A0A6YX02 | Lamtor1 | | 0.36 | | 25.1 | | 17.5 | 23.39 | 24.57 | 23.62 | 25.22 | 24.31 | 25.36 | 24.37 |  |  |
| Q05816 | Fabp5 | | 0.23 | | 25.09 | | 24.74 | 24.07 | 24.79 | 26.72 | 26.59 | 26.52 | 26.69 | 24.53 |  |  |
| P50396 | Gdi1 | | 0.39 | | 25.09 | | 25.56 | 26.65 | 25.78 | 26.32 | 26.48 | 25.4 | 24.76 | 26.1 |  |  |
| O70503 | Hsd17b12 | | 0.68 | | 25.08 | | 23.65 | 25.73 | 26.32 | 24.82 | 25.14 | 24.75 | 25.74 | 24.62 |  |  |
| Q3UX10 | Tubal3 | | 0.45 | | 25.08 | | 24.62 | 25.39 | 17.5 | 25.32 | 25.22 | 25.2 | 25.08 | 25.51 |  |  |
| O54984 | Asna1 | | 0.62 | | 25.08 | | 24.87 | 27.93 | 25.21 | 27.97 | 27.12 | 22.43 | 26.81 | 26.37 |  |  |
| P01887 | B2m | | 0.69 | | 25.06 | | 26.85 | 25.58 | 26.41 | 24.09 | 24.99 | 24.32 | 25.33 | 26.07 |  |  |
| Q922Q8 | Lrrc59 | | 0.52 | | 25.05 | | 23.64 | 22.88 | 24.58 | 24.4 | 17.5 | 24.71 | 25.26 | 23.54 |  |  |
| Q9WTZ2 | Mbtps1 | | 0.58 | | 25.04 | | 24.45 | 25.03 | 24.24 | 25.45 | 25.45 | 24.5 | 24.09 | 25.12 |  |  |
| Q63836 | Selenbp2 | | 0.24 | | 25.03 | | 25.44 | 25.19 | 24.41 | 24.51 | 23.76 | 23.67 | 24.18 | 25.69 |  |  |
| Q64374 | Rgn | | 0.77 | | 25.02 | | 26.08 | 27.01 | 25.41 | 26.3 | 25 | 26.39 | 25.79 | 24.87 |  |  |
| A0A0G2JDW7 | Rps27 | | 0.36 | | 25.02 | | 25.43 | 17.5 | 24.46 | 26.32 | 25.63 | 25.8 | 25.89 | 25.23 |  |  |
| Z4YK85 | Agrn | | 0.21 | | 25.01 | | 24.87 | 24.33 | 25.32 | 23.3 | 23.62 | 23.34 | 24.14 | 23.14 |  |  |
| Q9ET30 | Tm9sf3 | | 0.45 | | 25.01 | | 24.71 | 25.09 | 25.38 | 25.3 | 25.69 | 17.5 | 25.41 | 25.35 |  |  |
| G3X928 | Sec23ip | | 0.71 | | 25 | | 23.57 | 23.72 | 23.92 | 25.23 | 24.01 | 24.82 | 25.28 | 23.76 |  |  |
| Q9ERN0 | Scamp2 | | 0.33 | | 25 | | 24.71 | 17.5 | 24.2 | 25.6 | 26.27 | 26.04 | 25.11 | 24.73 |  |  |
| Q99MQ4 | Aspn | | 0.86 | | 25 | | 24.9 | 25.76 | 24.26 | 25.3 | 25.5 | 25.18 | 24.44 | 25.37 |  |  |
| G3X920 | Armc8 | | 0.7 | | 24.98 | | 25.85 | 23.88 | 24.59 | 25.35 | 23.46 | 25.38 | 25.48 | 24.32 |  |  |
| P28076 | Psmb9 | | 0.36 | | 24.98 | | 25.17 | 25.36 | 25.31 | 25.19 | 25.11 | 22.76 | 24.67 | 25.46 |  |  |
| Q61166 | Mapre1 | | 0.48 | | 24.98 | | 25.51 | 25.19 | 25.32 | 25.64 | 21.85 | 25.36 | 25.52 | 25.59 |  |  |
| Q04519 | Smpd1 | | 0.37 | | 24.96 | | 24.39 | 25.83 | 25.69 | 25.85 | 25.43 | 25.49 | 25.79 | 25.01 |  |  |
| F8WHM5 | Glg1 | | 0.84 | | 24.95 | | 23.99 | 25.58 | 25.37 | 23.36 | 26.12 | 24.75 | 26.04 | 25.12 |  |  |
| E9PYT3 | Atl3 | | 0.45 | | 24.94 | | 24.97 | 22.88 | 24.52 | 23.41 | 25.53 | 25.14 | 25.43 | 25.16 |  |  |
| A0A0R4IZW8 | Capns1 | | 0.76 | | 24.93 | | 24.18 | 23.76 | 21.97 | 25.4 | 24.45 | 24.28 | 25.42 | 24.22 |  |  |
| Q52KC3 | Mcm5 | | 0.21 | | 24.93 | | 24.21 | 24.88 | 26.28 | 25.77 | 23.89 | 25.81 | 26.28 | 25.79 |  |  |
| P46460 | Nsf | | 0.38 | | 24.88 | | 25.89 | 17.5 | 26.81 | 26.86 | 24.48 | 25.6 | 26.4 | 24.07 |  |  |
| E9QKV6 | Myo9b | | 0.08 | | 24.88 | | 24.39 | 24.35 | 24 | 23.95 | 23.21 | 24.21 | 24.33 | 25.14 |  |  |
| Q3U816 | Htatip2 | | 0.45 | | 24.88 | | 25.89 | 25.26 | 26.01 | 17.5 | 24.75 | 24.8 | 25.79 | 25.21 |  |  |
| Q9EQF5 | Dpys | | 0.55 | | 24.87 | | 24.91 | 24.92 | 24.87 | 23.94 | 24.56 | 24.29 | 24.6 | 25.93 |  |  |
| P28474 | Adh5 | | 0.39 | | 24.85 | | 23.51 | 25.21 | 25.37 | 25.66 | 24.55 | 24.86 | 25.47 | 25.26 |  |  |
| Q6ZWX6 | Eif2s1 | | 0.55 | | 24.84 | | 26.22 | 25.63 | 25.5 | 26.36 | 25.34 | 25.07 | 25.69 | 24.89 |  |  |
| H3BLB7 | Igfbp4 | | 0.88 | | 24.84 | | 25.53 | 26.59 | 26.11 | 25.63 | 25.63 | 25.74 | 24.8 | 26.01 |  |  |
| D3YYN7 | Atp1a2 | | 0.5 | | 24.82 | | 24.21 | 24.65 | 25.33 | 24.64 | 24.82 | 24.92 | 25.14 | 17.5 |  |  |
| A2AR26 | Slc2a6 | | 0.94 | | 24.8 | | 23.26 | 24.34 | 23.58 | 23.48 | 24.58 | 25.2 | 25.01 | 22.3 |  |  |
| P61089 | Ube2n | | 0.79 | | 24.77 | | 25.72 | 25.16 | 25.12 | 25.18 | 25.73 | 25.22 | 25.66 | 25.37 |  |  |
| Q8BRF7 | Scfd1 | | 0.89 | | 24.76 | | 24.55 | 24.31 | 24.89 | 24.93 | 23.05 | 24.82 | 24.86 | 23.86 |  |  |
| Q9QZZ6 | Dpt | | 0.64 | | 24.75 | | 24.81 | 26.43 | 25.74 | 26.01 | 17.5 | 24.53 | 24.63 | 24.06 |  |  |
| A0A0J9YKD4 | Ckm | | 0.19 | | 24.74 | | 24.88 | 25.4 | 25.3 | 24.7 | 24.95 | 25.05 | 26.35 | 25.64 |  |  |
| P0DOV2 | Ifi204 | | 0.7 | | 24.73 | | 21.75 | 19.25 | 25.2 | 17.5 | 21.22 | 25.1 | 24.39 | 20.7 |  |  |
| P06745 | Gpi | | 0.83 | | 24.73 | | 23.31 | 25.75 | 25.29 | 23.57 | 24.54 | 25.5 | 24.66 | 24.59 |  |  |
| P26040 | Ezr | | 0.57 | | 24.73 | | 25.5 | 25.03 | 24.66 | 24.89 | 25.25 | 24.86 | 24.4 | 25.06 |  |  |
| A0A0N4SVU1 | Mug1 | | 0.5 | | 24.73 | | 25.1 | 25.86 | 26.11 | 25.29 | 25.2 | 24.85 | 24.96 | 25.39 |  |  |
| Q543K9 | Pnp | | 0.41 | | 24.72 | | 24.76 | 17.5 | 25.05 | 25.36 | 21.93 | 25.46 | 25.53 | 25.21 |  |  |
| Q9Z0M6 | Cd97 | | 0.41 | | 24.71 | | 22.89 | 24.52 | 24.75 | 24.75 | 24.89 | 23.79 | 24.81 | 24.54 |  |  |
| Q5EBP8 | Hnrnpa1 | | 0.56 | | 24.7 | | 25.46 | 23.87 | 25.01 | 24.83 | 24.44 | 25.59 | 24.78 | 17.5 |  |  |
| Q91XH6 | Vti1b | | 0.35 | | 24.69 | | 23.23 | 17.5 | 23.74 | 25.13 | 25.07 | 23.04 | 24.45 | 24.3 |  |  |
| Q9WVJ3 | Cpq | | 0.73 | | 24.67 | | 25.42 | 25.69 | 25.47 | 24.68 | 25.64 | 25.71 | 24.78 | 26.39 |  |  |
| Q8BJY1 | Psmd5 | | 0.17 | | 24.67 | | 24.85 | 25.82 | 23.89 | 25.34 | 24.24 | 25.53 | 25.19 | 26.64 |  |  |
| G3X956 | Supt16 | | 0.6 | | 24.66 | | 22.76 | 24.33 | 24.69 | 24.62 | 17.5 | 24.91 | 25.41 | 22.86 |  |  |
| A0A1D5RLY2 | Vac14 | | 0.97 | | 24.65 | | 25.14 | 24.05 | 24.31 | 25.14 | 24.11 | 25.16 | 24.68 | 23.65 |  |  |
| Q8CFI0 | Nedd4l | | 0.81 | | 24.65 | | 27.03 | 22.41 | 23.1 | 24.6 | 23.27 | 23.09 | 22.61 | 26.82 |  |  |
| E9Q0X4 | Col16a1 | | 0.34 | | 24.64 | | 25.12 | 26.04 | 24.3 | 23.73 | 25.02 | 24.49 | 25.72 | 24.39 |  |  |
| Q8K0C9 | Gmds | | 0.42 | | 24.64 | | 25.35 | 25.45 | 17.5 | 25.69 | 24.62 | 25.03 | 25.01 | 25.53 |  |  |
| Q8BGX0 | Trim23 | | 0.94 | | 24.57 | | 24.38 | 24.23 | 25 | 24.04 | 24.44 | 24.88 | 23.68 | 25.04 |  |  |
| Q80WM4 | Hapln4 | | 0.22 | | 24.54 | | 22.56 | 22.54 | 24.5 | 24.16 | 24.64 | 23.31 | 24.58 | 24.25 |  |  |
| P67984 | Rpl22 | | 0.15 | | 24.54 | | 23.65 | 25.15 | 23.47 | 24.1 | 23.18 | 24.38 | 24.53 | 24.28 |  |  |
| Q9CQ88 | Tspan31 | | 0.32 | | 24.51 | | 23.88 | 23.18 | 23.47 | 23.09 | 17.5 | 23.45 | 24.41 | 22.84 |  |  |
| P25911 | Lyn | | 0.5 | | 24.5 | | 25.5 | 24.46 | 24.18 | 24.65 | 24.34 | 24.79 | 25.48 | 24.25 |  |  |
| Q8K094 | Pvr | | 0.48 | | 24.5 | | 24.84 | 24.32 | 24.72 | 24.6 | 17.5 | 24.21 | 24.18 | 24.57 |  |  |
| A2AFP4 | Rab9 | | 0.31 | | 24.45 | | 17.5 | 24.14 | 25.48 | 24.96 | 24.89 | 24.34 | 23.99 | 23.55 |  |  |
| F8VPN4 | Agl | | 0.64 | | 24.42 | | 25.16 | 24.24 | 24.29 | 25.45 | 24.75 | 24.8 | 25.18 | 24.91 |  |  |
| Q8R5F7 | Ifih1 | | 0.41 | | 24.41 | | 22.56 | 17.5 | 23.68 | 23.61 | 25.52 | 24.69 | 24.43 | 21.16 |  |  |
| F6QYF8 | Npepps | | 0.98 | | 24.4 | | 24.34 | 25.54 | 24.59 | 25.36 | 24.11 | 25.45 | 24.25 | 24.53 |  |  |
| Q91YQ5 | Rpn1 | | 0.58 | | 24.39 | | 22.61 | 23.63 | 23.18 | 22.5 | 24.75 | 23.13 | 24.53 | 17.5 |  |  |
| Q9QUR8 | Sema7a | | 0.42 | | 24.39 | | 25.6 | 26.81 | 26.42 | 27.82 | 26.55 | 27.07 | 27.23 | 24.72 |  |  |
| A0A0G2JEC4 | Sh3glb1 | | 0.6 | | 24.38 | | 24.2 | 25.28 | 25.27 | 25.47 | 17.5 | 24.7 | 26.31 | 23.78 |  |  |
| E0CY49 | Asl | | 0.93 | | 24.38 | | 25 | 26.41 | 25.43 | 26.52 | 24.11 | 25.68 | 24.11 | 25.38 |  |  |
| Q3UNN4 | Smarcc1 | | 0.78 | | 24.37 | | 23.35 | 21.87 | 26.4 | 23.1 | 22.88 | 23.13 | 25.16 | 22.15 |  |  |
| H3BK73 | Tor2a | | 0.78 | | 24.37 | | 24.6 | 24.17 | 24.76 | 24.17 | 24.27 | 24.32 | 24.43 | 24.83 |  |  |
| F7CBP1 | Eif4g2 | | 0.91 | | 24.35 | | 23.54 | 23.32 | 24.06 | 23.77 | 24.07 | 24.19 | 24.62 | 22.45 |  |  |
| Q8K297 | Colgalt1 | | 0.08 | | 24.35 | | 24.31 | 24.98 | 23.75 | 24.26 | 23.12 | 24.98 | 24.94 | 24.19 |  |  |
| Q8R1F1 | Fam129b | | 0.15 | | 24.35 | | 24.35 | 25.07 | 26.02 | 24.42 | 25.22 | 26.01 | 25.57 | 25.3 |  |  |
| Q62422 | Ostf1 | | 0.23 | | 24.32 | | 23.84 | 23.28 | 24.4 | 25.83 | 25.83 | 26 | 26.14 | 23.33 |  |  |
| P31324 | Prkar2b | | 0.14 | | 24.32 | | 24.95 | 25.92 | 26.52 | 26.86 | 26.84 | 26.56 | 24.35 | 26.52 |  |  |
| P24270 | Cat | | 0.58 | | 24.31 | | 25.02 | 24.13 | 25.31 | 25.48 | 24.12 | 25.6 | 25.06 | 17.5 |  |  |
| D6RHS6 | Pebp1 | | 0.58 | | 24.31 | | 17.5 | 24.47 | 22.88 | 24.26 | 24.04 | 24.21 | 24.33 | 23.56 |  |  |
| A0A0N4SV15 | Kcmf1 | | 0.85 | | 24.3 | | 24.37 | 24.89 | 24.58 | 24.68 | 23.96 | 24.55 | 24.21 | 24.41 |  |  |
| P30204 | Msr1 | | 0.88 | | 24.27 | | 24.72 | 24.59 | 26.35 | 25.42 | 21.76 | 26.32 | 26.22 | 17.5 |  |  |
| O09172 | Gclm | | 0.57 | | 24.25 | | 28.3 | 27.88 | 17.5 | 28.88 | 26.59 | 24.12 | 22.92 | 23.88 |  |  |
| P51807 | Dynlt1 | | 0.63 | | 24.25 | | 24.14 | 24.68 | 25.27 | 25.66 | 17.5 | 24.57 | 25.34 | 24.71 |  |  |
| Q61792 | Lasp1 | | 0.59 | | 24.24 | | 23.74 | 20.93 | 23.77 | 24.19 | 23.91 | 23.79 | 24.27 | 17.5 |  |  |
| E9Q5B6 | Hnrnpd | | 0.76 | | 24.23 | | 24.95 | 25.06 | 25.44 | 24.25 | 24.36 | 26.22 | 25.78 | 17.5 |  |  |
| Q9CYG7 | Tomm34 | | 0.34 | | 24.23 | | 22.98 | 17.5 | 24.14 | 24.59 | 24.12 | 23.13 | 24.51 | 22.89 |  |  |
| Q8R307 | Vps18 | | 0.88 | | 24.23 | | 24.71 | 24.23 | 26.01 | 24.56 | 23.46 | 25.32 | 24.24 | 23.41 |  |  |
| D3YY94 | Stx2 | | 0.48 | | 24.21 | | 23.84 | 23.95 | 24.47 | 17.5 | 24.36 | 23.54 | 25.05 | 24.78 |  |  |
| P83887 | Tubg1 | | 0.77 | | 24.18 | | 23.38 | 26.31 | 24.42 | 25.37 | 25.66 | 25.86 | 24.41 | 25.22 |  |  |
| Q9CR86 | Carhsp1 | | 0.19 | | 24.17 | | 23.57 | 23.89 | 24.03 | 24.06 | 24.08 | 24.16 | 24.22 | 24.16 |  |  |
| Q8BTU6 | Eif4a2 | | 0.21 | | 24.17 | | 24.39 | 24.2 | 23.95 | 22.57 | 17.5 | 23.61 | 23.95 | 24.43 |  |  |
| A0A0R4J150 | Osbpl8 | | 0.5 | | 24.14 | | 23.61 | 17.5 | 23.93 | 23.54 | 23.74 | 24.22 | 24.52 | 22.39 |  |  |
| Q9ER38 | Tor3a | | 0.08 | | 24.13 | | 25.1 | 24.02 | 26.15 | 25.09 | 25.54 | 25.59 | 24.94 | 26.16 |  |  |
| D3Z0P3 | Serpina11 | | 0.74 | | 24.12 | | 24.06 | 24.82 | 23.86 | 23.87 | 24.56 | 24.17 | 24.02 | 24.65 |  |  |
| G3XA25 | Acat2 | | 0.18 | | 24.12 | | 23.51 | 23.95 | 24.36 | 24.31 | 24.18 | 24.19 | 24.09 | 24.93 |  |  |
| E9PWE9 | Syk | | 0.8 | | 24.11 | | 25.04 | 24.05 | 25.09 | 26.06 | 17.5 | 24.35 | 23.63 | 22.57 |  |  |
| F2Z4A3 | Fat1 | | 0.1 | | 24.1 | | 22.2 | 17.5 | 24.75 | 25.32 | 24.98 | 25.57 | 23.97 | 25.45 |  |  |
| F8VQ05 | Fryl | | 0.22 | | 24.09 | | 24.01 | 24.38 | 23.44 | 23.8 | 24.09 | 24.19 | 24.05 | 23.82 |  |  |
| A2A7S7 | Yars | | 0.21 | | 24.09 | | 22.31 | 17.5 | 24.29 | 23.91 | 23.83 | 23.63 | 24.13 | 25 |  |  |
| A2AC16 | Dcxr | | 0.38 | | 24.09 | | 25.09 | 17.5 | 24.67 | 24.52 | 25.17 | 24.73 | 24.29 | 25.23 |  |  |
| Q6P9Q6 | Fkbp15 | | 0.38 | | 24.07 | | 25.71 | 26.12 | 30.6 | 25.82 | 24.44 | 24.38 | 25.5 | 23.66 |  |  |
| P48678 | Lmna | | 0.4 | | 24.07 | | 24.38 | 21.07 | 24.05 | 24.03 | 23.93 | 24.75 | 24.31 | 24.22 |  |  |
| D3Z158 | Qars | | 0.38 | | 24.06 | | 23.82 | 25.09 | 25.63 | 26.67 | 24.46 | 25.25 | 26.08 | 23.63 |  |  |
| Q8VED9 | Lgalsl | | 0.17 | | 24.05 | | 24.16 | 24.55 | 23.58 | 23.63 | 17.5 | 24.93 | 24.97 | 25.08 |  |  |
| Q9ERD7 | Tubb3 | | 0.4 | | 24.05 | | 25.82 | 17.5 | 25.67 | 24.01 | 25.07 | 25.54 | 24.42 | 26.25 |  |  |
| Q91VR5 | Ddx1 | | 0.53 | | 24.03 | | 24.72 | 22.44 | 23.91 | 24.52 | 17.5 | 24.72 | 24.91 | 22.98 |  |  |
| D6RFN5 | Ninj1 | | 0.56 | | 24.01 | | 24.78 | 22.7 | 23.7 | 25.26 | 17.5 | 24.34 | 24.64 | 23.93 |  |  |
| Q6PDS3 | Sarm1 | | 0.12 | | 23.97 | | 23.42 | 23.22 | 21.72 | 23.76 | 22.83 | 22.63 | 21.83 | 22.05 |  |  |
| A0A0R4J0G4 | Ranbp10 | | 0.49 | | 23.92 | | 24.41 | 22.83 | 23.62 | 24.18 | 17.5 | 23.51 | 24.53 | 23.28 |  |  |
| Q00493 | Cpe | | 0.63 | | 23.92 | | 23.21 | 23.73 | 23.3 | 23.87 | 24.01 | 23.99 | 23.56 | 24.15 |  |  |
| Q9EQ06 | Hsd17b11 | | 0.4 | | 23.91 | | 23.62 | 17.5 | 23.82 | 24.16 | 24.11 | 23 | 24.19 | 23.72 |  |  |
| P01027 | C3 | | 0.94 | | 23.89 | | 26.11 | 24.83 | 25.95 | 24.66 | 24.28 | 25.63 | 24.5 | 24.04 |  |  |
| A0A0N4SUM7 | Tmem176b | | 0.4 | | 23.88 | | 23.29 | 22.36 | 22.55 | 22.8 | 23.37 | 22.67 | 23.22 | 17.5 |  |  |
| Q9CQ10 | Chmp3 | | 0.64 | | 23.88 | | 23.59 | 26.23 | 23.64 | 23.61 | 24.79 | 24.26 | 24 | 23.22 |  |  |
| Q3MIA8 | Gps1 | | 0.47 | | 23.88 | | 25.15 | 24.82 | 24.31 | 25.56 | 17.5 | 25.31 | 25.21 | 24.39 |  |  |
| Q7TQG5 | Neo1 | | 0.3 | | 23.85 | | 24.74 | 17.5 | 24.3 | 24.29 | 24.75 | 24.65 | 24.91 | 25.84 |  |  |
| B7ZWL1 | Cnot1 | | 0.05 | | 23.83 | | 23.66 | 22.18 | 22.73 | 21.22 | 20.21 | 23.17 | 24.35 | 23.67 |  |  |
| J9VP10 |  | | 0.38 | | 23.81 | | 24.63 | 25.18 | 17.5 | 23.57 | 24.52 | 24.13 | 23.12 | 24.16 |  |  |
| Q80X90 | Flnb | | 0.53 | | 23.81 | | 24.84 | 25.16 | 25.35 | 25.77 | 22.88 | 25.89 | 25.59 | 25.01 |  |  |
| E9Q1T9 | Cse1l | | 0.44 | | 23.8 | | 23.07 | 24.72 | 23.94 | 24.86 | 24.83 | 25.04 | 22.69 | 22.67 |  |  |
| Q04736 | Yes1 | | 0.36 | | 23.8 | | 25.29 | 21.39 | 25.74 | 25.23 | 24.15 | 23.82 | 25.43 | 25.21 |  |  |
| Q8BYA0 | Tbcd | | 0.63 | | 23.8 | | 25.19 | 25.26 | 24.62 | 25.23 | 23.92 | 24.99 | 24.72 | 25.61 |  |  |
| A0A0N4SUH4 | Kiaa0020 | | 0.68 | | 23.75 | | 23.82 | 23.46 | 23.43 | 21.73 | 24.12 | 23.51 | 23.82 | 22.71 |  |  |
| J3QN31 | Adssl1 | | 0.49 | | 23.72 | | 23.06 | 23.66 | 23.24 | 24.64 | 23.25 | 25.29 | 24.61 | 23.04 |  |  |
| Q6PGC1 | Dhx29 | | 0.61 | | 23.7 | | 22.67 | 23.15 | 22.92 | 22.31 | 23.39 | 23.58 | 22.4 | 21.83 |  |  |
| P40336 | Vps26a | | 0.45 | | 23.67 | | 23.92 | 21.96 | 24.31 | 23.8 | 23.85 | 22.47 | 23.82 | 23.77 |  |  |
| A0A140LHY2 | Prcp | | 0.79 | | 23.67 | | 24.24 | 24.75 | 24.15 | 24.36 | 25.03 | 24.52 | 23.71 | 24.78 |  |  |
| Q9R1S8 | Capn7 | | 0.47 | | 23.66 | | 24.56 | 24.19 | 23.89 | 23.78 | 25.03 | 23.74 | 17.5 | 24.83 |  |  |
| Q921V5 | Mgat2 | | 0.32 | | 23.63 | | 25.12 | 17.5 | 25.49 | 24.8 | 25 | 24.09 | 24.12 | 25.01 |  |  |
| Q6PDI5 | Ecm29 | | 0.64 | | 23.62 | | 23.58 | 22.39 | 24.21 | 23.5 | 23.19 | 23.86 | 23.39 | 23.06 |  |  |
| P97310 | Mcm2 | | 0.71 | | 23.61 | | 23.95 | 23.43 | 25.47 | 23.36 | 23.56 | 24.03 | 24 | 23.91 |  |  |
| Q8K1X4 | Nckap1l | | 0.99 | | 23.59 | | 27.27 | 21.4 | 24.87 | 25.58 | 21.19 | 25.13 | 24.67 | 21.91 |  |  |
| Q9D819 | Ppa1 | | 0.31 | | 23.59 | | 22.77 | 23.71 | 23.96 | 23.99 | 23.34 | 23.58 | 23.22 | 22.61 |  |  |
| Q9CQA1 | Trappc5 | | 0.22 | | 23.57 | | 23.64 | 23.74 | 24.1 | 23.58 | 23.68 | 24.11 | 22.14 | 21.59 |  |  |
| Q5SVI9 | Camk2b | | 0.41 | | 23.56 | | 25.54 | 23.67 | 23.94 | 23.76 | 17.5 | 23.79 | 23.26 | 23.43 |  |  |
| Q6NV83 | U2surp | | 0.46 | | 23.54 | | 23.51 | 22.68 | 23.69 | 20.97 | 22.18 | 23.37 | 22.84 | 22.35 |  |  |
| Q60865 | Caprin1 | | 0.22 | | 23.54 | | 23.81 | 17.5 | 23.77 | 23.38 | 23.67 | 25.4 | 25.05 | 24.44 |  |  |
| A0A1Y7VL93 | Ralb | | 0.64 | | 23.53 | | 17.5 | 28.07 | 25.57 | 24.6 | 24.48 | 25.13 | 25.55 | 25.32 |  |  |
| A0A2I3BRV9 | Gnl3 | | 0.35 | | 23.52 | | 21.88 | 17.5 | 23.7 | 22.89 | 23.43 | 22.59 | 22.22 | 21.92 |  |  |
| Q64345 | Ifit3 | | 0.7 | | 23.51 | | 22.77 | 23.27 | 23.66 | 24.09 | 22.95 | 24.43 | 24.1 | 17.5 |  |  |
| A0A1L1STF4 | Islr | | 0.37 | | 23.49 | | 23.42 | 23.62 | 17.5 | 22.9 | 23.78 | 23.95 | 23.13 | 23.48 |  |  |
| Q5MJS3 | Fam20c | | 0.66 | | 23.49 | | 25.32 | 26 | 23.8 | 25.13 | 23.77 | 25.19 | 24.71 | 24.06 |  |  |
| Q9Z1G3 | Atp6v1c1 | | 0.28 | | 23.47 | | 24.12 | 23.67 | 24.87 | 24.7 | 24.3 | 25.14 | 24.39 | 23.25 |  |  |
| Q4VA93 | Prkca | | 0.25 | | 23.44 | | 23.23 | 22.53 | 17.5 | 21.29 | 23.35 | 22.76 | 22.85 | 23.11 |  |  |
| Q9D2Y4 | Mlkl | | 0.69 | | 23.44 | | 22.97 | 24.83 | 24.89 | 24.52 | 17.5 | 24.46 | 23.96 | 23.44 |  |  |
| Q8VI75 | Ipo4 | | 0.63 | | 23.42 | | 22.51 | 21.91 | 23.77 | 22.07 | 21.26 | 23.14 | 24.36 | 22.17 |  |  |
| Q9D6F9 | Tubb4a | | 0.54 | | 23.42 | | 17.5 | 24.19 | 22.75 | 23.93 | 23.76 | 23.37 | 23.51 | 23.34 |  |  |
| Q99LT0 | Dpy30 | | 0.94 | | 23.37 | | 22.24 | 23.55 | 22.04 | 23.99 | 23.68 | 22.94 | 23.77 | 22.32 |  |  |
| Q9R001 | Adamts5 | | 0.48 | | 23.37 | | 24.99 | 23.84 | 24.1 | 23.84 | 22.68 | 24.15 | 23 | 22.53 |  |  |
| Q6IR41 | C1qtnf6 | | 0.76 | | 23.34 | | 23.93 | 24.41 | 23.56 | 21.75 | 24.75 | 23.62 | 24.16 | 23.59 |  |  |
| O88325 | Naglu | | 0.52 | | 23.33 | | 21.01 | 23.83 | 24.19 | 23.71 | 23.22 | 22.65 | 22.84 | 24.05 |  |  |
| A0A0R4J2B2 | Kctd12 | | 0.34 | | 23.25 | | 23.44 | 17.5 | 22.54 | 24.12 | 23.27 | 25.46 | 24.79 | 22.45 |  |  |
| A0A2I3BPM1 | Ero1l | | 0.98 | | 23.25 | | 25.03 | 23.79 | 24.81 | 23.87 | 23.64 | 25.29 | 23.05 | 24.09 |  |  |
| P97390 | Vps45 | | 0.35 | | 23.22 | | 17.5 | 22 | 23.28 | 22.51 | 22.69 | 22.98 | 22.74 | 22.97 |  |  |
| B1AXN9 | Rps6ka3 | | 0.92 | | 23.22 | | 29.21 | 24.57 | 25.84 | 27.36 | 25.65 | 25.2 | 25.24 | 28.43 |  |  |
| P54276 | Msh6 | | 0.57 | | 23.21 | | 23.75 | 23.28 | 24.01 | 23.3 | 23.63 | 23.85 | 23.6 | 23.46 |  |  |
| E9Q0Q3 | 2610021A01Rik | | 0.99 | | 23.2 | | 23.23 | 22.14 | 22.97 | 22.18 | 23.25 | 22.52 | 23.18 | 22.72 |  |  |
| Q9D662 | Sec23b | | 0.07 | | 23.19 | | 25.14 | 22.57 | 24.08 | 24.82 | 24.31 | 25.93 | 25.11 | 25.81 |  |  |
| A0A087WRV4 | Srgap2 | | 0.28 | | 23.18 | | 22.8 | 23.29 | 24.41 | 23.24 | 23.53 | 24.65 | 23.02 | 24.14 |  |  |
| P97315 | Csrp1 | | 0.83 | | 23.17 | | 25.6 | 22.32 | 24.44 | 25.08 | 23.27 | 23.12 | 25.06 | 22.78 |  |  |
| Q9WV54 | Asah1 | | 0.43 | | 23.15 | | 25.19 | 24.72 | 17.5 | 24.49 | 23.69 | 23.42 | 23.49 | 25.61 |  |  |
| K4DI77 | Wdr81 | | 0.42 | | 23.06 | | 25.65 | 21.95 | 23.46 | 22.92 | 23.48 | 22.84 | 23.41 | 17.5 |  |  |
| Q924C1 | Xpo5 | | 0.25 | | 23.06 | | 22.55 | 25.42 | 24.6 | 25.13 | 24.77 | 25.33 | 24.68 | 24.82 |  |  |
| E0CZ22 | Mroh1 | | 0.08 | | 23.04 | | 21.66 | 22.99 | 23.41 | 24.86 | 23.35 | 21.85 | 22.92 | 21.11 |  |  |
| Q8R4G6 | Mgat5 | | 0.49 | | 23.03 | | 23.88 | 24.1 | 24.27 | 23.65 | 24.59 | 23.81 | 23.8 | 24.9 |  |  |
| P63028 | Tpt1 | | 0.32 | | 23.02 | | 24.15 | 21.79 | 17.5 | 25.04 | 23.61 | 25.73 | 25.27 | 24.81 |  |  |
| Q91XV3 | Basp1 | | 0.67 | | 22.99 | | 23.75 | 20.99 | 23.14 | 23.48 | 23.11 | 23.44 | 23.84 | 17.5 |  |  |
| A2AFQ0 | Huwe1 | | 0.11 | | 22.97 | | 23.47 | 23.09 | 23.47 | 22.99 | 23.09 | 24.08 | 23.25 | 24.07 |  |  |
| Q62418 | Dbnl | | 0.36 | | 22.96 | | 23.16 | 22.67 | 22.6 | 17.5 | 22.28 | 23.25 | 23.31 | 21.1 |  |  |
| Q9CXY6 | Ilf2 | | 0.1 | | 22.95 | | 23.05 | 23.15 | 23.13 | 23.39 | 23.36 | 23.41 | 23.45 | 23.13 |  |  |
| H3BKW0 | Cpsf6 | | 0.2 | | 22.94 | | 23.82 | 22.91 | 22.9 | 21.05 | 22.5 | 22.52 | 21.48 | 17.5 |  |  |
| Q61164 | Ctcf | | 0.78 | | 22.9 | | 23.9 | 17.5 | 18.47 | 22.16 | 22.68 | 22.11 | 23.33 | 22 |  |  |
| P41241 | Csk | | 0.7 | | 22.81 | | 22.48 | 22.75 | 22.97 | 22.12 | 22.46 | 22.27 | 22.88 | 22.15 |  |  |
| O08738 | Casp6 | | 0.22 | | 22.81 | | 21.3 | 22.67 | 23.41 | 23.15 | 22.76 | 22.7 | 22.79 | 22.95 |  |  |
| F6WV69 | Git2 | | 0.69 | | 22.79 | | 20.47 | 23.68 | 21.8 | 22.94 | 23.47 | 20.86 | 21.83 | 22.86 |  |  |
| A0A1L1SVK0 | Pafah1b2 | | 0.16 | | 22.79 | | 22.55 | 22.78 | 23.96 | 23.23 | 22.91 | 22.97 | 23.15 | 23.71 |  |  |
| A2AH25 | Arhgap1 | | 0.96 | | 22.79 | | 24.87 | 24.32 | 22.61 | 24.45 | 24.56 | 22.27 | 24.79 | 24.12 |  |  |
| A0A1D5RLG3 | Rab3gap1 | | 0.63 | | 22.76 | | 25.14 | 25 | 24.65 | 20.85 | 23.61 | 23.44 | 22.88 | 25.66 |  |  |
| D3Z494 | Akr1b10 | | 0.49 | | 22.75 | | 23.41 | 22.89 | 23.24 | 20.95 | 22.64 | 22.4 | 22.8 | 22.46 |  |  |
| Q61239 | Fnta | | 0.87 | | 22.75 | | 22.97 | 22.74 | 22.82 | 21.58 | 23.4 | 22.27 | 22.59 | 22.94 |  |  |
| B7ZCB8 | Stx16 | | 0.65 | | 22.69 | | 22.1 | 22.38 | 21.92 | 21.44 | 22.56 | 21.87 | 24.6 | 21.81 |  |  |
| A0A286YDA2 | Nolc1 | | 0.77 | | 22.69 | | 24.31 | 20.24 | 24.38 | 23.59 | 21.87 | 23.53 | 23.32 | 22.05 |  |  |
| P70698 | Ctps1 | | 0.55 | | 22.69 | | 21.41 | 23.39 | 23.23 | 22.27 | 23.08 | 23.28 | 22.65 | 23.5 |  |  |
| D3YUT6 | S100a1 | | 0.56 | | 22.68 | | 22.54 | 23.81 | 22.23 | 23 | 22.51 | 22.52 | 22.83 | 22.81 |  |  |
| Q8BVQ0 | Wdr61 | | 0.06 | | 22.67 | | 23.13 | 23.48 | 22.23 | 21.31 | 22.78 | 22.19 | 21.83 | 21.89 |  |  |
| P63005 | Pafah1b1 | | 0.47 | | 22.66 | | 21.3 | 23.57 | 17.5 | 22.95 | 23.55 | 23.34 | 23.39 | 23.81 |  |  |
| Q61881 | Mcm7 | | 0.35 | | 22.64 | | 24.07 | 24.28 | 25.13 | 24.42 | 23.87 | 24.28 | 25.1 | 23.98 |  |  |
| P84089 | Erh | | 0.53 | | 22.63 | | 25.3 | 25.15 | 25.2 | 25.6 | 24.84 | 25.65 | 25.05 | 24.5 |  |  |
| A0A1L1SRX2 | Ampd3 | | 0.81 | | 22.62 | | 22.59 | 23.34 | 22.73 | 23.24 | 22.99 | 23.58 | 23.25 | 22.43 |  |  |
| G3X9J6 | Nt5c2 | | 0.49 | | 22.61 | | 21.91 | 24.44 | 23.52 | 24.13 | 24.08 | 23.91 | 23.55 | 22.28 |  |  |
| Q99KC8 | Vwa5a | | 0.78 | | 22.6 | | 24.08 | 23.89 | 23.49 | 23.03 | 23.79 | 24.62 | 24.54 | 22.55 |  |  |
| D3YW19 | 9030624J02Rik | | 0.14 | | 22.59 | | 22.57 | 24.65 | 24.64 | 22.28 | 23.83 | 21.97 | 22 | 17.5 |  |  |
| L7N451 | Gvin1 | | 0.15 | | 22.56 | | 23.16 | 22.12 | 22.91 | 22.76 | 23.42 | 22.18 | 17.5 | 21.54 |  |  |
| Q3UJQ9 | Oxct1 | | 0.3 | | 22.56 | | 23.8 | 22.12 | 23.4 | 22.47 | 17.5 | 22.29 | 25.02 | 24.69 |  |  |
| Q9D071 | Mms19 | | 0.86 | | 22.53 | | 23.74 | 24.01 | 22.93 | 23.49 | 23.62 | 24.01 | 22.95 | 23.85 |  |  |
| F8VQ06 | Ltbp3 | | 0.28 | | 22.52 | | 22.16 | 23.34 | 17.5 | 21.65 | 23.63 | 24.26 | 24.56 | 22.41 |  |  |
| Q8BPI2 | Tmed2 | | 0.92 | | 22.36 | | 23.38 | 19.54 | 21.9 | 19.61 | 25.34 | 24.46 | 22.01 | 21.1 |  |  |
| Q9JJA2 | Cog8 | | 0.34 | | 22.35 | | 21.59 | 17.5 | 22.67 | 22.05 | 21.77 | 22.42 | 22.33 | 22.1 |  |  |
| J3QNW0 | Dnmt1 | | 0.39 | | 22.31 | | 21.89 | 21.6 | 21.73 | 22.29 | 22 | 22.55 | 17.5 | 21.05 |  |  |
| Q9JHZ2 | Ankh | | 0.57 | | 22.3 | | 21.55 | 20.72 | 21.19 | 21.23 | 21.86 | 21.61 | 22.52 | 21.65 |  |  |
| Q8VE47 | Uba5 | | 0.38 | | 22.27 | | 22.39 | 21.47 | 22.29 | 22.39 | 22.41 | 21.94 | 17.5 | 22.22 |  |  |
| O54950 | Prkag1 | | 0.71 | | 22.23 | | 17.5 | 22.9 | 22.22 | 20.8 | 22.15 | 22.07 | 22.16 | 21.95 |  |  |
| E9PX70 | Col12a1 | | 0.4 | | 22.17 | | 23.07 | 22.79 | 23.14 | 22.25 | 21.12 | 24 | 22.62 | 22.67 |  |  |
| Q99J77 | Nans | | 0.28 | | 22.09 | | 23.46 | 21.89 | 25.56 | 22.2 | 22.23 | 25.39 | 25.25 | 23 |  |  |
| A0A0B4J1E7 | Kpna4 | | 0.88 | | 22.04 | | 22.2 | 23.05 | 22.29 | 23.34 | 21.87 | 22.43 | 22.7 | 21.62 |  |  |
| A0A0R4J124 | Srpk2 | | 0.25 | | 22.01 | | 21.34 | 21.9 | 21.94 | 21.59 | 22.67 | 22.35 | 23.43 | 22.05 |  |  |
| S4R1L5 | Birc6 | | 0.7 | | 22.01 | | 25.53 | 23.75 | 26.04 | 22.09 | 17.5 | 23.43 | 22.46 | 23.23 |  |  |
| G3UW30 | Gbe1 | | 0.57 | | 21.99 | | 22.56 | 22.28 | 22.73 | 22.55 | 22.31 | 22 | 22.72 | 22.35 |  |  |
| Q8BWW3 | Pgm3 | | 0.7 | | 21.96 | | 22.64 | 22.82 | 22.91 | 22.58 | 22.8 | 22.57 | 22.04 | 23.09 |  |  |
| Q9CZM3 | Tmem33 | | 0.47 | | 21.82 | | 22.56 | 17.5 | 22.25 | 21.12 | 22.68 | 22.3 | 22.45 | 22 |  |  |
| Q9QWR8 | Naga | | 0.27 | | 21.7 | | 24.49 | 23.25 | 21.73 | 21.12 | 21.69 | 17.5 | 21.2 | 22.85 |  |  |
| Z4YJT3 | Larp1 | | 0.77 | | 21.64 | | 21.83 | 20.68 | 20.97 | 19.59 | 22.01 | 21.99 | 20.58 | 21.06 |  |  |
| Q91ZW3 | Smarca5 | | 0.12 | | 21.51 | | 24.89 | 21.37 | 17.5 | 20.5 | 21.18 | 21.26 | 24.38 | 25.03 |  |  |
| A2AIW9 | Pmpca | | 0.58 | | 21.29 | | 21.19 | 17.5 | 20.85 | 21.37 | 21 | 20.69 | 20.45 | 21.45 |  |  |
| A0A0R4J086 | Olfml3 | | 0.58 | | 21.23 | | 26.12 | 25.09 | 17.5 | 25.61 | 23.41 | 23.65 | 24.27 | 25.95 |  |  |
| Q68FH4 | Galk2 | | 0.62 | | 21.22 | | 21.42 | 21.51 | 22.67 | 21.3 | 20.96 | 20.78 | 21.42 | 21.33 |  |  |
| A2AE27 | Ampd2 | | 0.51 | | 21.16 | | 22.94 | 19.53 | 23.83 | 23.92 | 20.85 | 20.33 | 23.89 | 23.92 |  |  |
| A0A0R4J0H7 | Ncapd2 | | 0.3 | | 21.15 | | 21.04 | 22.52 | 21.04 | 20.89 | 20.81 | 21.01 | 20.97 | 17.5 |  |  |
| P26043 | Rdx | | 0.33 | | 21.12 | | 25.58 | 21.51 | 21.73 | 21.63 | 17.5 | 21.54 | 22.57 | 23.66 |  |  |
| P52633 | Stat6 | | 0.38 | | 17.5 | | 22.86 | 22.67 | 22.92 | 23.4 | 22.66 | 22.9 | 23.22 | 22.36 |  |  |
| H3BKY1 | Glmp | | 0.36 | | 17.5 | | 24.78 | 24.52 | 24.45 | 24.83 | 25.32 | 24.85 | 25.4 | 24.53 |  |  |
| Q8BK64 | Ahsa1 | | 0.42 | | 17.5 | | 25.19 | 25.2 | 25.35 | 24.91 | 25.06 | 25.51 | 25.59 | 24.81 |  |  |
| A0A1Y7VNR7 | Akr1cl | | 0.75 | | 17.5 | | 28.2 | 27.36 | 28.01 | 23.65 | 27.92 | 24.75 | 24.53 | 24.9 |  |  |
| G3X9I4 | Alyref2 | | 0.87 | | 17.5 | | 26.19 | 25.81 | 25.73 | 23.82 | 23.91 | 21.34 | 24.41 | 25.24 |  |  |

Live-BM-EVs: EVs from live *C. neoformans* infected activated BMDMs;

Hk-BM-EVs: EVs from heat-killed *C. neoformans* infected activated BMDMs;

Non-BM-EVs: EVs from activated BMDMs without *C. neoformans* infection;

Hk: heat-killed.
