# Supplementary material for: Cryptococcus neoformans-Infected Macrophages Release Proinflammatory Extracellular Vesicles: Insight into Their Components by Multi-omics
Source: mBio. 2021 Mar 30;12(2):e00279-21. doi: 10.1128/mBio.00279-21 (PMC8092229; doi:10.1128/mBio.00279-21)
Supplement: TABLE S5 [file mBio.00279-21-st005.docx]

Table S5. Core lipids widely present in all EVs from macrophages.

**Table S5. Core lipids present in macrophage EVs from all conditions**

| **Row_ID** | **ANOVA** | **Mode** | **Normalized Log_2_ Intensities** | | | | | | | | |
| --- | --- | --- | --- | --- | --- | --- | --- | --- | --- | --- | --- |
|  |  |  | **live-BM-EVs** | **live-BM-EVs** | **live-BM-EVs** | **hk-BM-EVs** | **hk-BM-EVs** | **hk-BM-EVs** | **Non-BM-EVs** | **Non-BM-EVs** | **Non-BM-EVs** |
| SM(d18:1/16:0)_A | 0.07 | positive ion | 25.43 | 25.34 | 25.39 | 25.33 | 25.17 | 25.39 | 25.41 | 25.52 | 25.55 |
| PC(16:0/18:1) | 0.42 | positive ion | 25.28 | 25.29 | 25.35 | 25.27 | 25.41 | 25.68 | 25.42 | 25.57 | 25.34 |
| PC(16:0/20:1)/PC(18:0/18:1) | 0.34 | positive ion | 25.04 | 25.17 | 25.32 | 25.07 | 25.13 | 25.41 | 25.38 | 25.27 | 25.38 |
| SM(d18:2/24:0) | 0.57 | positive ion | 24.12 | 24.02 | 24.48 | 24.19 | 24.16 | 24.4 | 24.38 | 24.26 | 24.42 |
| PC(16:0/16:0)_A | 0.37 | positive ion | 23.94 | 23.91 | 24.01 | 24.04 | 23.96 | 24.09 | 24.1 | 24.07 | 23.93 |
| PC(16:0/18:0) | 0.12 | positive ion | 23.25 | 23.34 | 23.3 | 23.4 | 23.23 | 23.42 | 23.47 | 23.45 | 23.38 |
| PC(18:1/18:1) | 0.3 | positive ion | 23.21 | 23.27 | 23.28 | 23.3 | 23.38 | 23.59 | 23.36 | 23.51 | 23.22 |
| SM(d18:0/16:0) | 0.97 | positive ion | 23.2 | 23.29 | 23.26 | 23.28 | 23.16 | 23.21 | 22.86 | 23.76 | 23.18 |
| PC(14:0/18:1) | 0.58 | positive ion | 22.92 | 23 | 22.84 | 23.07 | 22.88 | 23.09 | 22.94 | 23.07 | 22.8 |
| PE(18:0/18:1) | 0.26 | negative ion | 22.71 | 22.5 | 23.32 | 23.05 | 23.54 | 24.17 | 22.76 | 22.6 | 23.62 |
| SM(d18:0/18:1)/SM(d18:1/18:0) | 0.43 | positive ion | 22.62 | 22.68 | 22.63 | 22.66 | 22.68 | 22.84 | 22.81 | 22.69 | 22.63 |
| PS(18:0/18:1) | 0.5 | positive ion | 22.52 | 22.54 | 22.57 | 22.54 | 22.63 | 22.45 | 22.58 | 22.59 | 22.6 |
| PC(O-16:0/18:1)/PC(O-18:1/16:0) | 0.12 | positive ion | 22.49 | 22.56 | 22.48 | 22.55 | 22.47 | 22.62 | 22.64 | 22.75 | 22.57 |
| PC(18:0/20:4) | 0.17 | positive ion | 22.46 | 22.31 | 22.59 | 22.46 | 22.54 | 22.74 | 22.31 | 22.48 | 22.18 |
| PC(18:0/22:6) | 0.07 | positive ion | 22.13 | 22.03 | 22.26 | 22.16 | 22.29 | 22.37 | 21.85 | 22.15 | 21.9 |
| PC(O-18:0/14:0) | 0.74 | positive ion | 22.04 | 22.13 | 22.03 | 22.1 | 22.06 | 21.9 | 22.02 | 22.12 | 22.01 |
| PC(16:0/18:2) | 0.68 | positive ion | 22.02 | 22.12 | 21.7 | 22.06 | 21.94 | 21.9 | 21.4 | 22.24 | 21.7 |
| SM(d18:1/23:0) | 0.34 | positive ion | 22.01 | 22.15 | 22.16 | 22.19 | 22.21 | 22.27 | 22.09 | 22.29 | 22.02 |
| PC(O-18:0/16:0) | 0.24 | positive ion | 22 | 22.14 | 22.17 | 22.13 | 21.95 | 21.71 | 22.23 | 22 | 22.25 |
| PC(14:0/16:0) | 0.78 | positive ion | 21.98 | 22.01 | 21.7 | 21.95 | 21.71 | 21.82 | 21.89 | 21.86 | 21.74 |
| SM(d18:1/23:1) | 0.13 | positive ion | 21.94 | 21.93 | 22 | 21.94 | 21.94 | 21.98 | 21.98 | 22.08 | 22.02 |
| PC(18:0/22:5) | 0.45 | positive ion | 21.92 | 21.77 | 22.11 | 21.8 | 22.08 | 22.27 | 21.69 | 22.01 | 21.81 |
| PS(18:0/18:1) | 0.18 | negative ion | 21.83 | 21.9 | 22.1 | 22.1 | 22.94 | 22.84 | 21.36 | 21.96 | 22.56 |
| PE(16:0/18:1)/PE(16:1/18:0) | 0.27 | positive ion | 21.75 | 21.83 | 21.6 | 21.84 | 21.66 | 21.67 | 21.96 | 21.88 | 21.75 |
| SM(d18:2/16:0)_A | 0.58 | positive ion | 21.75 | 21.72 | 21.56 | 21.76 | 21.64 | 22.1 | 21.58 | 21.91 | 21.81 |
| GalCer(d18:1/24:0) | 0.18 | positive ion | 21.69 | 21.58 | 21.44 | 21.63 | 21.66 | 21.49 | 21.82 | 21.67 | 21.7 |
| PC(18:0/20:3) | 0.3 | positive ion | 21.69 | 21.51 | 21.8 | 21.54 | 21.67 | 21.79 | 21.41 | 21.66 | 21.41 |
| PC(16:0/19:1) | 0.97 | positive ion | 21.64 | 21.67 | 21.65 | 21.62 | 21.6 | 21.71 | 21.61 | 21.74 | 21.61 |
| PC(16:0/20:2)/PC(18:0/18:2) | 0.54 | positive ion | 21.59 | 21.64 | 21.34 | 21.5 | 21.53 | 21.58 | 21.49 | 21.56 | 21.17 |
| PE(18:1/18:1) | 0.15 | negative ion | 21.5 | 21.48 | 21.9 | 21.95 | 22.06 | 22.9 | 21.82 | 21.62 | 22.16 |
| PE(18:0/20:4) | 1 | positive ion | 21.42 | 21.54 | 21.07 | 21.51 | 21.36 | 21.17 | 21.43 | 21.47 | 21.17 |
| PC(16:0/20:4) | 0.33 | positive ion | 21.39 | 21.23 | 21.12 | 21.36 | 21.35 | 21.52 | 21.03 | 21.47 | 21.05 |
| PC(18:1/18:2) | 0.22 | positive ion | 21.37 | 21.42 | 21.18 | 21.32 | 21.32 | 21.38 | 21.03 | 21.38 | 20.93 |
| PC(16:0/16:1) | 0.77 | positive ion | 21.34 | 21.37 | 21.44 | 21.33 | 21.56 | 21.62 | 21.23 | 21.81 | 21.08 |
| PC(15:0/18:1) | 0.25 | positive ion | 21.32 | 21.52 | 21.35 | 21.48 | 21.45 | 21.5 | 21.28 | 21.48 | 21.18 |
| PC(16:0/17:1) | 0.25 | positive ion | 21.32 | 21.52 | 21.35 | 21.48 | 21.45 | 21.5 | 21.28 | 21.48 | 21.18 |
| SM(d18:1/17:0) | 0.23 | positive ion | 21.3 | 21.36 | 21.25 | 21.39 | 21.36 | 21.34 | 21.03 | 21.34 | 21.25 |
| SM(d16:1/17:0)/SM(d18:1/15:0) | 0.59 | positive ion | 21.28 | 21.11 | 21.03 | 21.34 | 21 | 21.1 | 20.61 | 21.34 | 20.92 |
| SM(d16:1/22:0)/SM(d18:1/20:0) | 0.81 | positive ion | 21.21 | 21.32 | 21.31 | 21.25 | 21.13 | 21.35 | 21.29 | 21.19 | 21.4 |
| PC(O-18:0/18:1) | 0.22 | positive ion | 21.19 | 21.25 | 21.05 | 21.09 | 20.99 | 21.01 | 21.13 | 21.44 | 21.11 |
| PC(16:1/18:1) | 0.49 | positive ion | 21.18 | 21.13 | 21.02 | 21.23 | 21.19 | 21.49 | 21.11 | 21.46 | 20.93 |
| SM(d16:1/24:1)/SM(d18:1/22:1)/ | 0.36 | positive ion | 21.17 | 21.15 | 21.32 | 21.2 | 21.07 | 21.17 | 21.22 | 21.2 | 21.25 |
| SM(d18:2/22:0) |  |  |  |  |  |  |  |  |  |  |  |
| PC(15:0/16:0)_A | 0.17 | positive ion | 20.93 | 20.99 | 20.76 | 20.95 | 20.67 | 20.73 | 20.46 | 20.83 | 20.57 |
| PC(0:0/18:0) | 0.84 | positive ion | 20.79 | 20.61 | 21.4 | 21.08 | 20.77 | 21.05 | 20.46 | 20.63 | 21.3 |
| SM(d18:1/26:1) | 0.76 | positive ion | 20.7 | 20.88 | 21.03 | 20.99 | 20.78 | 20.94 | 20.89 | 20.96 | 20.97 |
| PE(16:0/18:1) | 0.05 | negative ion | 20.69 | 20.77 | 21 | 21.08 | 21.38 | 21.85 | 20.97 | 20.77 | 21.02 |
| PC(15:1/18:1) | 0.74 | positive ion | 20.68 | 20.83 | 20.39 | 20.71 | 20.68 | 20.59 | 20.83 | 20.91 | 20.5 |
| PE(18:0/18:2) | 0.74 | positive ion | 20.68 | 20.83 | 20.39 | 20.71 | 20.68 | 20.59 | 20.83 | 20.91 | 20.5 |
| PC(16:0/20:3)/PC(16:0/20:3) | 0.09 | positive ion | 20.65 | 20.81 | 20.64 | 20.67 | 20.7 | 20.74 | 20.48 | 20.67 | 20.39 |
| SM(d18:0/22:0) | 0.98 | positive ion | 20.62 | 20.68 | 20.47 | 20.62 | 20.62 | 20.52 | 20.66 | 20.74 | 20.41 |
| SM(d16:1/20:1)/SM(d18:2/18:0) | 0.51 | positive ion | 20.61 | 20.64 | 20.76 | 20.73 | 20.68 | 21.02 | 20.5 | 20.82 | 20.76 |
| PC(0:0/16:0) | 0.93 | positive ion | 20.5 | 19.81 | 21.44 | 20.13 | 20.59 | 20.29 | 19.86 | 19.91 | 21.81 |
| SM(d18:0/24:0) | 0.93 | positive ion | 20.48 | 20.62 | 20.41 | 20.54 | 20.41 | 20.5 | 20.42 | 20.6 | 20.52 |
| PE(20:0/20:5) | 0.98 | positive ion | 20.45 | 20.47 | 19.74 | 20.43 | 20.33 | 19.84 | 20.49 | 20.43 | 19.85 |
| GalCer(d18:1/24:1) | 0.46 | positive ion | 20.44 | 20.58 | 20.49 | 20.45 | 20.53 | 20.65 | 20.81 | 20.45 | 20.65 |
| SM(d17:1/26:1)/SM(d18:2/25:0)/ | 0.1 | positive ion | 20.36 | 20.54 | 20.48 | 20.66 | 20.55 | 20.54 | 20.57 | 20.58 | 20.61 |
| SM(d19:1/24:1) |  |  |  |  |  |  |  |  |  |  |  |
| SM(d16:1/16:0) | 0.39 | positive ion | 20.36 | 20.32 | 20.13 | 20.47 | 20.19 | 20.38 | 20.08 | 20.28 | 20.25 |
| GalCer(d18:1/22:0) | 0.62 | positive ion | 20.26 | 20.5 | 19.95 | 20.23 | 20.19 | 19.63 | 20.52 | 20.26 | 19.92 |
| GalCer(d18:1/16:0) | 0.3 | negative ion | 20.23 | 20.15 | 20.23 | 20.19 | 20.85 | 21.03 | 20.39 | 20.05 | 20.86 |
| GalCer(d18:1/24:0) | 0.07 | negative ion | 20.17 | 20.15 | 20.41 | 20.37 | 20.82 | 20.95 | 20.21 | 19.97 | 20.43 |
| PC(18:1/20:1) | 0.39 | positive ion | 20.1 | 20.31 | 20.15 | 20.13 | 20.17 | 20.36 | 20.51 | 20.27 | 20.23 |
| PE(18:1/20:3) | 0.19 | positive ion | 20.07 | 20.29 | 20.13 | 20.08 | 20.2 | 20.11 | 21.25 | 20.11 | 20.59 |
| SM(d18:2/23:0) | 0.56 | positive ion | 20.05 | 20.11 | 20.28 | 20.18 | 20.17 | 20.21 | 20.07 | 20.12 | 20.17 |
| PC(18:0/20:1) | 0.38 | positive ion | 19.95 | 19.97 | 19.77 | 19.81 | 19.76 | 19.84 | 20.1 | 20.06 | 19.74 |
| PC(18:0/18:0) | 0.84 | positive ion | 19.89 | 19.91 | 19.72 | 19.82 | 19.87 | 19.74 | 19.91 | 19.9 | 19.75 |
| PE(18:0/22:6) | 0.96 | positive ion | 19.87 | 20.08 | 19.25 | 20.02 | 19.92 | 19.51 | 19.99 | 19.99 | 19.36 |
| PC(0:0/18:1) | 0.87 | positive ion | 19.84 | 18.84 | 21.38 | 19.08 | 20.2 | 18.94 | 18.36 | 18.69 | 21.95 |
| PS(18:0/22:6) | 0.78 | positive ion | 19.77 | 19.78 | 19.36 | 19.79 | 19.63 | 19.32 | 19.53 | 19.56 | 19.46 |
| GalCer(d18:1/16:0) | 0.5 | positive ion | 19.76 | 19.94 | 19.59 | 19.78 | 19.71 | 19.74 | 19.57 | 19.76 | 19.63 |
| PC(18:1/22:5) | 0.23 | positive ion | 19.76 | 19.63 | 19.7 | 19.74 | 19.63 | 19.65 | 19.06 | 19.72 | 19.45 |
| LacCer(d18:1/24:1) | 0.89 | positive ion | 19.67 | 19.98 | 19.97 | 19.79 | 19.83 | 19.94 | 19.96 | 19.94 | 19.8 |
| PC(O-16:0/16:1) | 0.65 | positive ion | 19.64 | 19.76 | 19.31 | 19.57 | 19.47 | 19.33 | 19.58 | 20.01 | 19.36 |
| SM(d18:1/25:0)/SM(d20:1/23:0) | 0.22 | positive ion | 19.64 | 19.59 | 19.58 | 19.64 | 19.4 | 19.33 | 19.59 | 19.56 | 19.64 |
| SM(d16:0/20:0)/SM(d18:0/18:0)/ | 0.83 | positive ion | 19.48 | 19.56 | 19.31 | 19.57 | 19.43 | 19.35 | 19.24 | 19.63 | 19.27 |
| SM(d19:0/17:0) |  |  |  |  |  |  |  |  |  |  |  |
| SM(d18:1/26:0) | 0.98 | positive ion | 19.48 | 19.56 | 19.64 | 19.72 | 19.52 | 19.48 | 19.58 | 19.56 | 19.56 |
| PE(17:0/18:1) | 0.8 | positive ion | 19.4 | 19.64 | 19.32 | 19.6 | 19.32 | 19.3 | 19.69 | 19.5 | 19.32 |
| PE(18:0/0:0) | 0.44 | positive ion | 19.38 | 19.25 | 19.24 | 18.96 | 19.16 | 19.08 | 18.77 | 18.85 | 19.51 |
| Cer(d18:1/24:1) | 0.7 | positive ion | 19.37 | 19.28 | 18.68 | 19.52 | 19.4 | 18.93 | 19.96 | 19.34 | 18.91 |
| GalCer(d18:1/24:1) | 0.41 | negative ion | 19.29 | 19.55 | 19.76 | 19.42 | 19.97 | 20.24 | 19.26 | 19.41 | 19.92 |
| PC(18:1/22:6) | 0.08 | positive ion | 19.27 | 19.26 | 19.13 | 19.21 | 19.08 | 19.31 | 18.95 | 19.14 | 18.87 |
| Cer(d18:1/24:0) | 0.81 | positive ion | 19.26 | 19.29 | 18.55 | 19.37 | 19.31 | 18.93 | 19.72 | 19.34 | 18.67 |
| PC(16:0/17:0) | 0.81 | positive ion | 19.21 | 19.19 | 18.97 | 19.17 | 19.09 | 19.07 | 19.12 | 19.17 | 18.89 |
| PC(18:0/24:1)/PC(18:1/24:0) | 0.12 | positive ion | 19.17 | 19.26 | 19.2 | 19.37 | 19.3 | 19.4 | 19.46 | 19.4 | 19.21 |
| PC(17:1/18:1) | 0.41 | positive ion | 19.13 | 19.22 | 19.03 | 19.14 | 19.12 | 19.2 | 18.98 | 19.22 | 18.81 |
| PS(18:0/18:2)/PS(18:1/18:1) | 0.86 | positive ion | 19.09 | 19.21 | 18.87 | 19.12 | 19.06 | 18.77 | 19 | 19.18 | 18.93 |
| PS(16:0/18:1)/PS(16:1/18:0) | 0.7 | positive ion | 19.06 | 19.11 | 18.78 | 19.07 | 18.84 | 18.78 | 19.02 | 19 | 18.92 |
| PC(O-20:0/16:0) | 0.67 | positive ion | 19.05 | 19.26 | 19.06 | 19.15 | 19.22 | 19.26 | 19.05 | 19.33 | 19.15 |
| PE(18:0/20:2)/PE(18:1/20:1) | 0.08 | positive ion | 18.98 | 19.17 | 18.99 | 19.14 | 19.05 | 19.06 | 19.45 | 19.22 | 19.17 |
| GM2(42:1) | 0.88 | negative ion | 18.96 | 19.03 | 18.71 | 19.31 | 19.42 | 17.89 | 19.23 | 19.09 | 18.91 |
| PE(18:1/20:4) | 0.94 | positive ion | 18.93 | 19.09 | 18.43 | 18.97 | 18.92 | 18.78 | 19.05 | 19.01 | 18.56 |
| PE(18:1/0:0) | 0.58 | positive ion | 18.92 | 18.51 | 19.64 | 18.2 | 19.06 | 18.81 | 18.68 | 18.84 | 20.34 |
| Cer(d18:1/16:0) | 0.96 | positive ion | 18.82 | 18.94 | 17.72 | 18.77 | 18.63 | 17.93 | 18.98 | 18.82 | 17.92 |
| PE(18:0/20:1) | 0.19 | positive ion | 18.81 | 18.98 | 18.78 | 18.84 | 18.86 | 18.77 | 19.38 | 18.98 | 18.89 |
| Cer(d18:1/16:0) | 0.68 | negative ion | 18.79 | 18.95 | 17.71 | 19.05 | 18.94 | 18.5 | 18.88 | 18.76 | 18.09 |
| GM2(42:2) | 0.52 | negative ion | 18.79 | 18.82 | 18.74 | 19.24 | 19.43 | 18.2 | 19.17 | 19.07 | 19.25 |
| PC(18:0/19:1) | 0.86 | positive ion | 18.79 | 18.76 | 18.75 | 18.65 | 18.73 | 18.89 | 18.73 | 18.79 | 18.67 |
| Cer(d18:1/24:1) | 0.57 | negative ion | 18.78 | 19.06 | 18.18 | 18.98 | 19.27 | 18.83 | 19.66 | 18.76 | 18.6 |
| PC(18:1/20:3) | 0.47 | positive ion | 18.76 | 18.74 | 18.49 | 18.68 | 18.78 | 18.71 | 18.54 | 18.82 | 18.19 |
| PE(17:1/18:0) | 0.17 | negative ion | 18.71 | 18.81 | 18.73 | 19.06 | 18.64 | 19.32 | 18.64 | 18.75 | 18.53 |
| Cer(d18:1/24:0) | 0.46 | negative ion | 18.69 | 18.99 | 18.01 | 18.95 | 19.17 | 18.95 | 19.24 | 18.84 | 18.22 |
| PC(17:0/18:1) | 0.78 | positive ion | 18.69 | 18.8 | 18.64 | 18.68 | 18.66 | 18.79 | 18.56 | 18.93 | 18.38 |
| PE(18:1/18:3) | 0.26 | positive ion | 18.67 | 18.88 | 18.64 | 18.77 | 18.64 | 18.66 | 19.39 | 18.66 | 18.96 |
| PE(16:0/16:1) | 0.23 | negative ion | 18.63 | 18.99 | 18.11 | 18.92 | 18.72 | 19.42 | 18.01 | 18.92 | 18.04 |
| PC(16:0/22:4) | 0.08 | positive ion | 18.58 | 18.7 | 18.51 | 18.63 | 18.63 | 18.66 | 18.23 | 18.61 | 18.21 |
| PC(O-16:0/22:5) | 0.28 | positive ion | 18.58 | 18.62 | 18.34 | 18.61 | 18.68 | 18.39 | 18.31 | 18.54 | 18.16 |
| SM(d18:1/16:0)_C | 0.19 | positive ion | 18.58 | 18.67 | 18.67 | 18.8 | 18.59 | 18.69 | 18.34 | 18.57 | 18.62 |
| PE(18:1/18:2) | 0.73 | positive ion | 18.52 | 18.76 | 18.46 | 18.59 | 18.51 | 18.97 | 18.85 | 18.7 | 18.53 |
| SM(d18:0/20:0) | 0.34 | positive ion | 18.51 | 18.49 | 18.62 | 18.55 | 18.53 | 18.74 | 18.36 | 18.58 | 18.48 |
| PC(18:1/19:1) | 0.61 | positive ion | 18.5 | 18.59 | 18.32 | 18.4 | 18.37 | 18.32 | 18.5 | 18.6 | 18.25 |
| PC(16:0/24:1) | 0.75 | positive ion | 18.49 | 18.59 | 18.45 | 18.56 | 18.42 | 18.39 | 18.65 | 18.42 | 18.45 |
| PE(18:0/20:1)/PE(18:1/20:0) | 0.68 | negative ion | 18.49 | 18.46 | 18.55 | 18.45 | 18.48 | 19.05 | 18.74 | 18.45 | 18.38 |
| SM(d18:2/18:1) | 0.59 | positive ion | 18.48 | 18.62 | 18.66 | 18.81 | 18.94 | 19.02 | 19.74 | 18.27 | 18.75 |
| PE(16:1/18:1) | 0.42 | positive ion | 18.46 | 18.69 | 18.47 | 18.47 | 18.44 | 18.7 | 18.89 | 18.64 | 18.53 |
| PC(14:0/16:1)/PC(14:1/16:0) | 0.45 | positive ion | 18.44 | 18.58 | 18.38 | 18.44 | 18.4 | 18.61 | 18.46 | 18.45 | 18.09 |
| PC(O-16:1/20:4) | 0.09 | positive ion | 18.42 | 18.59 | 18.58 | 18.48 | 18.52 | 18.66 | 18.22 | 18.49 | 18.2 |
| Cer(d18:1/22:0) | 0.93 | positive ion | 18.4 | 18.59 | 17.41 | 18.45 | 18.32 | 17.61 | 18.79 | 18.34 | 17.71 |
| GalCer(d18:0/24:0) | 0.26 | positive ion | 18.4 | 18.45 | 18.32 | 18.39 | 18.3 | 18.21 | 18.44 | 18.44 | 18.32 |
| TG(16:0/16:0/18:1) | 0.47 | positive ion | 18.4 | 18.29 | 18.48 | 18.76 | 18.43 | 19.02 | 19.51 | 18.67 | 18.23 |
| TG(12:0/18:0/18:0)/TG(14:0/14:0/20:0)/ | 0.33 | positive ion | 18.38 | 18.57 | 18.47 | 18.37 | 18.37 | 18.56 | 19.71 | 18.32 | 18.77 |
| TG(14:0/16:0/18:0)/TG(15:0/16:0/17:0)/ |  |  |  |  |  |  |  |  |  |  |  |
| TG(16:0/16:0/16:0) |  |  |  |  |  |  |  |  |  |  |  |
| PS(18:0/19:1) | 0.33 | positive ion | 18.38 | 18.32 | 18.45 | 18.6 | 18.45 | 18.28 | 18.23 | 18.42 | 18.18 |
| PS(18:0/20:4) | 0.82 | positive ion | 18.32 | 18.35 | 17.84 | 18.28 | 18.19 | 17.78 | 18 | 18.25 | 17.88 |
| PC(14:0/14:0) | 0.44 | positive ion | 18.3 | 18.44 | 18.13 | 18.53 | 18.19 | 18.27 | 18.23 | 18.28 | 17.92 |
| PE(16:0/20:4) | 0.99 | positive ion | 18.3 | 18.41 | 17.71 | 18.26 | 18.19 | 17.97 | 18.37 | 18.24 | 17.73 |
| TG(16:0/18:1/18:1) | 0.28 | positive ion | 18.26 | 18.21 | 18.34 | 18.54 | 18.36 | 18.84 | 19.44 | 18.62 | 18.33 |
| PE(18:0/22:4) | 0.97 | positive ion | 18.24 | 18.49 | 17.18 | 18.05 | 17.97 | 17.66 | 18.18 | 18.18 | 17.55 |
| PC(17:0/22:5) | 0.14 | positive ion | 18.17 | 18.05 | 18.14 | 18.03 | 18.12 | 18.09 | 17.91 | 18.09 | 17.73 |
| GM3(42:1) | 0.99 | negative ion | 18.16 | 18.4 | 18.25 | 18.59 | 18.6 | 17.69 | 18.44 | 18.53 | 17.84 |
| PC(O-16:0/17:1) | 0.87 | positive ion | 18.1 | 18.26 | 17.98 | 18.01 | 18.1 | 18.05 | 18.09 | 18.27 | 17.76 |
| PC(15:0/16:1) | 0.21 | positive ion | 18.09 | 18.06 | 17.72 | 18 | 17.79 | 17.9 | 17.6 | 17.91 | 17.54 |
| PE(18:0/20:5) | 0.75 | positive ion | 18.09 | 18.21 | 17.69 | 18 | 17.97 | 17.77 | 17.88 | 18.05 | 17.67 |
| PG(18:0/18:1) | 0.08 | negative ion | 18.04 | 18.07 | 17.94 | 18.15 | 18.23 | 18.11 | 17.73 | 18.09 | 17.6 |
| PC(17:0/20:4) | 0.21 | positive ion | 18.03 | 18.04 | 18 | 17.95 | 18.06 | 18.16 | 17.83 | 18.05 | 17.48 |
| PE(18:1/0:0) | 0.78 | negative ion | 18.01 | 17.63 | 18.92 | 17.92 | 18.95 | 18.78 | 17.87 | 18.24 | 19.56 |
| PE(16:0/18:0) | 0.29 | negative ion | 17.99 | 17.87 | 17.98 | 18.2 | 18.05 | 18.59 | 18.33 | 17.89 | 17.53 |
| PE(18:0/0:0) | 0.52 | negative ion | 17.93 | 18.02 | 17.86 | 18.1 | 17.92 | 18.34 | 16.94 | 17.81 | 18.34 |
| SM(d18:0/17:0) | 0.64 | positive ion | 17.92 | 18.11 | 17.85 | 18.19 | 17.98 | 17.91 | 17.95 | 18.06 | 17.68 |
| PC(14:0/15:0) | 0.51 | positive ion | 17.9 | 17.77 | 17.29 | 17.78 | 17.41 | 17.6 | 17.2 | 17.7 | 17.28 |
| PC(16:0/16:0)_B | 0.51 | positive ion | 17.88 | 17.97 | 17.44 | 17.95 | 17.69 | 17.54 | 17.59 | 17.78 | 17.14 |
| PS(16:1/18:0)/PS(16:0/18:1) | 0.49 | negative ion | 17.88 | 18.18 | 17.73 | 18.12 | 17.7 | 18.06 | 17.85 | 17.87 | 17.58 |
| GalCer(d18:1/23:0) | 0.63 | positive ion | 17.84 | 18.19 | 17.82 | 17.95 | 17.9 | 17.62 | 17.86 | 17.92 | 17.8 |
| PC(15:0/16:0)_B | 0.27 | positive ion | 17.84 | 17.87 | 17.36 | 17.75 | 17.6 | 17.67 | 17.37 | 17.65 | 17.21 |
| PS(18:0/20:3) | 0.79 | positive ion | 17.84 | 18.06 | 17.63 | 17.94 | 17.77 | 17.49 | 17.99 | 17.78 | 17.67 |
| PC(24:0/0:0) | 0.28 | positive ion | 17.82 | 17.91 | 17.72 | 18.09 | 17.67 | 17.91 | 17.62 | 17.82 | 17.57 |
| PE(18:1/20:1) | 0.77 | negative ion | 17.81 | 17.95 | 17.96 | 18.17 | 17.49 | 18.59 | 18.05 | 17.88 | 17.81 |
| TG(16:0/16:0/18:0) | 0.65 | positive ion | 17.76 | 18.05 | 17.8 | 17.9 | 17.89 | 17.98 | 19.15 | 17.76 | 17.74 |
| PI(16:0/18:1) | 0.16 | negative ion | 17.73 | 18.04 | 17.53 | 18.47 | 17.79 | 18.08 | 17.48 | 17.89 | 17.41 |
| PE(16:1/18:1) | 0.65 | negative ion | 17.72 | 18.21 | 17.68 | 17.98 | 17.8 | 18.46 | 18.06 | 18 | 17.81 |
| PS(18:1/18:1) | 0.36 | negative ion | 17.71 | 17.89 | 17.39 | 17.95 | 17.45 | 17.57 | 17.49 | 17.54 | 17.16 |
| Cer(d18:1/22:0) | 0.93 | negative ion | 17.68 | 17.98 | 16.62 | 17.87 | 17.71 | 17.08 | 18.13 | 17.72 | 16.96 |
| PS(18:0/22:4) | 0.77 | positive ion | 17.64 | 17.93 | 17.14 | 17.68 | 17.49 | 17.05 | 17.38 | 17.58 | 17.27 |
| GalCer(d18:0/16:0) | 0.39 | positive ion | 17.6 | 17.62 | 17.51 | 17.76 | 17.75 | 17.63 | 17.8 | 17.77 | 17.47 |
| PC(16:0/24:0) | 0.17 | positive ion | 17.59 | 17.82 | 17.69 | 17.87 | 17.96 | 17.87 | 17.93 | 17.99 | 17.68 |
| TG(16:0/18:0/18:1) | 0.48 | positive ion | 17.43 | 17.68 | 17.49 | 17.88 | 17.62 | 18.18 | 18.56 | 18 | 17.26 |
| GM3(42:2) | 0.73 | negative ion | 17.42 | 17.87 | 17.37 | 18.19 | 18.05 | 17.12 | 17.58 | 18.03 | 17.72 |
| PE(17:1/18:1) | 0.96 | positive ion | 17.39 | 17.72 | 17.37 | 17.54 | 17.43 | 17.48 | 17.75 | 17.6 | 17.24 |
| TG(14:0/20:1/20:1)/TG(16:0/18:1/20:1)/ | 0.29 | positive ion | 17.38 | 17.27 | 17.47 | 17.36 | 17.1 | 17.72 | 18.19 | 17.76 | 17.34 |
| TG(16:1/18:0/20:1)/TG(18:0/18:1/18:1) |  |  |  |  |  |  |  |  |  |  |  |
| PG(16:0/18:1) | 0.09 | negative ion | 17.3 | 17.27 | 16.95 | 17.77 | 17.26 | 17.29 | 16.69 | 17.2 | 16.54 |
| PC(16:0/20:5)/PC(16:0/20:5) | 0.42 | positive ion | 17.25 | 17.26 | 16.63 | 17.19 | 17.08 | 17.04 | 16.91 | 17.09 | 16.21 |
| TG(14:0/18:1/18:1)/TG(14:1/18:0/18:1)/ | 0.41 | positive ion | 17.24 | 17.46 | 17.62 | 17.83 | 17.55 | 18.16 | 18.37 | 17.9 | 17.23 |
| TG(16:0/16:0/18:2)/TG(16:0/16:1/18:1)/ |  |  |  |  |  |  |  |  |  |  |  |
| TG(16:1/16:1/18:0) |  |  |  |  |  |  |  |  |  |  |  |
| TG(14:0/14:0/20:1)/TG(14:0/16:0/18:1)/ | 0.28 | positive ion | 17.19 | 17.12 | 17.35 | 17.59 | 17.34 | 17.76 | 18.09 | 17.67 | 17.15 |
| TG(14:0/16:1/18:0)/TG(12:0/16:0/20:0) |  |  |  |  |  |  |  |  |  |  |  |
| TG(14:0/16:0/16:0) | 0.4 | positive ion | 17.11 | 17.37 | 17.12 | 17.24 | 17.36 | 17.35 | 18.21 | 17.14 | 17.41 |
| TG(16:0/18:1/20:2)/TG(16:1/18:1/20:1)/ | 0.08 | positive ion | 16.93 | 16.99 | 16.93 | 17.23 | 16.96 | 17.4 | 18.62 | 17.1 | 18.99 |
| TG(18:0/18:1/18:2)/TG(18:1/18:1/18:1) |  |  |  |  |  |  |  |  |  |  |  |
| PC(16:1/16:1) | 0.58 | positive ion | 16.89 | 17.16 | 16.84 | 17.12 | 17 | 17.27 | 17.2 | 17.06 | 16.62 |
| PI(16:0/18:0) | 0.73 | negative ion | 16.75 | 17.21 | 16.52 | 17.28 | 16.72 | 16.72 | 16.85 | 16.94 | 16.24 |
| GalCer(d18:0/16:0) | 0.9 | negative ion | 16.69 | 16.94 | 16.67 | 16.88 | 16.44 | 16.85 | 16.64 | 17.03 | 16.34 |
| Cer(d18:0/24:0) | 0.79 | positive ion | 16.68 | 16.69 | 16.16 | 16.47 | 16.67 | 16.32 | 16.97 | 16.74 | 16.23 |
| PC(16:0/26:0) | 0.66 | positive ion | 16.67 | 16.81 | 16.59 | 16.74 | 16.81 | 16.75 | 16.82 | 16.73 | 16.53 |
| PC(O-16:0/0:0) | 0.78 | positive ion | 16.67 | 16.37 | 16.88 | 16.6 | 16.43 | 16.4 | 15.78 | 15.89 | 17.33 |
| TG(16:0/16:0/20:3)/TG(16:0/18:1/18:2)/ | 0.31 | positive ion | 16.63 | 16.67 | 17.06 | 17.16 | 16.87 | 17.59 | 17.77 | 17.2 | 16.81 |
| TG(16:1/18:0/18:2)/TG(16:1/18:1/18:1) |  |  |  |  |  |  |  |  |  |  |  |
| SM(d18:0/14:0) | 0.35 | positive ion | 16.57 | 16.72 | 16.48 | 16.66 | 16.35 | 16.44 | 16.5 | 16.46 | 16.32 |
| GM3(40:1) | 0.83 | negative ion | 16.53 | 16.81 | 16.2 | 16.79 | 16.74 | 15.64 | 16.68 | 17.15 | 16.12 |
| PC(16:1/0:0) | 0.85 | positive ion | 16.5 | 15.27 | 18.18 | 15.24 | 16.9 | 15.5 | 14.61 | 14.93 | 18.84 |
| SM(d18:0/15:0) | 0.12 | positive ion | 16.43 | 16.52 | 16.35 | 16.71 | 16.45 | 16.48 | 16.16 | 16.46 | 16.17 |
| PA(18:0/18:1) | 0.67 | negative ion | 16.42 | 16.6 | 16.27 | 16.56 | 16.24 | 16.24 | 16.62 | 16.31 | 15.67 |
| PC(22:1/22:1) | 0.44 | positive ion | 16.41 | 16.48 | 16.53 | 16.73 | 16.51 | 16.83 | 16.86 | 16.78 | 16.28 |
| GM2(40:1) | 0.51 | negative ion | 16.4 | 16.52 | 15.69 | 16.7 | 16.32 | 14.92 | 16.88 | 16.93 | 16.07 |
| PC(17:0/0:0) | 0.81 | positive ion | 16.4 | 15.92 | 17.21 | 16.32 | 16.47 | 16.02 | 15.76 | 15.83 | 17.07 |
| PC(O-18:0/0:0) | 0.22 | positive ion | 16.37 | 16.33 | 16.39 | 16.33 | 15.78 | 16.14 | 15.54 | 15.89 | 16.32 |
| TG(16:0/18:0/18:0) | 0.55 | positive ion | 16.34 | 16.88 | 16.39 | 16.54 | 16.44 | 16.38 | 17.51 | 16.47 | 16.45 |
| PE(17:1/18:1) | 0.92 | negative ion | 16.23 | 16.63 | 16.37 | 16.9 | 16.14 | 16.64 | 16.85 | 16.87 | 15.75 |
| PE(16:0/0:0) | 0.71 | positive ion | 16.07 | 16.11 | 16.59 | 16.02 | 15.97 | 16.16 | 16.09 | 15.6 | 16.55 |
| PS(17:1/18:0) | 0.77 | negative ion | 15.99 | 16.49 | 15.88 | 16.41 | 15.65 | 15.7 | 16.03 | 16.26 | 15.49 |
| SM(d18:1/16:0)_B | 0.85 | positive ion | 15.98 | 16.02 | 15.64 | 16.15 | 15.75 | 15.9 | 15.62 | 16.13 | 15.71 |
| PC(0:0/20:4) | 0.88 | positive ion | 15.93 | 14.21 | 17.81 | 14.13 | 16.54 | 15.05 | 14.39 | 14.03 | 18.03 |
| TG(15:0/16:0/18:1) | 0.53 | positive ion | 15.88 | 15.79 | 15.89 | 15.95 | 16.01 | 16.46 | 17.01 | 16.24 | 15.58 |
| PC(20:0/0:0) | 0.68 | positive ion | 15.8 | 15.43 | 15.01 | 15.38 | 15.5 | 15.54 | 15.29 | 15.37 | 15.25 |
| PC(22:6/0:0) | 0.91 | positive ion | 15.8 | 14.25 | 17.67 | 14.35 | 16.49 | 15.17 | 14.63 | 14.17 | 17.87 |
| PG(16:0/18:0) | 0.46 | negative ion | 15.7 | 15.85 | 15.24 | 16.16 | 15.28 | 15.32 | 15.26 | 15.61 | 14.67 |
| PC(0:0/14:0) | 0.74 | positive ion | 15.69 | 14.74 | 16.92 | 14.26 | 15.76 | 14.72 | 13.99 | 14.47 | 17.47 |
| PC(22:0/0:0) | 0.82 | positive ion | 15.61 | 16.04 | 15.41 | 15.81 | 15.71 | 15.83 | 15.68 | 15.79 | 15.66 |
| PG(16:1/18:1) | 0.3 | negative ion | 15.46 | 15.66 | 15.03 | 15.92 | 15.19 | 15.46 | 14.7 | 15.63 | 14.25 |
| SM(d18:2/14:0) | 0.55 | positive ion | 15.38 | 15.58 | 15.48 | 15.25 | 15.22 | 15.61 | 15.51 | 15.48 | 15.41 |
| PC(20:1/0:0) | 0.96 | positive ion | 15.28 | 14.88 | 16.3 | 15.37 | 15.6 | 15.5 | 14.31 | 14.88 | 16.76 |
| GM3(34:1) | 0.96 | negative ion | 15.2 | 15.55 | 14.88 | 15.44 | 15.31 | 15.32 | 15.88 | 16.02 | 14 |
| PC(O-18:1/0:0) | 0.92 | positive ion | 15.15 | 14.72 | 15.88 | 15.15 | 15.2 | 14.67 | 14.28 | 14.81 | 16.42 |
| PE(22:6/0:0) | 0.52 | positive ion | 15.14 | 14.38 | 14.81 | 14.25 | 15.05 | 14.12 | 15.04 | 14.57 | 14.88 |
| TG(16:0/16:1/18:2) | 0.34 | positive ion | 15.07 | 14.97 | 15.34 | 15.58 | 15.32 | 15.89 | 16.07 | 15.76 | 14.89 |
| PC(18:2/18:2) | 0.29 | positive ion | 15.05 | 15.09 | 14.45 | 14.98 | 14.96 | 17.38 | 14.66 | 14.98 | 14.13 |
| PC(20:3/0:0) | 0.91 | positive ion | 14.99 | 13.84 | 16.91 | 14.03 | 15.77 | 14.22 | 13.78 | 13.12 | 17.37 |
| PI(16:0/16:0) | 0.3 | negative ion | 14.85 | 15.32 | 14.48 | 14.94 | 13.97 | 14.48 | 14.27 | 14.7 | 13.85 |
| GM2(34:1) | 0.25 | negative ion | 14.61 | 14.26 | 13.31 | 14.33 | 14.06 | 14.12 | 16.29 | 15.65 | 13.79 |
| PE(20:1/0:0) | 0.31 | positive ion | 14.58 | 14.23 | 14.89 | 14.18 | 14.36 | 14.68 | 14.86 | 14.44 | 15.41 |
| PC(19:0/0:0) | 0.31 | positive ion | 14.54 | 14.52 | 14.74 | 14.66 | 14.23 | 14.66 | 13.33 | 14.14 | 14.69 |
| PE(16:0/0:0) | 0.34 | negative ion | 14.26 | 14.6 | 15.3 | 14.27 | 13.8 | 14.71 | 14.41 | 14.85 | 15.44 |
| PS(16:0/16:1) | 0.76 | negative ion | 13.78 | 14.05 | 12.39 | 13.83 | 11.82 | 12.83 | 13.19 | 13.91 | 12.01 |
| PG(18:1/0:0) | 0.71 | negative ion | 13.75 | 12.08 | 15.78 | 11.62 | 14.32 | 12.85 | 13.01 | 12.85 | 16.37 |
| PS(18:0/0:0) | 0.15 | negative ion | 13.57 | 12.65 | 14.6 | 11.52 | 13.29 | 12.33 | 13.95 | 13.23 | 14.89 |
| PI(18:0/0:0) | 0.27 | negative ion | 12.94 | 11.3 | 14.44 | 10.45 | 11.38 | 11.24 | 12.49 | 11.14 | 14.69 |
| PI(18:1/0:0) | 0.22 | negative ion | 12.64 | 10.69 | 14.64 | 9.29 | 10.08 | 9.71 | 10.95 | 10.03 | 15.02 |
| PS(18:1/0:0) | 0.12 | negative ion | 12.4 | 11.47 | 12.87 | 10.34 | 11.36 | 11.46 | 12.3 | 11.45 | 12.61 |

Live-BM-EVs: EVs from live *C. neoformans* infected activated BMDMs;

Hk-BM-EVs: EVs from heat-killed *C. neoformans* infected activated BMDMs;

Non-BM-EVs: EVs from activated BMDMs without *C. neoformans* infection;

Hk: heat-killed.
